# Biocompatible Lysine Protecting Groups for the Chemoenzymatic Synthesis of K48/K63 Heterotypic and Branched Ubiquitin Chains

**DOI:** 10.1021/acscentsci.3c00389

**Published:** 2023-07-15

**Authors:** Toshiki Mikami, Sohei Majima, Haewon Song, Jeffrey W. Bode

**Affiliations:** † Laboratory for Organic Chemistry, Department of Chemistry and Applied Biosciences, ETH Zürich, Vladimir-Prelog-Weg 3, CH-8093 Zürich, Switzerland

## Abstract

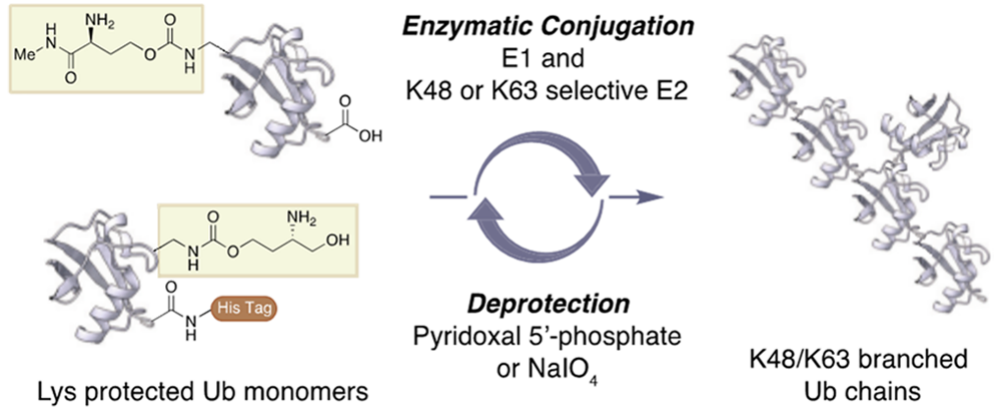

The elucidation of emerging biological functions of heterotypic and branched ubiquitin (Ub) chains requires new strategies for their preparation with defined lengths and connectivity. While *in vitro* enzymatic assembly using expressed E1-activating and E2-conjugating enzymes can deliver homotypic chains, the synthesis of branched chains typically requires extensive mutations of lysines or other sequence modifications. The combination of K48- and K63-biased E2-conjugating enzymes and two new carbamate protecting groups—pyridoxal 5′-phosphate (PLP)-cleavable aminobutanamide carbamate (Abac group) and periodate-cleavable aminobutanol carbamate (Aboc group)—provides a strategy for the synthesis of heterotypic and branched Ub trimers, tetramers, and pentamers. The Abac- and Aboc-protected lysines are readily prepared and incorporated into synthetic ubiquitin monomers. As these masking groups contain a basic amine, they preserve the overall charge and properties of the Ub structure, facilitating folding and enzymatic conjugations. These protecting groups can be chemoselectively removed from folded Ub chains and monomers by buffered solutions of PLP or NaIO_4_. Through the incorporation of a cleavable C-terminal His-tag on the Ub acceptor, the entire process of chain building, iterative Abac deprotections, and global Aboc cleavage can be conducted on a resin support, obviating the need for handling and purification of the intermediate oligomers. Simple modulation of the Ub monomers affords various K48/K63 branched chains, including tetramers and pentamers not previously accessible by synthetic or biochemical methods.

## Abstract 2

Small, polar, bio-orthogonal lysine protecting groups—both readily removed from folded proteins—enable the chemoenzymatic synthesis of branched and heterotypic ubiquitin chains.

## Introduction

Ubiquitin (Ub) is a highly conserved small protein that is found across all eukaryotic organisms. Ub can be conjugated at its C-terminus to a lysine side chain of another protein through the consecutive action of three enzymes: E1 ubiquitin-activating enzymes, E2 ubiquitin-conjugating enzymes, and E3 ubiquitin ligases.^1^ This three-step cascade—ubiquitylation—is one of the most elaborate and versatile post-translational modifications for controlling the stability, localization, and functions of proteins.^2−4^ Ub also forms oligomers of isopeptide bonds via its seven lysine residue side chains or N-terminal amine, resulting in polyubiquitin chains. The resulting nearly unlimited possible Ub chains include homotypic chains, heterotypic chains, and branched Ub chains.

Early studies on the function of poly-Ub focused primarily on homotypic chains such as K48 and K63 chains.^1,5,6^ While the structure and function of typical homotypic chains are reasonably well understood, the existence and physiological role of heterotypic and branched ubiquitin chains have only recently emerged.^7^ Advances in analytical methods have revealed their abundant existence and prolific biological functions,^8−10^ an area of study that continues to evolve rapidly. The physiological functions of some branched ubiquitin chains have been investigated and include enhanced protein degradation by K11/K48 chains,^11−13^ protein degradation of neosubstrates by K29/K48 chains,^14^ amplified NF-κB signaling by K48/K63 chains,^15,16^ and innate immune signaling by M1/K63 chains.^17^ Other ubiquitin chains, including K6/K48, K11/K33, K27/K29, and K29/K33, have been observed in cells using analytical methods, but their biological functions and relevance are not yet understood.^8^

The prevalence of heterotypic and branched ubiquitin chains—coupled with the extreme difficulty in securing homogeneous samples of these large, complex molecules—requires new strategies to prepare Ub chains with defined length and connectivity. Bottom-up approaches to Ub chain synthesis have been pursued by several research groups, including pioneering chemical syntheses of homotypic Ub chains by Pickart,^18^ Fushman,^19^ Brik,^20^ and Komander and Chin.^21^ For the preparation of heterotypic chains, the Liu group has employed native chemical ligation to successfully obtain K11/K48 branched chains. Although they could prepare a Ub hexamer, the branching site was restricted to the C-terminal ubiquitin for the synthesis of V-shaped chains (Figure S1).^22,23^ Enzyme-based construction of branched Ub chains can be achieved by mixing two linkage-selective E2s and mutating Ub lysines to arginines to obtain V-shaped K11/K48 or K48/K63 branched trimers with extensive Lys to Arg mutations.^24,25^ Strieter et al. successfully synthesized branched triubiquitin activity-based probes by a combination of enzymatic conjugation and a Cys modification reaction.^26^ During the revision of this manuscript, Okamoto and co-workers published an approach to ubiquitin chains using a photocleavable lysine protecting group.^27^ Lang et al. have prepared heterotypic and branched Ub trimers without Lys to Arg mutations by sortase-mediated conjugations of Ub monomers containing Gly-Gly-Lys isopeptides.^28^ Fushman and co-workers have incorporated a Boc-protected lysine at K48 by genetic code expansion and enzymatically conjugated the protected Ub to an acceptor Ub with C-terminal blocking such as Ub (1–77) or truncated Ub (1-74) to selectively obtain a Ub dimer.^29^ The resulting dimer was treated with TFA and used as an acceptor for subsequent trimer synthesis. Although this strategy was successfully applied to obtain Ub chains with a defined length without lysine mutations, this approach has not been applied to the synthesis of branched Ub chains.

**Figure 1:**
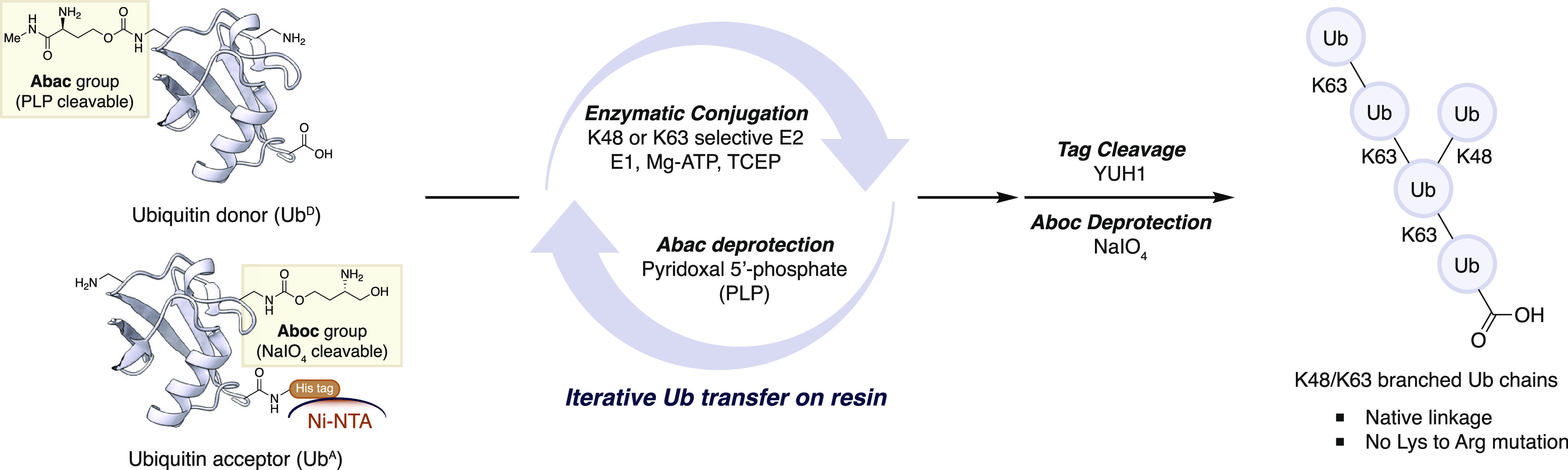
Overview of K48 and K63 branched ubiquitin chain synthesis using Abac (PLP-cleavable) and Aboc (NaIO_4_-cleavable) lysine protecting groups.

## Results and Discussion

### Design and Synthesis of Orthogonal Protecting Groups

Due to their prevalence and putative role in a number of biological processes, including a role in viral uncoating processes currently being investigated as part of collaborations in our lab,^30−33^ we initially targeted the synthesis of defined, branched K48/K63 Ub chains. Our intended chemoenzymatic approach required at least two transient protecting groups for Ub lysines. Although we initially considered established solutions including photolabile protecting groups^34^ (e.g., Nvoc) or azidolysines, initial studies on Ub chain synthesis and handling of Ub monomers with these hydrophobic side chains indicated this would not be a viable route to longer chains. An alternative approach investigated in our group—using lysine homologues as masking group—unfortunately did not deter undesired chain formation,^35^ although many E2 and E3 enzymes show strong preference for a native lysine acceptor.^36^ We therefore sought alternative masking groups that maintained the overall properties of folded Ub monomers by incorporating a basic primary amine in the protecting groups themselves. Further requirements included compatibility with Fmoc-SPPS and deprotection from folded, unprotected Ub chains under mild conditions.

**Figure 2:**
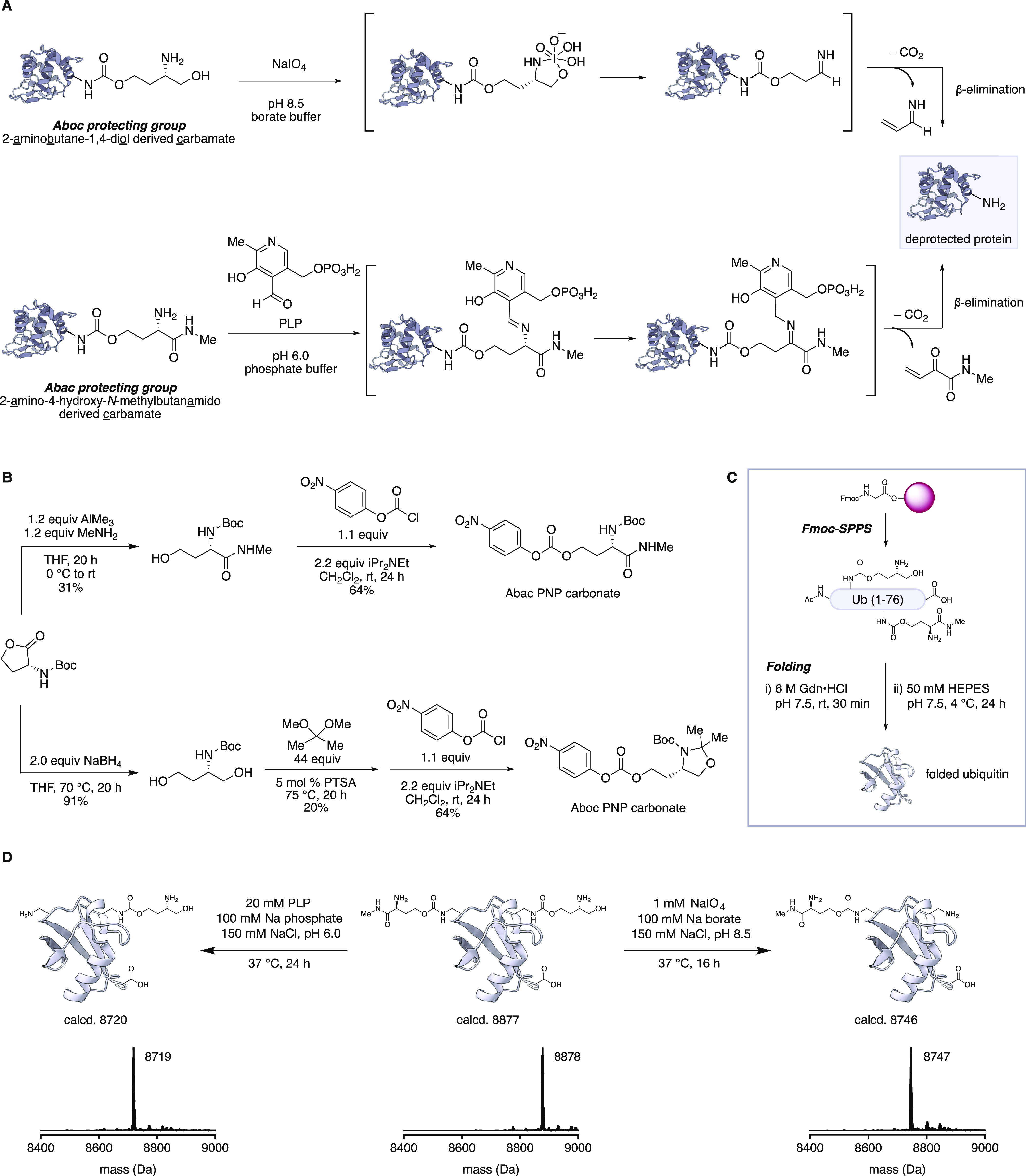
Design and synthesis of biocompatible lysine side chain protecting groups. (A) Plausible deprotection reactions of Aboc and Abac groups by NaIO_4_ and PLP, respectively. (B) Synthesis of Aboc and Abac carbonates with acid labile protecting groups. (C) Preparation of Ub^D^ monomer. (D) Orthogonal deprotections on folded Ub.

Inspired by a number of reported caging strategies that operate via induced β-elimination reactions from a carbonyl group formed by a chemical or enzymatic transformation,^37−39^ we designed two novel carbamate protecting groups containing primary amines (Figure 2A). In the first, an aminobutanol carbamate (Aboc group) would generate an aldehyde upon periodate treatment, followed by subsequent β-elimination to release the lysine amine. Importantly, oxidation of biomolecules with NaIO_4_ has long been known as selective and compatible with unprotected proteins.^40,41^ The requisite protecting group and its incorporation into (*S*)-Fmoc-lysine was readily achieved on a multigram scale, starting from (*S*)-Boc-homoserine lactone (Figure 2B).

For the second protecting group, we exploited a reaction of α-amino acid residues with pyridoxal 5′-phosphate (PLP), a natural cofactor for transamination reactions.^42,43^ PLP converts an α-amine into an imine, which in the case of homoserine-derived carbamates leads to β-elimination. We designed the Abac protecting group, consisting of an aminobutanamide moiety attached to a Lys side chain via a carbamate (Figure 2A). This protecting group and the corresponding lysine monomers could be prepared from (*S*)-Boc-homoserine lactone in excellent yields (Figure 2B).

### Lysine Deprotections on Folded Ubiquitin

As an initial test of the suitability of these protected lysines for SPPS, Ub folding, and deprotection on folded proteins, we prepared K48-Aboc/K63-Abac Ub donor (Ub^D^) **2** by automated peptide synthesis of full length Ub (1–76) using the procedure developed by Ovaa, which employed several pseudoproline and Dmb dipeptides.^44^ The N-terminal methionine (Met1) of Ub was substituted by norleucine to avoid oxidation, and the N-terminal amine was acetylated to prevent reaction with PLP. The incorporation of lysine monomers bearing these new protecting groups proceeded smoothly to afford the expected linear Ub monomers.

Purified linear Ub^D^**2** was dissolved in 6 M Gdn·HCl, and the resulting solution was dialyzed into HEPES buffer for folding. The deprotections and orthogonality of the protecting groups were tested by selective removal of each protecting group from the folded Ub^D^**2** (Figure 2C). Optimal conditions for deprotection of the Aboc group on Ub^D^**2** were determined to be 1 mM NaIO_4_ in Na borate buffer at pH 8.5. The oxidative cleavage was complete within 10 min, but the β-elimination required a basic pH and a longer reaction time for completion. Tris buffer (20 mM, pH 8.5) was added to the reaction mixture after 10 min to quench excess periodate and raise the pH. The addition of a secondary amine—such as pyrrolidine—accelerated the elimination step,^45^ although this was not strictly necessary and could be omitted. Importantly, the Abac group on K63 remained intact under these deprotection conditions even after 16 h.

The PLP-mediated cleavage of the Abac group was tested by using the same Ub monomer. After 24 h of incubation with 20 mM PLP in Na phosphate buffer at pH 6.0, we confirmed complete deprotection with no side products. In this reaction, we found that the β-elimination required a slightly acidic pH (below pH 6.0); otherwise, the reaction stopped before the elimination step. The Aboc group did not react with PLP and showed no decomposition throughout the reaction.

### Construction of Ub Chains by Iterative E2-Mediated Conjugations and Deprotections

The chemoenzymatic construction of Ub chains requires two distinct types of Ub monomer—donors (Ub^D^) and an initial acceptor (Ub^A^). We aimed to synthesize K48/K63 branched chains, as these are the most observed linkages. We also sought to take advantage of well-studied K48- and K63-biased E2 enzymes, some of which show excellent lysine selectivity. Linear K48 chains have been prepared using Ub (1–77) as an acceptor, Ub (K48C) as a donor, and Ube2K as the E2-conjugating enzyme. Deblocking of D77 by yeast ubiquitin hydrolase 1, YUH1, exposes the native G76, and the resulting Ub_2_ works as a donor in the following elongation step. Alternatively, alkylation of the cysteine side chain by aziridine affords the Lys analogue, and the Ub_2_ serves as an acceptor for the next conjugation. A similar approach using Ubc13/Mms2 affords linear K63-linked chains.^46^

**Figure 3:**
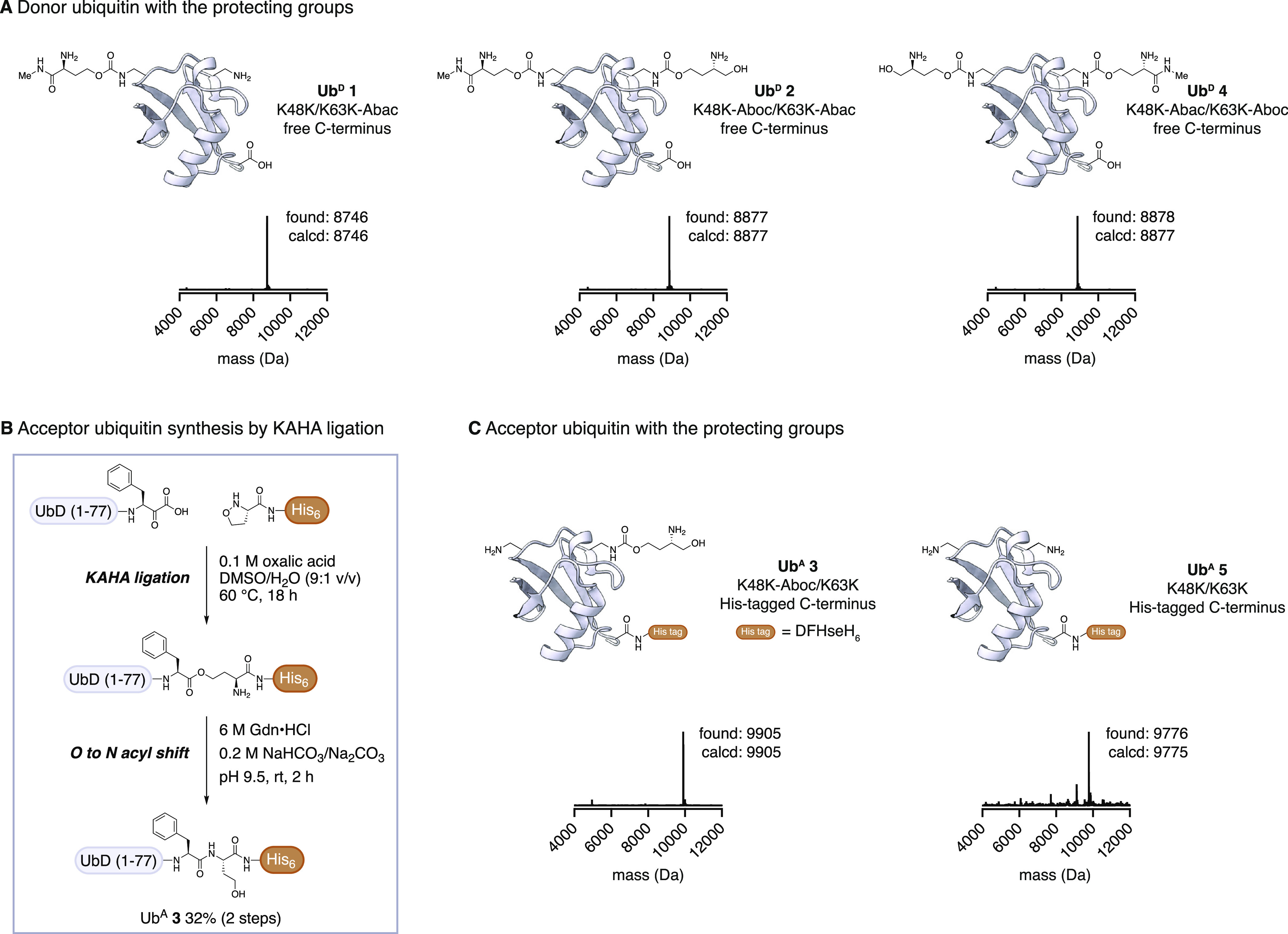
Chemical synthesis of protected Ub donors and acceptors. (A) Donor ubiquitins prepared by Fmoc-SPPS. Deconvoluted mass spectra of the purified proteins are shown. (B) Synthesis of Ub^A^**3** by Fmoc-SPPS and KAHA ligation. (C) Deconvoluted mass spectra of the purified proteins. Ub^A^**5** was prepared from Ub^A^**3** by treatment with NaIO_4_.

Our approach toward unanchored, branched Ub chains requires a Ub^A^ with a blocked—but ultimately cleavable—C-terminus and transient protection on either K48 or K63. As a blocking strategy, we employed YUH1 to cleave a C-terminal RGG-DH6 extension.^47^ The inclusion of a C-terminal (His)_6_ tag would facilitate purification and immobilization. Unfortunately, SPPS of this longer Ub^A^ resulted in a low yield and many side products. To circumvent this problem, we synthesized Ub^A^**3** by KAHA ligation.^48^ A Ub C-terminal α-ketoacid, which was extended by D77 and F78 (as the α-ketoacid), was successfully obtained utilizing our previously developed α-ketoacid-forming linker.^49^ The Ub α-ketoacid was coupled with an Opr-His_6_ peptide (Opr, (*S*)-5-oxaproline) in DMSO/aq oxalic acid (9:1 v/v). After O to N acyl shift in a basic buffer, the product Ub^A^**3** was purified by HPLC, and the mass was confirmed by LC-MS (Figure 3B,C).

The Ub^D^ monomers require a free C-terminus and blocked K48 and/or K63 Lys side chain(s); for these studies, we selected Ub^D^**1** and Ub^D^**2**. As a result of the expected site selectivity of certain E2-conjugating enzymes, we protected only K63 or K48 residues, leaving K6, K11, K27, K29, and K33 as native lysines. The peptide syntheses of Ub^D^**1** and other Ub^D^s were successfully carried out by analogous procedures to those used for monomer Ub^D^**2** (Figure 3A).

To prepare K48/K63 branched chains, we initiated the synthesis by constructing a K63 linear chain followed by a K48 branching reaction with Ube2K, which has a UBA domain that preferentially binds to K63 chains and catalyzes K48 chain formation.^50^ The linear K63 chain building was achieved by using Ubc13/Mms2, a K63-biased heteromeric dimer E2, along with Uba1 (E1) and ATP. The PLP-cleavable protecting group (Abac) on K63 of Ub^D^ restricted K63 elongation by blocking the active lysine side chain for further oligomerization. This strategy allowed us to perform monoubiquitylation in each conjugation cycle and to use different ubiquitin donors, K48K/K63K-Abac Ub^D^**1** and K48K-Aboc/K63K-Abac Ub^D^**2**. We could obtain different branched chains by changing the order of Ub^D^**1** and **2** because Ub^D^**1** is the only position where the branching reaction occurs with Ube2K.

**Figure 4:**
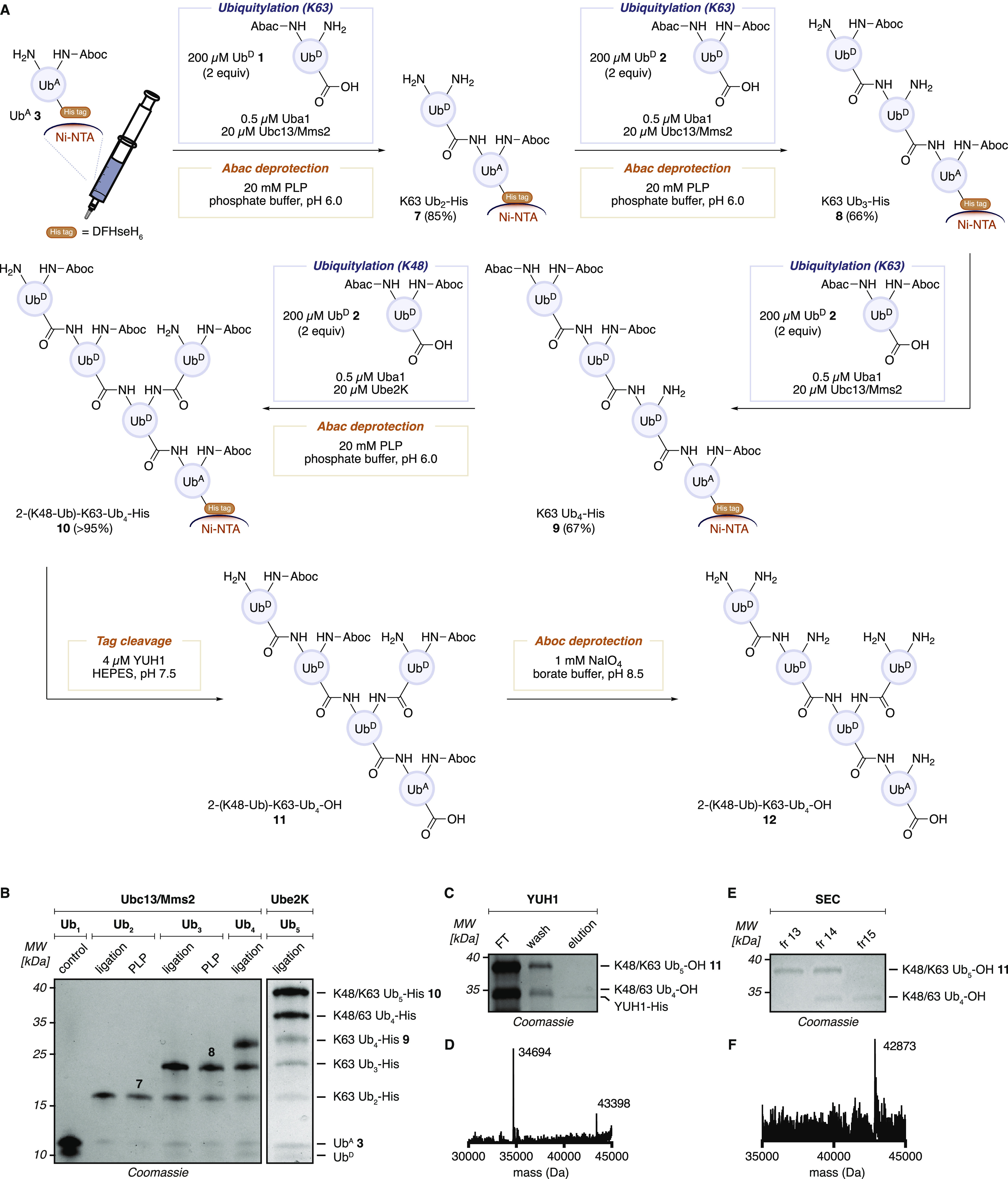
Branched Ub chain construction cycle. (A) Graphical scheme of resin-supported Ub chain synthesis. Conversions were calculated based on densitometry of the SDS-gel after Coomassie staining. (B) SDS-PAGE gel of Ub chain elongation (Coomassie staining). After each reaction (conjugation and PLP deprotection), a small amount of resin was taken, washed, and eluted. The gel shows a stepwise growth of the Ub chain. (C) SDS-PAGE gel of enzymatic C-terminal tag cleavage (Coomassie staining). Treatment of the Ub chain on resin with YUH1 cleaved the Ub chains from the resin (FT: flow through). (D) Deconvoluted mass spectrum of the flow through fraction after tag cleavage by YUH1. Two major products (Ub_5_ and Ub_4_) were observed. Calcd for Aboc-protected Ub_5_: 43 397, Calcd for Aboc-protected Ub_4_: 34 695. (E) SDS-PAGE gel of fractions from size-exclusion chromatography (Coomassie staining). Ub_5_ and Ub_4_ were separated by size-exclusion chromatography. (F) Deconvoluted mass spectrum of the crude mixture after NaIO_4_ cleavage. Calcd for Aboc deprotected Ub_5_: 42 872.

The synthesis of Ub chains requires sequential conjugation and deprotection as illustrated in Figure 4A. We adopted the recently proposed nomenclatures for branched ubiquitin from Kulath et al., which provides a method of describing heterotypic ubiquitin chains.^51^ In our synthesis, the removal of excess Ub^D^ and the E2-conjugating enzymes after the conjugation as well as buffer exchange before each conjugation/deprotection are inevitable. To facilitate the synthesis and avoid chromatographic purification after each conjugation or deprotection, we implemented an on-resin synthesis of ubiquitin chains. This was made possible by the C-terminal extension including a His_6_ tag on Ub^A^**3**, which could be immobilized onto Ni-NTA agarose resin. The subsequent chemoenzymatic Ub transfer and Abac deprotection steps could be conducted with an immobilized Ub acceptor or a growing chain. This facilitated the removal of non-tagged Ub^D^ and E2s from the mixture and enabled buffer exchange by simple washing of the resin, analogous to SPPS.

In the on-resin chain-building reactions, 0.2 μmol of Ub^A^**3** (1 equiv) was loaded on Ni-NTA agarose resin and conjugated with a K48-unprotected Ub^D^**1**; the unprotected K48 is subsequently used as the branching site. The conjugation was carried out using 0.5 μM Uba1, 20 μM Ubc13/Mms2, 200 μM (2 equiv) Ub^D^, 5 mM Mg-ATP, and 0.2 mM TCEP in 50 mM HEPES, 150 mM NaCl, pH 7.5, at 37 °C for 16 h. The incorporation of Lys protecting groups may affect enzyme recognition, as noted by studies showing that simple acylation of Lys side chains in Ub affect chain-building efficiency.^52^ We postulated that the protecting groups on Ub^A^ K48 might impair the binding of Mms2, one of the E2 enzymes for K63 building.^53,54^ Nevertheless, ubiquitylation proceeded with 60–90% conversion according to densitometry of the SDS-PAGE gel after staining (Figure 4B).

After Ub attachment, the resin was washed, and Abac deprotection solution—composed of 20 mM PLP in 100 mM Na phosphate, pH 6.0, 150 mM NaCl—was added. The mixture was agitated at 37 °C for 20 h to ensure complete Abac removal. The conjugation and deprotection cycles were repeated until K63 Ub_4_**9** was obtained (Figures 4A, S2). At this point, K63 Ub_4_ has four K48 side chains, and only the second Ub from the C-terminus is available for conjugation. The branching reaction was conducted using 20 μM K48-biased Ube2K (instead of Ubc13/Mms2) and 200 μM Ub^D^**2**. The generation of branched K48/K63 Ub_5_-His **10** was confirmed by SDS-PAGE (Figure 4B). The analysis showed the mixture also contained some amount of branched Ub_4,_ which arises from incomplete coupling of the second or third ubiquitylation with Ubc13/Mms2. After PLP-mediated deprotection, the His-tag of the branched chains was cleaved and eluted from the resin by YUH1, which preferentially cleaves C-terminal amide bonds of a Ub chain over the isopeptide bonds connecting Ub chains and releases the C-terminal carboxylic acid (Figure 4E). The resulting unanchored Ub_5_ was separated from the remaining Ub_4_ by size-exclusion chromatography (SEC), and the isolated K48/K63 Ub_5_-OH **11** was subjected to deprotection of the four Aboc groups (Figure 4D). For this global deprotection, Ub_5_-OH **11** in 50 mM HEPES and 150 mM NaCl at pH 7.5 was treated with a 1 mM solution of NaIO_4_ in 200 mM Na borate buffer (pH 8.5). After 30 min, 50 mM Tris-HCl (pH 7.5) was added to quench the excess NaIO_4_, and the mixture was further agitated for 16 h at 37 °C. To confirm complete deprotection, the obtained branched pentamer was lyophilized and desalted for LC-MS analysis. We were pleased to find the corresponding intact mass of the fully deprotected branched K48/K63 Ub_5_**12**, containing all native lysine residues (Figure 4F).

**Figure 5:**
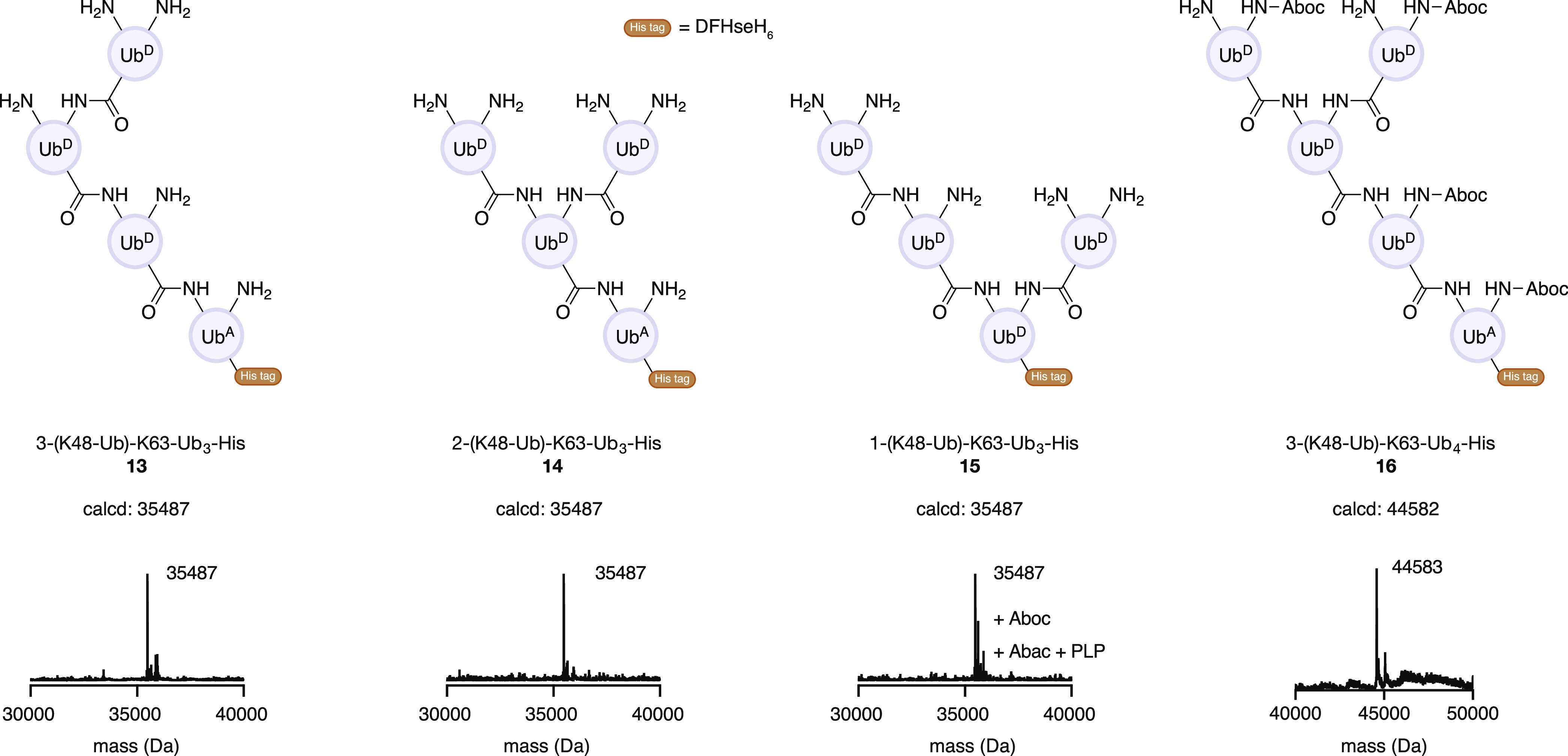
Heterotypic and branched Ub chains synthesized. Ub tetramers were synthesized by slightly modified conditions. (See Supporting Information for detailed procedures and SDS-PAGE gels.)

This method enables us to obtain heterotypic and branched Ub chains with different conjugation sites by simply swapping the protecting groups of K48 and K63. Heterotypic tetraubiquitin **13**, two different branched Ub tetramers **14** and **15**, and another branched Ub pentamer **16** were prepared from just three Ub monomers (Figure 5, estimated conversions for each conjugation step are included in the Supporting Information, Figures S3 to S8). In contrast to other approaches, this method accepts any branching site (from distal ubiquitin to middle and proximal ubiquitins) by simply changing the Ub^A^ or Ub^D^.

### Safety Statement

No unexpected or unusually high safety hazards were encountered.

## Discussion

General strategies for the preparation of branched Ub chains should accommodate branching at the proximal, middle, and distal positions, some of which have been challenging to prepare with previously reported methods. Our approach of employing “temporary” (Abac, PLP-cleavable) and “permanent” (Aboc, NaIO_4_-cleavable) lysine protecting groups realized the syntheses of Ub_4_ and Ub_5_ branched chains with these topologies. This work also takes advantage of K48- and K63-biased E2 enzymes, suggesting that this strategy will be extendable to other branching sites for which biased E2-conjugating enzymes are known, such as K11.^13,55^ We expect that other atypical linkages would also be obtained by limiting linkage-promiscuous E2 enzymes, such as the Ubc5 family,^56^ to the synthesis of specific atypical Ub chains by restricting the available conjugation sites by protection of the other lysine residues.^57^ Alternatively, E3 ligases and linkage-specific DUBs may expand the scope, as previously described for K6 chain synthesis.^58^

Ub chain synthesis, in general, requires multiple chromatographic purifications and can be laborious. The resin-supported synthesis implemented here rendered the preparation simpler and less time-demanding and should be amenable to automation. We found that the efficiency of conjugation on solid support was comparable to that in the solution phase. To construct even longer Ub chains, improvements in the efficiency of the Ub transfer step, possibly through the use of chimeric E2-E3 proteins, will be advantageous.^59^ As the Ub chain length increases, isolating Ub_*n*_ from Ub_*n*–1_ by size-exclusion chromatography becomes more difficult. At present, the iterative Abac deprotection requires longer reaction time as the chain length increases, an aspect that can be improved by fine-tuning this new class of carbamate protecting groups. Finally, although these initial studies were conducted on small scale, there is no inherent limitation to larger scale chain synthesis. The synthetic Ub can be prepared on multimilligram scale, and the requisite enzymes are available from *E. coli* expression.

## Conclusion

Branched Ub chains add layers of complexity to understanding the ubiquitin code. Contemporary interest in the function of Ub chains includes studies of how branched chains interact with associated reader proteins and the construction of antibodies for the detection and isolation of specific Ub branches. The lack of synthetic approaches to homogeneous, heterotypic Ub chains containing all seven native lysine residues currently limits further studies. In this Article, we documented the development of two new orthogonal lysine protecting groups that can be removed from folded Ub chains and maintain the overall charge state of Ub monomers and chains. The protecting groups are easily prepared by chemical synthesis and incorporated into proteins by Fmoc-SPPS. The resulting fully synthetic, orthogonally protected Ub monomers could be assembled into heterotypic/branched chains using a solid support that minimizes isolation and purification of the growing Ub chains. The combination of these advances enables the preparation of Ub chains with branching at the proximal, middle, and distal positions.

## Supporting Information Available

Additional figures, methods, characterization data by NMR, IR, HPLC, mass spectrometry, and SDS-PAGE gels (PDF)
Transparent Peer Review report available (PDF)
