# Supplementary material for: Biocompatible Lysine Protecting Groups for the Chemoenzymatic Synthesis of K48/K63 Heterotypic and Branched Ubiquitin Chains
Source: ACS Cent Sci. 2023 Jul 15;9(8):1633–41. doi: 10.1021/acscentsci.3c00389 (PMC10450881; doi:10.1021/acscentsci.3c00389)
Supplement: Supplementary file 1 — oc3c00389_si_001.pdf [file oc3c00389_si_001.pdf]

Supporting Information

Biocompatible lysine protecting groups for the chemoenzymatic synthesis of K48/K63 heterotypic and branched ubiquitin chains

Toshiki Mikami‡, Sohei Majima‡, Haewon Song, Jeffrey W. Bode

Department of Chemistry and Applied Biosciences
ETH Zürich
Vladimir Prelog Weg 3
8093 Zürich, Switzerland

E-mail: bode@org.chem.ethz.ch

Table of Contents

1 Supplementary figures 4

1.1 Summary of previously developed methods 4
1.2 Supplementary information for Figure 4 5
1.3 Supplementary figures for Figure 5. 6
1.4 Full gels used in Figures 12

2 General Methods 13

2.1 Reagents 13
2.2 NMR 13
2.3 Fmoc Solid phase peptide synthesis (Fmoc-SPPS) 13
2.4 Coupling of special amino acids 14
2.5 Resin cleavage procedures 14
2.6 KAHA ligation[11] 15
2.7 Folding of ubiquitin variants 15
2.8 High-Performance Liquid Chromatography 15
2.9 LC-MS measurement 16
2.10 High-resolution Mass Spectroscopy 16
2.11 Reagents for protein expression 17
2.12 Gel electrophoresis 17
2.13 Gelcode blue staining 18
2.14 Fast Protein Liquid Chromatography (FPLC) 18
2.15 Protein quantification and UV-Vis spectroscopy 18

3 Synthesis of Abac and Aboc protected Fmoc-Lys 19

3.1 Synthesis of homoserine lactone **S2** 19
3.2 Synthesis of Boc Hse NHMe **S3** 19
3.3 Synthesis of Abac-PNP carbonate **S4** 20
3.4 Synthesis of Abac protected Fmoc-Lys-OH **S5** 20
3.5 Synthesis of diol **S6** 21
3.6 Synthesis of **S7** 22
3.7 Synthesis of Aboc-PNP carbonate **S8** 22
3.8 Synthesis of Aboc protected Fmoc-Lys-OH **S9** 23

4 Synthesis of Aboc/Abac protected Ub monomers by Fmoc-SPPS 24

4.1 Ub donor (Ub^D) synthesis 24

4.2 Ub^A **3** synthesis by KAHA ligation 29

5 Protein expression 32

5.1 Expression of Uba1 32
5.2 Expression of Ubc13/Mms2 32
5.3 Expresssion of Ube2K 33
5.4 Expression of GST-3C protease[18] 33
5.5 Expression of YUH1 33

6 Ubiquitin chain elongation 35

6.1 Enzymatic ligation 35
6.2 PLP mediated deprotection 35
6.3 $NaIO_4$ mediated deprotection 36
6.4 YUH1 tag cleavage (Figure 4) 36
6.5 Estimation of ubiquitylation conversions 36

7 NMR spectrum 37

8 Reference 42

1 Supplementary figures

1.1 Summary of previously developed methods

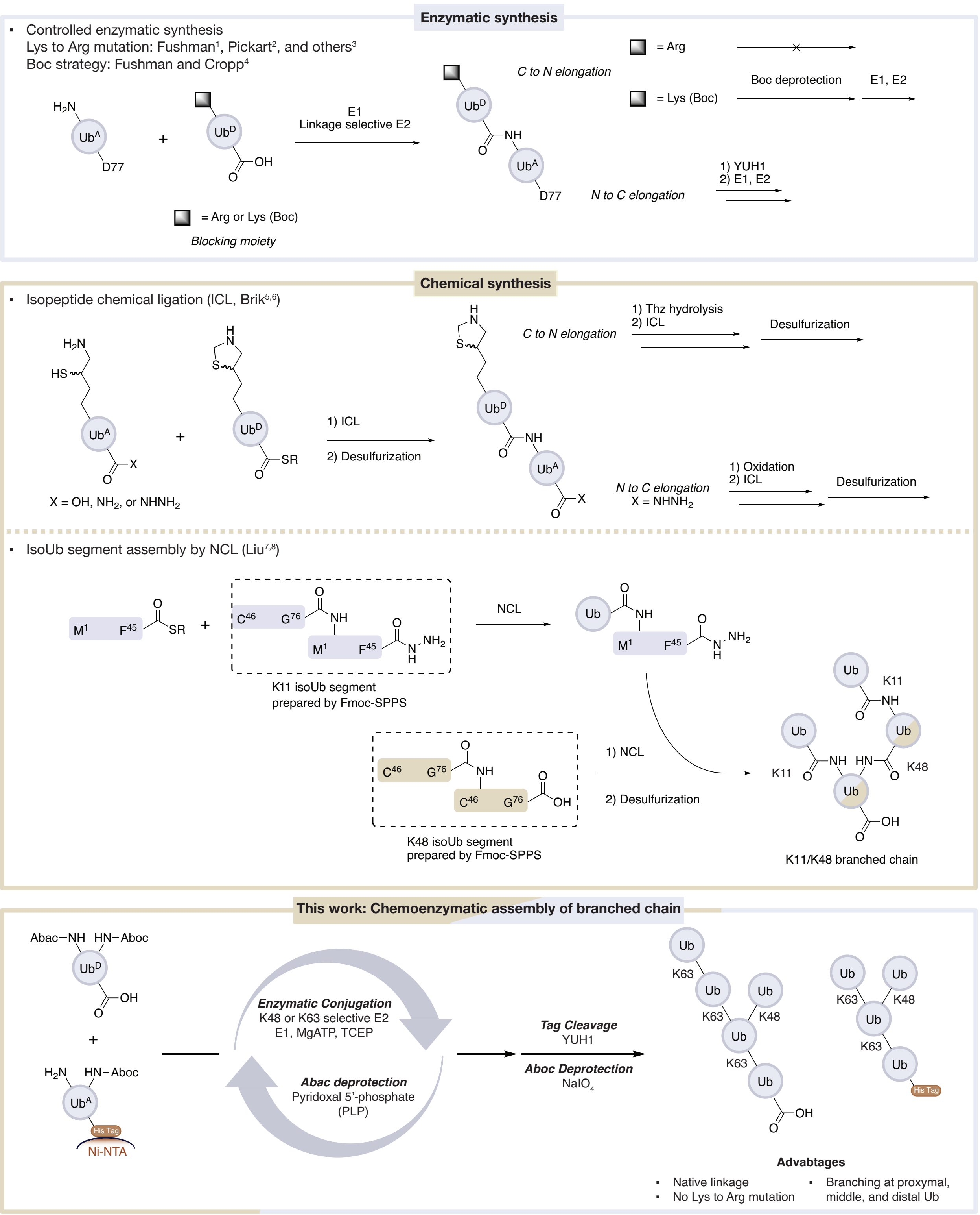

Figure S1. Some of the existing enzymatic and chemical methods are highlighted together with this work.[1–8] Other methods such as Alloc protection by Fushman[9] and GOPAL strategy by Chin and Komander[10] are not shown.

1.2 Supplementary information for Figure 4.

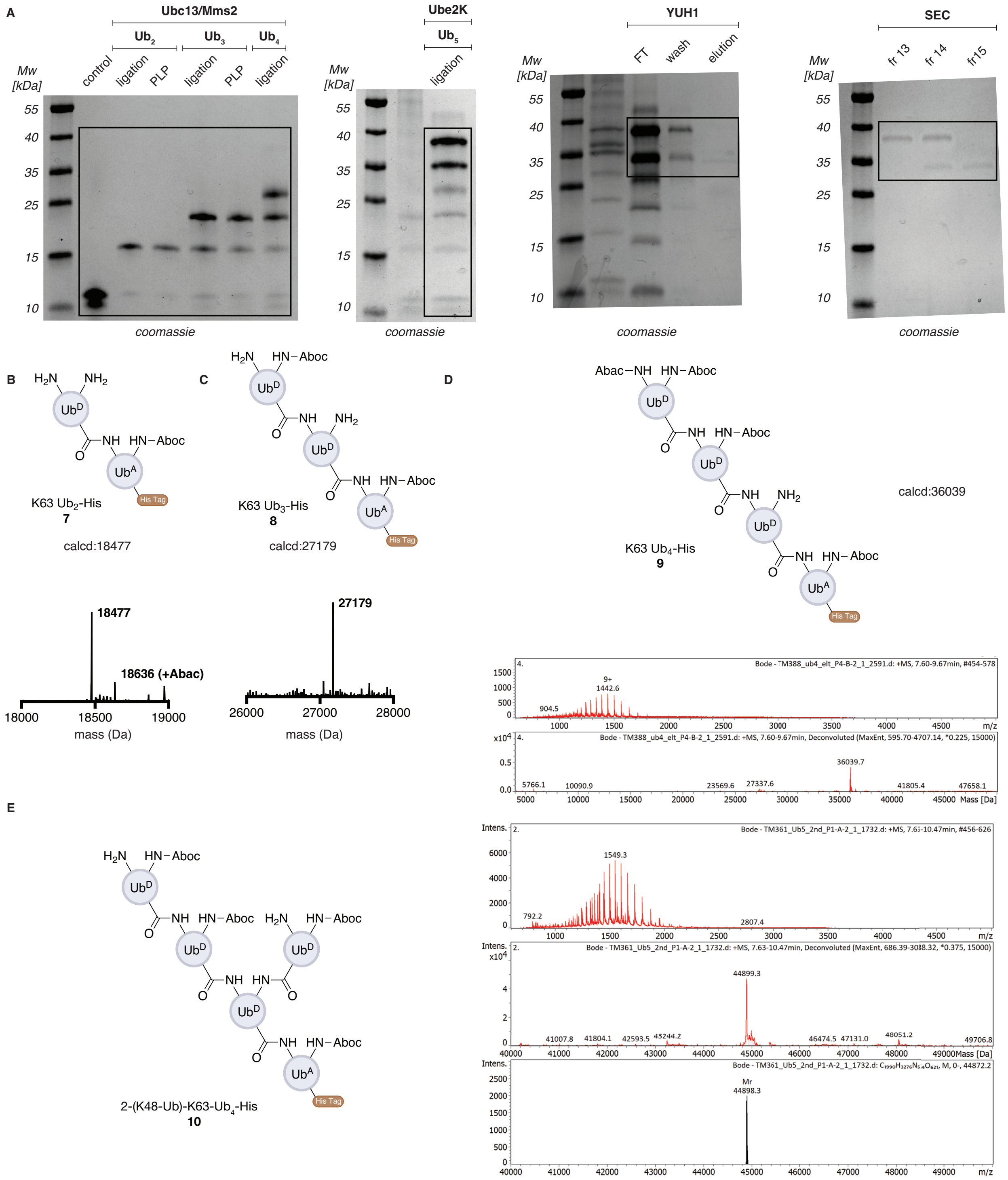

Figure S2. Additional data for Figure 4. (A) SDS-PAGE gel used in Figure 4 before masking. (B) Deconvoluted mass obtained after Abac deprotection. The mass corresponds to Ub_2 **7** with one Aboc group, which suggests the branching site is different from the one in Figure S3. (C) Deconvoluted mass spectrum of Ub_3 **8** after Abac deprotection. Spectra of panel B and C were obtained using LC-MS method A. (D) LC-MS result of the crude after ligation. Top: obtained mass spectrum, Bottom: Deconvoluted mass spectrum (E) LC-MS result of Ub_5 **10** after ligation and Abac deprotection. Top: observed mass spectrum, Middle: Deconvoluted mass spectrum, Bottom: calculated mass for the protected Ub_5. Spectra of panel D and E were obtained using LC-MS method B.

1.3 Supplementary figures for Figure 5.

1.3.1 Synthesis of branched Ub pentamer **16** with a different branching point

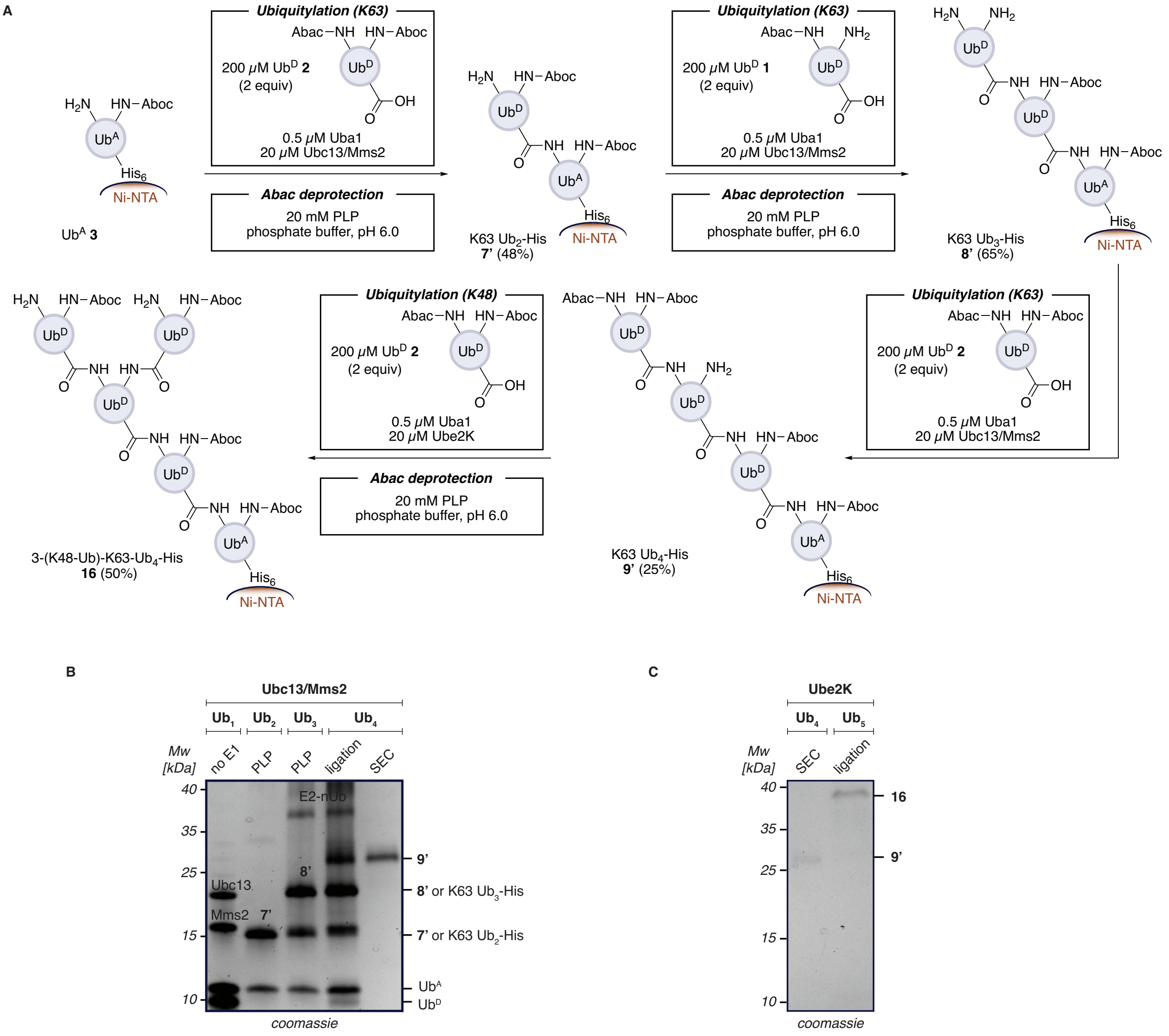

Figure S3. Branched Ub chain construction cycle. (A) Graphical scheme of on-resin Ub chain synthesis. Conversions were calculated based on densitometry of the SDS-gel after Coomassie staining. (B) SDS-PAGE gel of Ub chain elongation (Coomassie staining). After each reaction (ligation and Abac deprotection), a small amount of resin was sampled, washed, and eluted with elution buffer. The gel shows a stepwise growth of the Ub chain. (C) SDS-PAGE gel of ubiquitylation by Ube2K (Coomassie staining). The isolated linear Ub_4 **9'** was converted to branched Ub_5 **16**.

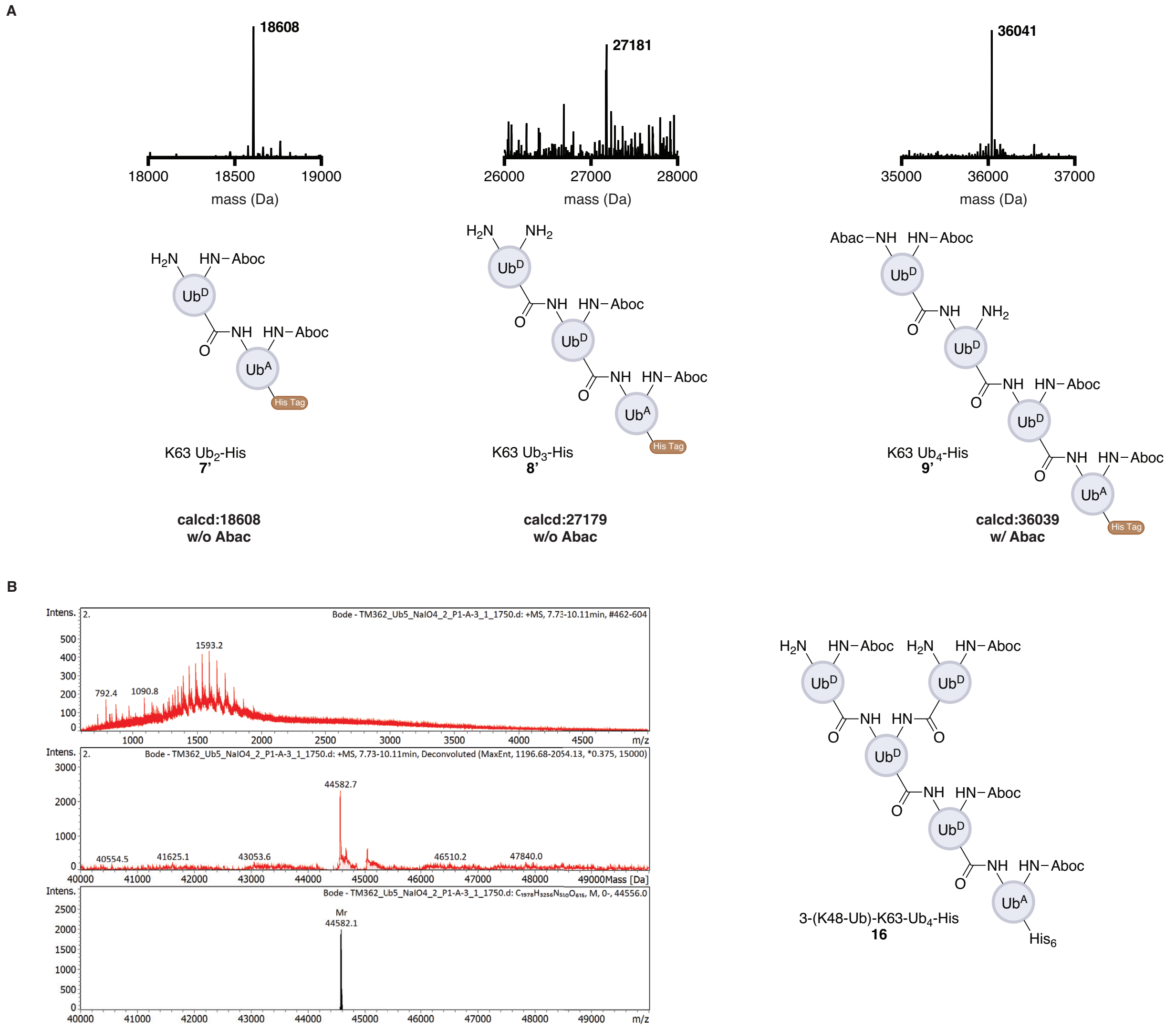

Figure S4. Mass spectra of Ub oligomers shown in Figure S3. (A) Deconvoluted mass spectra of Ub_2 **7’**, **8’**, and **9’** after Abac deprotection. The mass corresponds to Ub_2 **7’** with two Aboc groups, which supports that the branching site is different from the one in Figure 4. B) Deconvoluted mass spectrum of branched Ub_5 **16** after Abac deprotection. The spectrum was obtained using LC-MS method B.

1.3.2 Synthesis of heterotypic Ub tetramer **13**

The synthesis was conducted following the procedures described in Section **6.1.2**

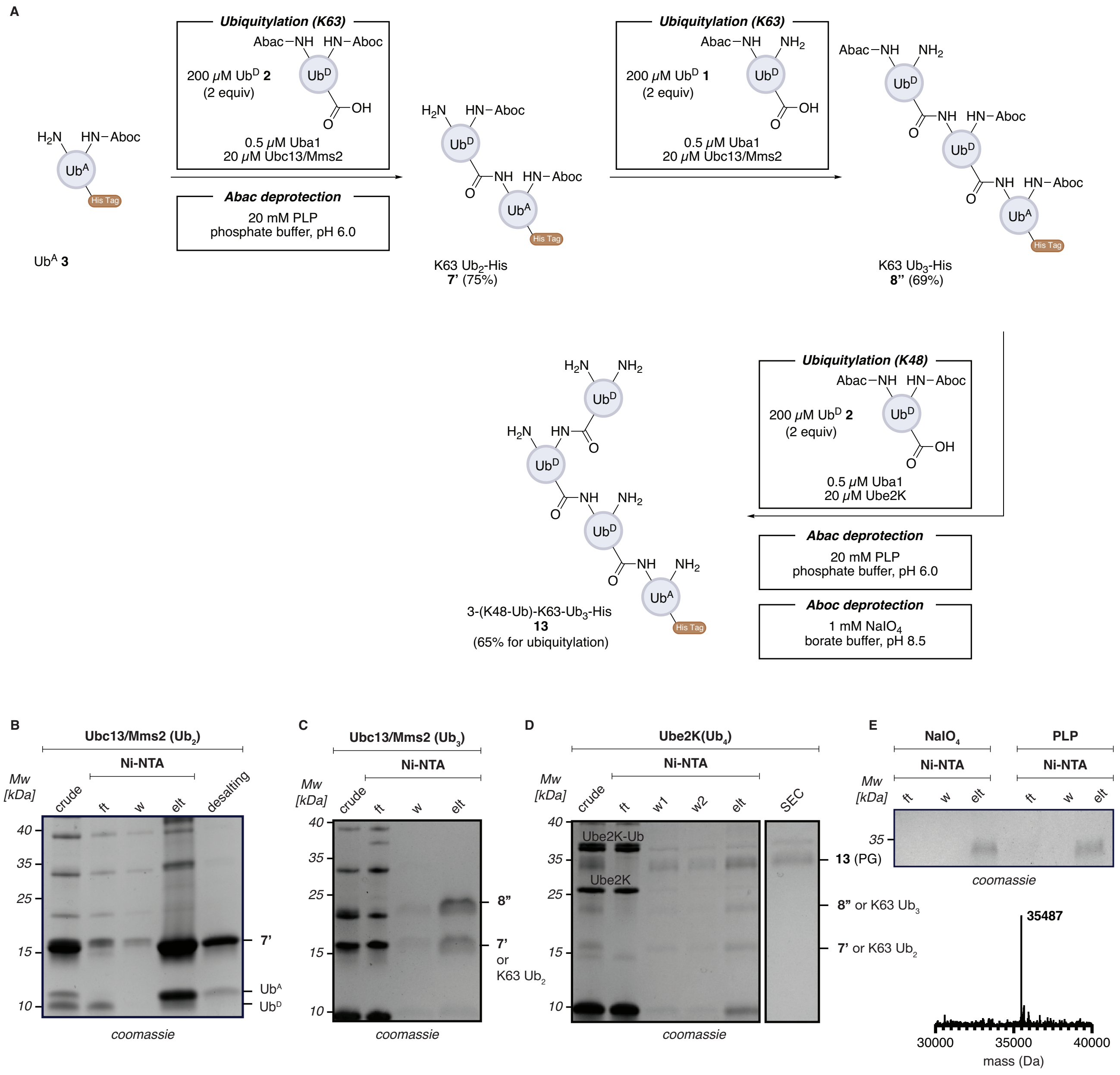

Figure S5. Branched Ub chain construction cycle. (A) Graphical scheme of Ub chain synthesis. Conversions were calculated based on densitometry of the SDS-gel after Coomassie staining. (B) SDS-PAGE gel of dimer synthesis after Coomassie staining (ft: flow through, w: wash, elt: elution). (C) SDS-PAGE gel of trimer synthesis after Coomassie staining (ft: flow through, w: wash, elt: elution). (D) SDS-PAGE gel of tetramer synthesis after Coomassie staining (ft: flow through, w1: wash fraction 1, w2: wash fraction 2, elt: elution, **13** (PG): Ub_4 **13** before Aboc and Abac deprotection). (E) Top: On-resin Aboc and Abac deprotection (ft: flow through, w: wash, elt: elution). The product was only observed in the elution fractions indicating there was no observable sample loss from the resin. Bottom: Deconvoluted mass spectrum of branched Ub_4 **13** after two deprotections. Spectrum was obtained using LC-MS method B.

1.3.4 Synthesis of branched Ub tetramer **14** with a branching point at the middle Ub

The synthesis was conducted following the procedures described in Section **6.1.2**

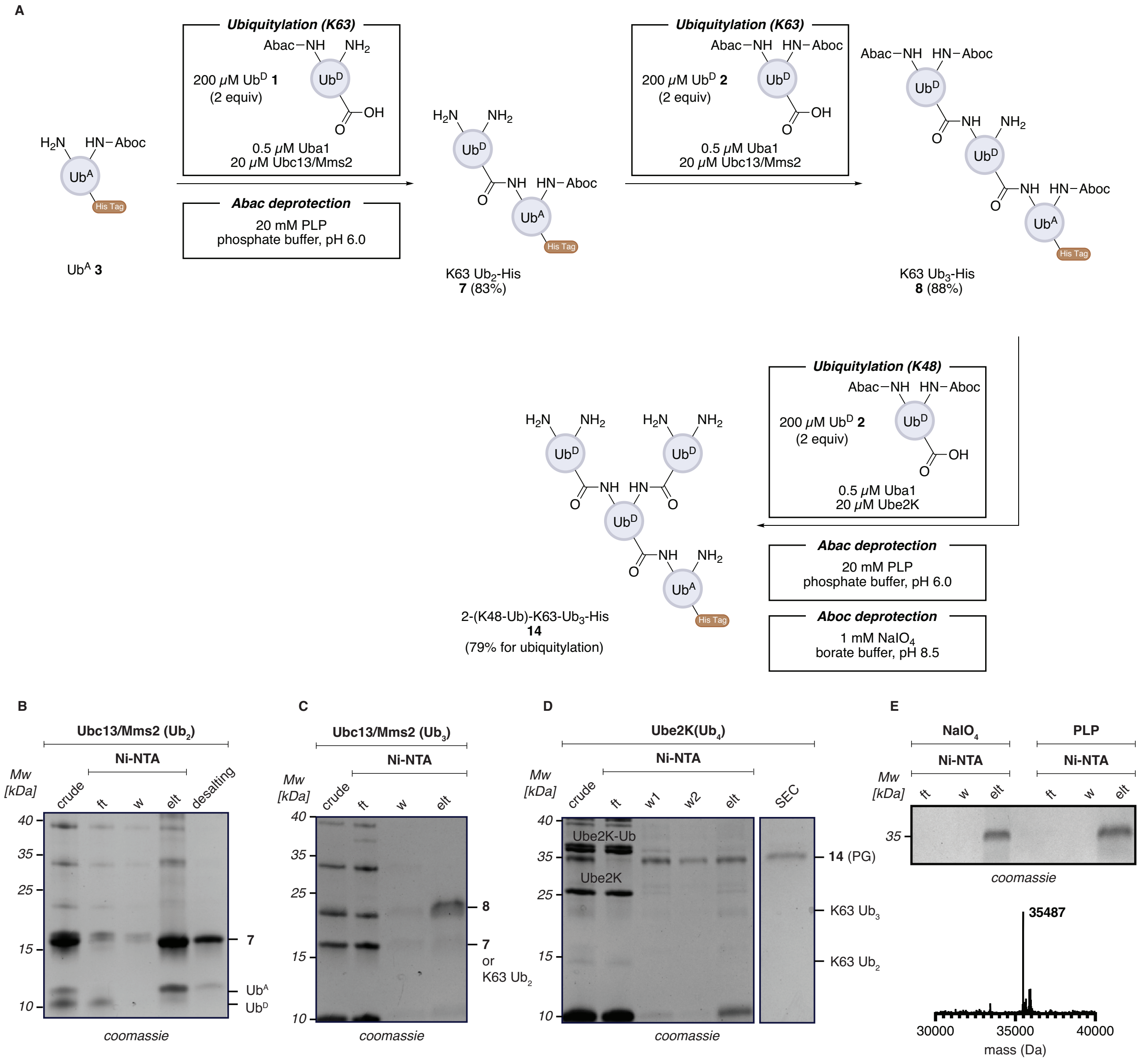

Figure S6. Branched Ub chain construction cycle. (A) Graphical scheme of Ub chain synthesis. Conversions were calculated based on densitometry of the SDS-gel after Coomassie staining. (B) SDS-PAGE gel of dimer synthesis after Coomassie staining (ft: flow through, w: wash, elt: elution). (C) SDS-PAGE gel of trimer synthesis after Coomassie staining (ft: flow through, w: wash, elt: elution). (D) SDS-PAGE gel of tetramer synthesis after Coomassie staining (ft: flow through, w1: wash fraction 1, w2: wash fraction 2, elt: elution, **14** (PG): Ub_4 **14** before Aboc and Abac deprotection). (E) Top: On-resin Aboc and Abac deprotection (ft: flow through, w: wash, elt: elution). The product was only observed in the elution fractions indicating there was no observable sample elution from the resin. Bottom: Deconvoluted mass spectrum of branched Ub_4 **14** after two deprotections. Spectrum was obtained using LC-MS method B.

1.3.6 Synthesis of branched Ub tetramer **15** with a branching point at the proximal Ub

The Ub^A **3** was subjected to periodate cleavage reaction as described to obtain Ub^A **5.**

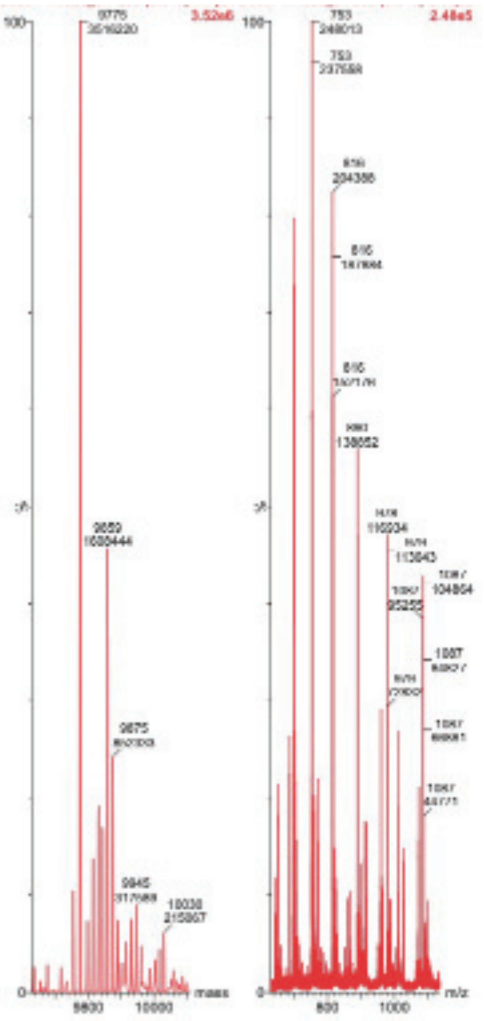

Figure S7. Left**:** Deconvoluted mass spectrum after overnight reaction. Right: Observed mass spectrum before deconvolution. Calcd for deprotected product: 9775. Spectrum was obtained using LC-MS method A.

The synthesis was conducted following the procedures described in Section **6.1.2**

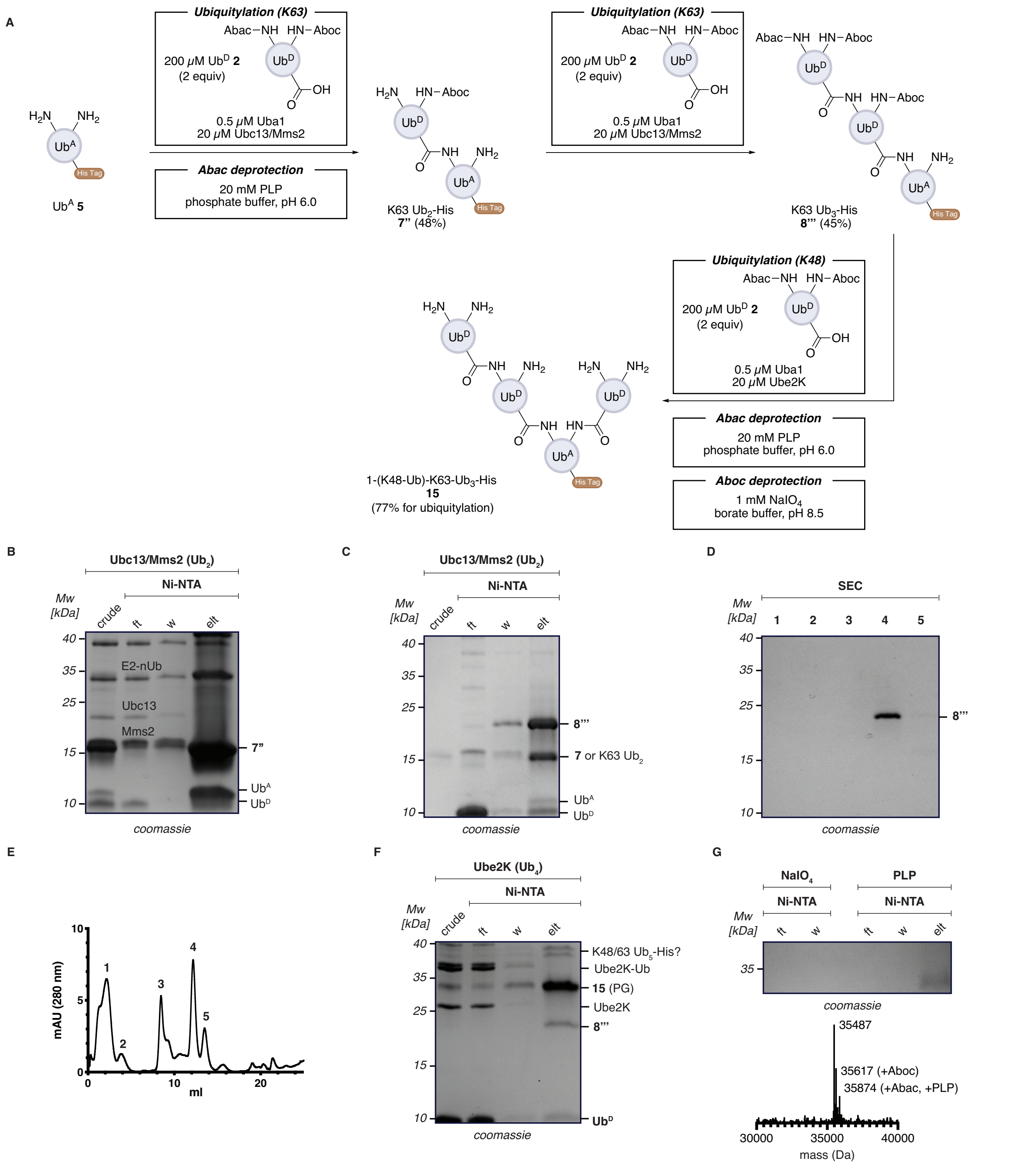

Figure S8. Branched Ub chain construction cycle. (A) Graphical scheme of Ub chain synthesis. Conversions were calculated based on densitometry of the SDS-gel after Coomassie staining. (B) SDS-PAGE gel of dimer synthesis after Coomassie staining (ft: flow through, w: wash, elt: elution). (C) SDS-PAGE gel of trimer synthesis after Coomassie staining (ft: flow through, w: wash, elt: elution). (D) SDS-PAGE gel of fractions after SEC purification (Coomassie staining). (E) Chromatogram of SEC. (F) SDS-PAGE gel of tetramer synthesis after Coomassie staining (ft: flow through, w: wash, elt: elution, **15** (PG): Ub_4 **15** before Aboc and Abac deprotection). (G) Top: SDS-PAGE gel of on-resin Aboc and Abac deprotection after Coomassie staining (ft: flow through, w: wash, elt: elution). The product was only observed in the elution fraction indicating there was no observable sample elution from the resin. Bottom: Deconvoluted mass of branched Ub_4 **15** after two deprotections. The

mass of incompletely deprotected compounds were also observed. Spectrum was obtained using LC-MS method B.

1.4 Full gels used in Figures

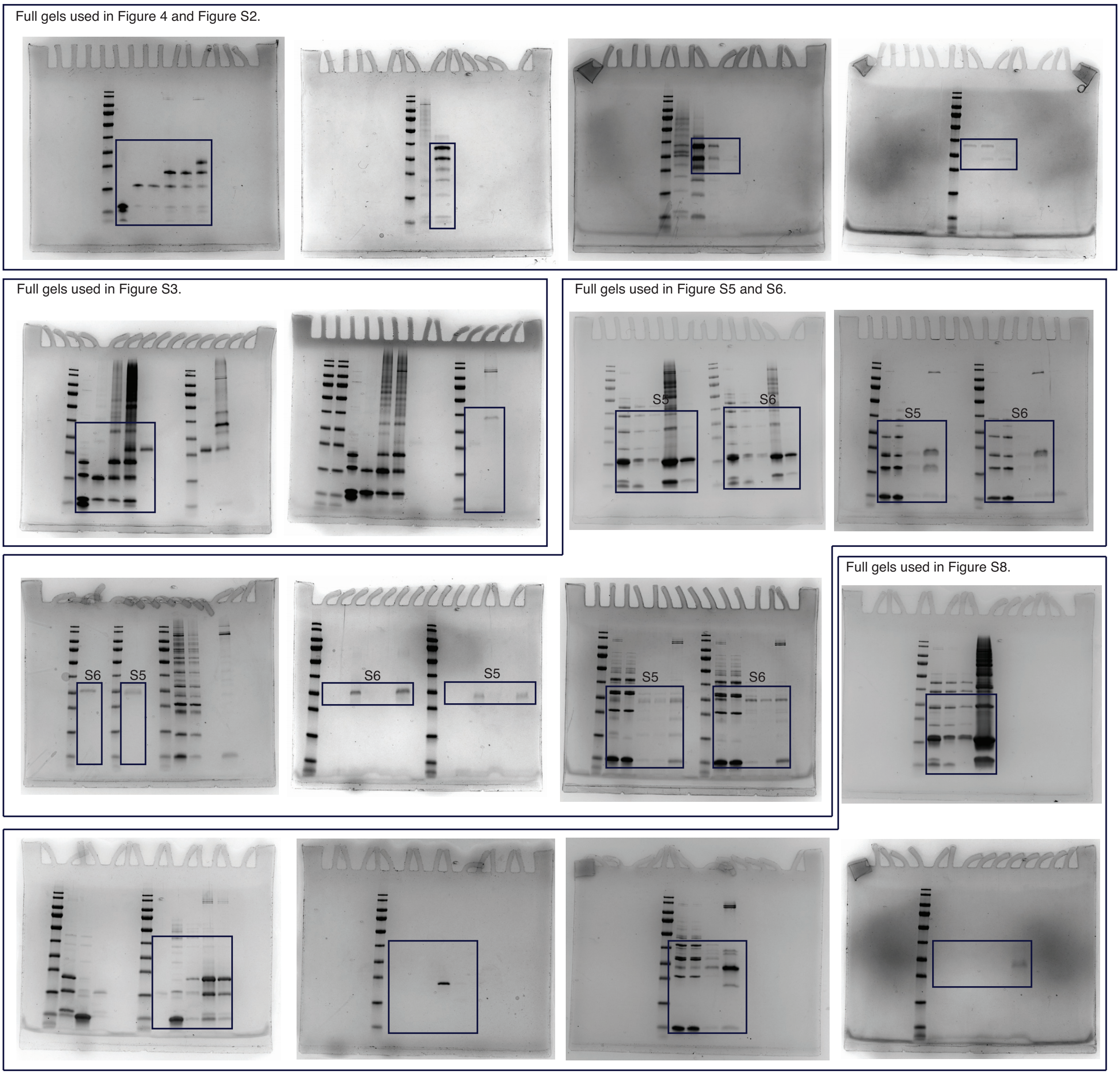

Figure S9. Full SDS-PAGE gels. Cropped sections highlighted were used in indicated Figures.

2 General Methods

2.1 Reagents

Fmoc-amino acids with suitable side-chain protecting groups, HCTU (*O*-(1*H*-6-chlorobenzotriazol-1-yl)-*N*,*N*,*N*',*N*'-tetramethyluroniumhexafluorophosphate) and HATU (1-[bis(dimethylamino)methylene]-1H-1,2,3-triazolo[4,5-b]pyridinium 3-oxide hexafluoro-phosphate) were purchased from Peptides International (Louisville, KY, USA) and ChemImpex (Wood Dale, IL, USA). HPLC grade CH_3CN from Sigma-Aldrich was used for analytical and preparative HPLC purification. DMF (> 99.8%) from Thommen-Furler AG was directly used without further purification for solid phase peptide synthesis. Other commercially available reagents and solvents were purchased from Sigma-Aldrich (Buchs, Switzerland), Acros Organics (Geel, Belgium) and TCI Europe (Zwijndrecht, Belgium) without further purification. H_2O used for reactions or assays was obtained from a Millipore purification system (MQ-H_2O). Common solvents for flash column chromatography were of technical grade and distilled prior to use. Anhydrous solvents for chemical reactions were purchased from Sigma Aldrich.

Thin layer chromatography (TLC) for reaction monitoring was conducted on glass-backed plates pre-coated with silica gel (Merck, Silica Gel 60 F254) and visualized by UV-quenching or staining with a $KMnO_4$-solution or ninhydrin solution. Flash column chromatography was performed on Sigma Aldrich SiO_2 Type F60 (high-purity grade, 60 Å pore size, 230-400 mesh particle size) using a forced flow of air (0.5-1.0 bar).

2.2 NMR

NMR spectra were recorded on Bruker Avance 500 MHz and Bruker Avance 400 MHz. Chemical shifts are reported in parts per million (ppm) and peaks were referenced to residual protonated solvents: $CDCl_3$ (d = 7.26 ppm, 77.2 ppm); DMSO-d_6 (d = 2.05, 39.5 ppm); MeOH-d_4 (d = 3.31 ppm, 49.0 ppm). NMR data is reported as follows: chemical shift, multiplicity (br, broad; s, singlet; d, doublet; t, triplet; q, quartet; p, pentet; m, multiplet), coupling constants in Hertz (Hz), integration.

2.3 Fmoc Solid phase peptide synthesis (Fmoc-SPPS)

Peptides were synthesised on a SYMPHONY® X (GYROS PROTEIN Technology) using Fmoc-SPPS chemistry. The following Fmoc-amino acids with side-chain protection groups were used: Fmoc-Ala-OH, Fmoc-Arg(Pbf)-OH, Fmoc-Asn(Trt)-OH, Fmoc-Asp(OtBu)-OH,, Fmoc-Gln(Trt)-OH, Fmoc-Glu(OtBu)-OH, Fmoc-Gly-OH, Fmoc-His(Trt)-OH, Fmoc-Ile-OH, Fmoc-Leu-OH, Fmoc-Lys(Boc)-OH, Fmoc-Met-OH, Fmoc-Nle-OH, Fmoc-Phe-OH, Fmoc-Pro-OH, Fmoc-Ser(tBu)-OH, Fmoc-Thr(tBu)-OH, Fmoc-Trp(Boc)-OH, Fmoc-Tyr(tBu)-OH, Fmoc-Val-OH. SPPS was performed on Rink-amide polystyrene resin or 2-chlorotrityl polystyrene resin. Manual loading of the first amino acid residue on the resin and subsequent Fmoc-SPPS, followed established standard protocols.[11] A brief summary of the utilised synthesis protocols: Fmoc-deprotections were performed with 20% piperidine in DMF

(2 x 8 min). Couplings were performed with Fmoc-amino acid (4.0 equiv relative to resin substitution), HCTU (3.9 equiv) and NMM (8.0 equiv) in DMF for 45 min. For cycles from P38 to M1, the coupling step was repeated once more (double coupling) before Fmoc-deprotection and coupling. After coupling, unreacted free amine was capped by treatment with 20% acetic anhydride in DMF and 2 M *i*Pr_2NEt in NMP (2 x 5 min).

2.4 Coupling of special amino acids

MQIFV**KT**LTG^{10} KT**IT**LEVEPS^{20} **DT**IENVKAKI^{30} QDKEGIPPDQ^{40} QRLIF**AG*K***QL^{50} E**DG**RTLSDYN^{60} IQ***K***E**ST**LHLV^{70} LRLRGG

- Coupling of pseudo proline dipeptides (KT, IT, DT and ST) and DMB-protected dipeptide (AG and DG). Highlighted in bold.

Pseudoproline dipeptides and DMB-protected dipeptides were coupled manually. The monomer (2.0 equiv) was dissolved in a minimal amount of DMF (minimum concentration of monomer: 0.2 M), HATU (2.0 equiv) and NMM (4.0 equiv) were added. After a brief pre-activation (30 s), the solution was added to the resin and allowed to react for 2 h.

- Longer coupling time was applied to T12 and P37 (2 h, double coupling). Highlighted with underbar.
- K48 and K63 were mutated to protected Lys when required. Highlighted in bold with italic.

The Aboc/Abac protected Fmoc-Lys (2.0 equiv) was dissolved in a minimal amount of DMF (minimum concentration of monomer: 0.2 M), HATU (2.0 equiv) and NMM (4.0 equiv) were added. After a brief pre-activation (30 s), the solution was added to the resin and allowed to react for 2 h.

- Met1Nle and N-terminal acetylation were applied for all the Ub monomers.

2.5 Resin cleavage procedures

Cleavage protocol for peptide segments synthesised on Rink Amide polystyrene resin or 2-chlorotritylchloride polystyrene resin: The dry resin was placed in a glass vial, a cleavage cocktail (20 mL/g resin, composition below) was added and the suspension shaken for 2 h. The resin was removed by filtration and washed with TFA (5 mL/g resin), the filtrate was placed in a plastic centrifugal tube (40 mL) and volatiles removed under reduced pressure. The residue was triturated with Et_2O (ca. 30 mL/g resin), centrifuged (3500 g, 3 min) and the supernatant was removed by decantation. This trituration/washing step was repeated twice. The crude material was dried using N_2 flow and dissolved in a suitable solvent (1:1 CH_3CN:H_2O + 0.1% TFA) for RP-HPLC purification.

Cleavage cocktail composition used:

Condition A: 90.5:5:2.5:2 TFA:H_2O:Phenol:TIPS

For 2-chlorotritylchloride polystyrene resin

Condition B: 95:2.5:2.5 TFA:DODT:H_2O

For α-ketoacid segments synthesised on α-ketoacid-bound Rink-amide resins[11]

2.6 KAHA ligation[11]

α-Ketoacid Segment 1 (1.0 equiv) and Opr Segment 2 (6.0 equiv) were dissolved in 9:1 DMSO/H_2O with 0.1 M oxalic acid (final α-ketoacid peptide concentration 10 mM). The mixture was heated to 60 °C for 18 h. After consumption of Segment 1, the reaction mixture was diluted 10-fold using 0.2 M Na_2CO_3/$NaHCO_3$ solution to a final concentration of 1 mM. The resulting solution was agitated at rt for 2 h. The O to N rearrangement was monitored by analytical HPLC. Upon completion, the reaction mixture was acidified using CH_3CN/H_2O (1:1) containing 0.1% TFA, and purified by RP-HPLC.

2.7 Folding of ubiquitin variants

The lyophilized powder of Ub^D **4** was dissolved in DMSO, then quickly diluted to 50 mM NaOAc pH 4.5. Then the protein was purified cation exchange chromatography (Mono S 5/50 GL) with buffer A (50 mM NaOAc pH 4.5) and a gradient of buffer B (buffer A with 1 M NaCl). The buffer was exchanged using desalting column (HiTrap™ Desalting 5 ml, performed on an ÄKTA pure chromatography system at 4 °C) with 50 mM HEPES pH 7.5, 150 mM NaCl. The other lyophilised KAHA ligation products or SPPS products were dissolved in 6 M Gdn HCl, 100 mM HEPES (pH 7.5), then dialyzed against 50 mM HEPES 150 mM NaCl (pH 7.5) using Slide-A-Lyzer® MINI Dialysis Devices (3.5K MWCO, 2 mL, Thermo Scientific) or SnakeSkin™ dialysis tubing (3.5 K MWCO, Thermo Scientific). Any precipitate formed during dialysis was removed by filtration, and the resulting filtrates were concentrated using VivaSpin 500 centrifugal concentrators 3000 MWCO from Sartorius Stedim Lab (Stonehouse, UK) or Amicon® Ultra-15 3 k from Merck at 4 °C.

2.8 High-Performance Liquid Chromatography

Peptides and protein segments were analysed and purified by reverse phase high performance liquid chromatography (RP-HPLC) on Jasco analytical and preparative instruments equipped with dual pumps, mixer and in-line degasser, a variable wavelength UV detector (simultaneous monitoring of the eluent at 220 nm, 254 nm and 301 nm) and a Rheodyne injector fitted with a 20 µL, 500 µL, 5 mL or 20 mL injection loop or on a Gilson preparative instrument fitted with a 20 mL injection loop. The columns were preheated using an Alltech column heater or a water bath (preparative HPLC). The mobile phase for RP-HPLC were Milipore-H_2O containing 0.1% TFA and HPLC-grade CH_3CN containing 0.1% TFA. In the HPLC analysis and purifications, TFA was always used as solvent modifier. Analytical HPLC was performed on a Shiseido Capcell Pak UG80 C18 UG120 (5 µm, 120

Å pore size, 4.6 mm I.D. x 250 mm) column, on a Shiseido Capcell Pak UG80 C18 UG 80 (5 µm, 120 Å pore size, 4.6 mm I.D. x 250 mm) column or on a Shiseido MGII C18 column (5 µm, 4.6 mm I.D. x 250 mm) at a flow rate of 1 mL/min. Semi-preparative HPLC was performed on Proteonavi C4 column (10 x 250 mm) with a flow rate of 5 mL/min.

Preparative HPLC was performed on a Shiseido Capcell Pak MGII column (5 µm, 100 Å pore size, 20 mm I.D. x 250 mm), Proteonavi C4 column (20 x 250 mm), Shiseido Capcell Pak UG80 C18 column (5 µm, 80 Å pore size, 50 x 250 mm), or Shiseido Capcell Pak C18 column (50 x 250 mm) at the indicated flow rates (typically 10 or 40 mL/min).

The column was pre-equilibrated at the starting solvent composition for 3–8 min. After injection of the sample, the solvent composition was run to the final solvent composition (e.g. 70% CH_3CN). After the gradient run time, the solvent composition was changed to 95% CH_3CN over 1 min and the column was flushed for 8–10 min. Over 1 min, the solvent composition was changed to the starting % of CH_3CN and the run ended for 8–10 min of re-equilibration. For the sake of simplicity, only the gradient time and the starting and end composition of the eluent will be stated at the individual experiments, although all experiments included the full cycle as described above.

2.9 LC-MS measurement

Method A: Waters-Xevo-G2-X5-QTOF

LC-MS measurement was performed on a ACQUITY UPLC® Protein BEH C4 column (300 å, 1.7 µm, 2.1 mm x 100 mm, 1/pk, Waters™) with a flow rate of 0.4 mL/min and 5 to 85% CH_3CN concentration over 8.5 min. The mobile phases for LC were Milipore-H_2O containing 0.1% formic acid and HPLC-grade CH_3CN containing 0.1% formic acid.

Ionisation method: ESI

Mass Analyzer: Quadruple-Time of Flight (Qq-TOF)

Method B: Bruker-Compact

LC-MS measurement was performed on a ACQUITY UPLC® Protein BEH C4 column (300 å, 1.7 µm, 2.1 mm x 100 mm, 1/pk, Waters™) with a flow rate of 0.4 mL/min and 10 to 95% CH_3CN concentration over 27 min. The mobile phases for LC were Milipore-H_2O containing 0.1% formic acid and HPLC-grade CH_3CN containing 0.1% formic acid.

Ionisation method: ESI

Mass Analyzer: Quadruple-Time of Flight (Qq-TOF)

2.10 High-resolution Mass Spectroscopy

High-resolution mass spectra were obtained by The Molecular and Biomolecular Analysis Service (MoBiAS) in the Department of Chemistry and Applied Bioscience at ETH Zürich on a Bruker solariX -

ESI-FTICR-MS, a Bruker maXis - ESI-Qq-TOF-MS, a Bruker solariX – MALDI-FTICR-MS, Bruker-Compact-20260, or a Bruker UltraFlex II – MALDI-TOF-MS.

2.11 Reagents for protein expression

Chemical reagents were purchased from Sigma Aldrich (Buchs, Switerland), Fluorochem (Glossop, UK), Novagentek (Ankara, Turkey) and used without further purification. Restriction enzymes, Phusion and Q5 High-Fidelity DNA polymerases, and PCR reagents were purchased from New England BioLabs (Ipswich, MA, USA). DNase I was obtained from Roche Diagnostics GmbH (Mannheim, Germany). Lysozyme (22500 U/mg) was obtained from Axon Lab AG (Baden, Switzerland).

pET3a-hUBA1 was a gift from Titia Sixma (Addgene plasmid #63571) and was used to express Uba1-His.[12] pET3d-E2-25K-C170S was a gift from Cecile Pickart (Addgene plasmid #18892) and was cloned into pGEX6p-1 vector having N-terminal 3C protease-cleavable GST tag. It was used to express Ube2K. pGEX-Ubc13 was a gift from Cecile Pickart (Addgene plasmid #18894) and was cloned into pGEX6p-1 vector having N-terminal 3C protease-cleavable GST tag. It was used to express Ubc13. pET16b-Mms2 was a gift from Cecile Pickart (Addgene plasmid #18893) and was cloned into pGEX6p-1 vector having N-terminal 3C protease-cleavable GST tag. It was used to express Mms2. pET42-3C protease was a gift from David Waugh (Addgene plasmid #78571) and was cloned into pGEX4T-2 vector having N-terminal thrombin cleavable GST tag. It was used to express GST-3C protease. DNA purification kits were purchased from Fisher Scientific (Geel, Belgium) and Zymo Research (Irvine, CA, USA). Gibson assembly master mix was prepared as reported.[13] Ampicillin sodium salt was obtained from AppliChem GmbH (Darmstadt, Germany), chloramphenicol form Hänseler AG (Herisau, Switzerland). Ni-NTA agarose resin was obtained from Qiagen GmbH (Hilden, Germany). Glutathione Sepharose 4 Fast Flow was obtained from Cytiva (Massachusetts, USA). Dialysis tubing and devices (SnakeSkin™ dialysis tubing, or Slide-A-Lyzer™ Mini dialysis devices for samples smaller than 2 mL, both with 3.5 kDa MWCO) were obtained from Thermo Fisher Scientific (Waltham, MA, USA). Amicon Ultra centrifugal filters were purchased from Merck (Darmstadt, Germany), VivaSpin 500 centrifugal concentrators from Sartorius Stedim Lab (Stonehouse, UK). All buffers were prepared using Milli-Q water, pH adjusted for the temperature at which the buffer was used, and sterile-filtered (0.2 µm membrane filter). Oligonucleotide synthesis and sequencing was carried out by Microsynth AG (Balgach, Switzerland).

2.12 Gel electrophoresis

Sodium dodecyl sulfate-polyacrylamide gel electrophoresis (SDS-PAGE) was carried out on a MiniPROTEAN Tetra Cell system (Bio-Rad) connected to a PowerPac Basic (Bio-Rad) programmable power supply. Reducing samples were treated with an equal volume of sample buffer (Laemmli 2x Concentrate, Sigma Aldrich), incubated at rt for 10 min and stored at –20 °C until

separation. A 10-180 kDa pre-stained protein ladder (Thermo Fisher) was applied to at least one well of each gel (2 µL). Samples were separated on 8-16% gradient Mini-PROTEAN TGX Precast gels (Bio-Rad) for 28 min at 200 V. Gels were imaged on a Bio-Rad ChemiDoc MP Imaging System (Coomassie staining) using the Image Lab Touch Software (Version 2.4.0.03). Images were cropped for illustration purposes.

2.13 Gelcode blue staining

Samples were resolved using SDS-PAGE on a 8-16% polyacrylamide gel (Bio-Rad). The membrane was washed with ddH_2O for at least 2 times and was added GelCode™ Blue Stain Reagent (Thermo Scientific, Catalog number: 24590) to completely cover the gel. The membrane was microwaved until the solution began to boil and incubated for at least 30 minutes until reaching to a proper stain intensity. The gel was washed with ddH_2O for 3 times and de-stained in ddH_2O for at least 3 h until a good band/background ratio was obtained. Gels were imaged on a Bio-Rad ChemiDoc MP Imaging System using the Image Lab Touch Software (Version 2.4.0.03). Images were cropped for illustration purposes.

2.14 Fast Protein Liquid Chromatography (FPLC)

Protein chromatography was performed on an ÄKTA pure chromatography system (GE Healthcare) at 4 °C. Anion exchange chromatography was performed using the strong ion exchange columns Mono Q 5/50 GL, cation exchange chromatography was performed using the strong ion exchange columns Mono S 5/50 GL, and size exclusion chromatography was performed using HiLoad 16/600 Superdex 200 pg column or Superdex 75 3.2/300 column. All columns were purchased from GE Healthcare. Protein elution was monitored at 280 nm. FPLC data was acquired using UNICORN (Version 6.3.2.89).

2.15 Protein quantification and UV-Vis spectroscopy

Protein concentration and OD600 measurements were carried out on a NanoDrop 2000c UV-Vis spectrophotometer. Protein concentrations were determined by the absorption at 280 nm using extinction coefficients calculated by ProtParam55 based on the amino acid sequence.

3 Synthesis of Abac and Aboc protected Fmoc-Lys

3.1 Synthesis of homoserine lactone **S2**

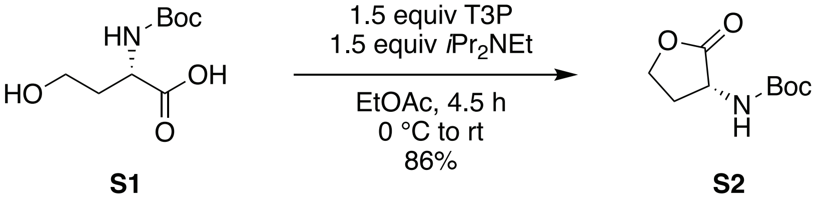

S1 **S2**

In a flask, (*S*)-Boc-homoserine (11 g, 35 mmol, 1.0 equiv) was dissolved in 330 mL of EtOAc and the mixture was added *i*Pr_2NEt (53 mmol, 1.5 equiv) and propylphosphonic anhydride (T3P, 42 mmol, 1.2 equiv) at 0 °C. After stirring for 4.5 h at rt, the mixture was diluted with EtOAc (350 mL) and washed with H_2O, sat aq $NaHCO_3$, sat aq NH_4Cl and brine (each 200 mL). The organic layer was dried over Na_2SO_4, filtered and concentrated under vacuum to obtain **S2** (6.1 g, 86%) as a white solid. The product was used for the next step without further purification.

^{1}H NMR (500 MHz, $CDCl_3$) δ 5.07 (s, 1H), 4.44 (td, *J* = 9.1, 1.2 Hz, 1H), 4.35 (s, 1H), 4.24 (ddd, *J* = 11.4, 9.3, 5.9 Hz, 1H), 2.76 (q, *J* = 8.2, 5.7 Hz, 1H), 2.25 – 2.12 (m, 1H), 1.45 (s, 9H).

^{13}C NMR (126 MHz, $CDCl_3$) δ 175.28, 156.62, 80.60, 65.75, 50.19, 30.66, 28.25.

HRMS (ESI): calculated for $[M+Na]^+$: 224.0893, found: 224.0892.

The data obtained were identical to the previously reported compound.[14]

3.2 Synthesis of Boc Hse NHMe **S3**

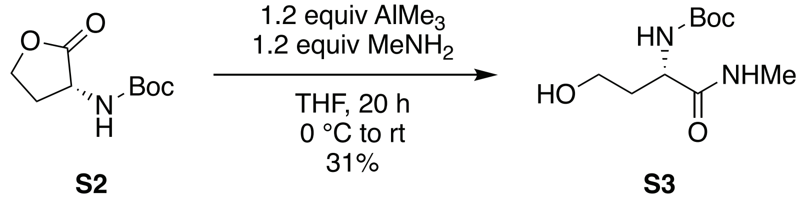

S2 **S3**

In a 200 mL 2-neck round bottom flask, methylamine (2 M in THF solution, 6.6 mL, 13.2 mmol, 1.2 equiv) was dissolved in THF (48 mL) under N_2 atmosphere. To the solution, trimethylaluminum (2 M in toluene solution, 6.6 mL, 13.2 mmol, 1.2 equiv) was added dropwise at 0 °C. After stirring for 1 h at rt, the reaction mixture was added **S2** (2.2 g, 11 mmol, 1.0 equiv) in THF (48 mL) dropwise at rt. The reaction mixture was allowed to stir for 20 h at rt and quenched by the addition of 10% citric acid solution (100 mL). The mixture was extracted with EtOAc (200 mL, 3 times) and the combined organic layers were concentrated under vacuum. The residue was purified by flash column chromatography (CH_2Cl_2:MeOH = 8:1) to obtain **S3** as a white crystalline solid (0.78 g, 31%).

^{1}H NMR (500 MHz, $CDCl_3$) δ 6.81 (s, 1H), 5.67 (d, *J* = 6.9 Hz, 1H), 4.32 (q, *J* = 8.2, 7.3 Hz, 1H), 3.70 (dq, *J* = 12.7, 6.7, 5.7 Hz, 3H), 2.81 (d, *J* = 4.8 Hz, 3H), 1.98 (ddd, *J* = 14.6, 7.6, 5.0 Hz, 1H), 1.75 (d, *J* = 11.2 Hz, 1H), 1.43 (s, 9H).

^{13}C NMR (126 MHz, $CDCl_3$) δ 172.61, 156.59, 80.30, 58.53, 51.37, 36.41, 28.25, 26.26.

HRMS (ESI): calculated for $[M+Na]^+$: 255.1315, found: 255.1313.

IR (ATR): ν^{max} (cm^{-1}) = 3469 (w), 3332 (m), 2991 (w), 2969 (w), 2887 (w), 1676 (m), 1636 (m), 1547 (m), 1519 (s)

3.3 Synthesis of Abac-PNP carbonate **S4**

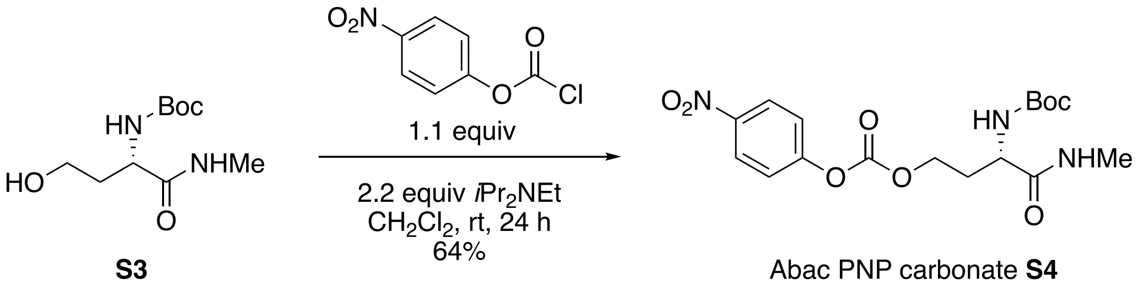

In a 100 mL round bottom flask, **S3** (0.78 g, 3.4 mmol, 1.0 equiv) was dissolved in 33 mL of CH_2Cl_2 and the mixture was added *i*Pr_2NEt (2.2 equiv) and 4-nitrophenyl chloroformate (PNPCl, 1.1 equiv). After stirring for 24 h at rt, the mixture was directly concentrated under vacuum and purified by flash column chromatography (EtOAc:hexanes = 2:1) to obtain **S4** as a white foam (0.85 g, 64%).

^1H NMR (500 MHz, $CDCl_3$) δ 8.30 – 8.23 (m, 2H), 7.41 – 7.35 (m, 2H), 6.45 – 6.41 (m, 1H), 5.30 (d, *J* = 8.6 Hz, 1H), 4.44 – 4.28 (m, 3H), 2.82 (d, *J* = 4.8 Hz, 3H), 2.33 – 2.24 (m, 1H), 2.06 (ddt, *J* = 14.6, 8.1, 5.7 Hz, 1H), 1.43 (s, 9H).

^{13}C NMR (126 MHz, $CDCl_3$) δ 171.72, 155.70, 155.44, 152.35, 145.39, 126.11, 125.27, 121.79, 115.61, 80.48, 65.78, 51.32, 31.58, 28.26, 26.32.

HRMS (ESI): calculated for $[M+Na]^+$: 420.1377, found: 420.1380.

IR (ATR): ν^{max} (cm^{-1}) = 3679 (br), 3327 (m), 2971 (m), 2903 (w), 1757 (m), 1681 (m), 1651 (m), 1616 (m)

3.4 Synthesis of Abac protected Fmoc-Lys-OH **S5**

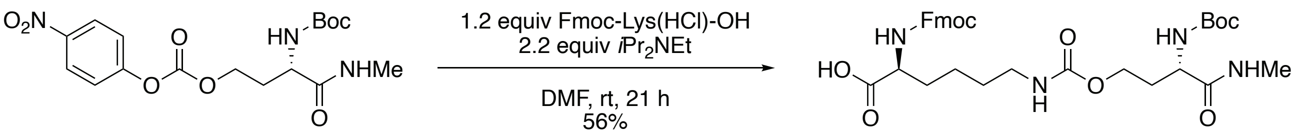

In a 50 mL round bottom flask, **S4** (0.85 g, 2.1 mmol, 1 equiv) was dissolved in 20 mL of DMF and the mixture was added *i*Pr_2NEt (2.2 equiv) and Fmoc-Lys-OH HCl (1.2 equiv). After stirring for 21 h at rt, the mixture was diluted with 50 mL of EtOAc and washed with 100 mL of water and 1 M aqueous HCl solution. The aqueous layer was extracted with 100 mL of EtOAc twice, and the combined organic extracts were concentrated under vacuum and purified by flash column chromatography (CH_2Cl_2:MeOH = 10:1 with 0.1% AcOH) to obtain **S5** as a white solid (0.75 g, 56%).

^{1}H NMR (500 MHz, DMSO) δ 7.90 (dd, J = 7.5, 1.0 Hz, 2H), 7.76 – 7.71 (m, 3H), 7.62 (d, J = 8.0 Hz, 1H), 7.43 (td, J = 7.5, 1.2 Hz, 2H), 7.34 (td, J = 7.4, 1.2 Hz, 2H), 7.09 (d, J = 5.7 Hz, 1H), 6.95 (d, J = 8.1 Hz, 1H), 4.29 (d, J = 8.0 Hz, 2H), 4.25 – 4.20 (m, 1H), 3.99 – 3.84 (m, 4H), 3.01 – 2.91 (m, 2H), 2.58 (d, J = 4.6 Hz, 3H), 1.90 (dq, J = 13.1, 7.3 Hz, 1H), 1.78 – 1.65 (m, 2H), 1.60 (qd, J = 9.4, 5.1 Hz, 1H), 1.38 (s, 13H).

^{13}C NMR (126 MHz, DMSO) δ 174.44, 172.44, 156.63, 156.59, 155.75, 144.32, 144.28, 141.20, 141.18, 128.11, 127.54, 125.78, 125.75, 120.59, 120.58, 78.58, 66.06, 61.13, 54.25, 51.90, 47.13, 40.49, 40.42, 40.32, 40.25, 40.16, 39.99, 39.91, 39.82, 39.66, 39.49, 31.86, 30.88, 29.45, 28.65, 26.11, 23.41.

HRMS (ESI): calculated for $[M+H]^+$:627.3025, found:627.3019.

IR (ATR): ν^{max} (cm^{-1}) = 3324 (m), 2944 (br), 2862 (w), 1737 (m), 1686 (s), 1656 (m), 1623 (m), 1522 (s)

3.5 Synthesis of diol **S6**

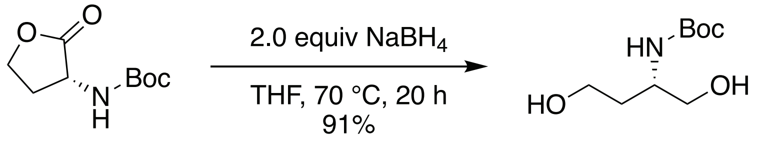

S2 **S6**

In a flask, **S2** (1.4 g, 7.0 mmol, 1.0 equiv) was dissolved in 15 mL of THF. The mixture was added $NaBH_4$ (2.0 equiv) and warmed up to 70 °C. After stirring for 20 h at 70 °C, the reaction mixture was cooled down to rt and quenched while stirring by slowly adding 5 ml of MeOH followed by 15 mL of sat. aq. $NaHCO_3$. 30 mL of EtOAc was added to the mixture and the phases were separated. The aqueous layer was extracted with EtOAc (30 mL x 2). The combined organic layers were concentrated under vacuum. The remaining clear colorless oil was employed to the next reaction without further purification (1.4 g, 91%).

^{1}H NMR (500 MHz, $CDCl_3$) δ 5.15 – 5.10 (m, 1H), 3.83 (s, 2H), 3.68 (dd, J = 26.0, 14.1 Hz, 3H), 1.79 (s, 1H), 1.68 – 1.53 (m, 1H), 1.43 (s, 9H).

^{13}C NMR (126 MHz, $CDCl_3$) δ 156.98, 79.88, 65.20, 58.69, 49.34, 34.77, 28.32.

HRMS (ESI): calculated for $[M+Na]^+$: 228.1206, found: 228.1204.

The data obtained were identical to the previously reported compounds.[15]

3.6 Synthesis of **S7**

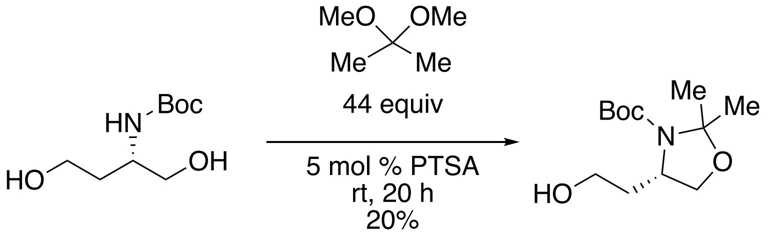

S6 **S7**

In a flask, **S6** (3.4 g, 16.8 mmol, 1.0 equiv) was dissolved in 91 mL of dimethoxypropane (DMP, 44 equiv). The mixture was added PTSA (0.05 equiv) and warmed up to 75 °C. After stirring for 20 h at 75 °C, the reaction mixture was cooled down to rt and added sodium bicarbonate and filtered. The crude was concentrated under vacuum and added CH_2Cl_2 (50 mL), silica-gel (60 g) and H_2O (4 mL). The mixture was stirred for 3 days at rt and filtered. The filtercake was washed with EtOAc. The combined organic layers were concentrated under vacuum. The remaining white crystalline solid was purified by flash column chromatography (hexanes:EtOAc = 1:1) to obtain **S7** (0.8 g, 20%).

^{1}H NMR (500 MHz, $CDCl_3$) δ 4.10 (tq, J = 10.0, 5.0 Hz, 1H), 3.91 (tt, J = 8.9, 5.1 Hz, 1H), 3.63 (q, J = 5.2 Hz, 1H), 3.55 (ddt, J = 12.4, 8.1, 4.0 Hz, 1H), 3.52 – 3.42 (m, 1H), 1.71 (dqd, J = 17.3, 8.3, 3.8 Hz, 2H), 1.46 (dt, J = 7.8, 3.1 Hz, 3H), 1.44 – 1.37 (m, 12H).

^{13}C NMR (126 MHz, $CDCl_3$) δ 153.57, 93.48, 80.73, 67.99, 58.68, 54.10, 37.37, 28.35, 27.66, 24.30.

HRMS (ESI): calculated for $[M+Na]^+$:268.1519, found:268.1518.

Rotameric mixture. The data obtained were identical to the previously reported compounds.[16]

3.7 Synthesis of Aboc-PNP carbonate **S8**

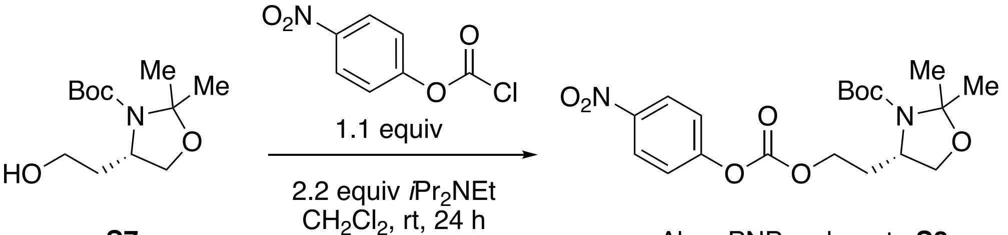

S7 Aboc PNP carbonate **S8**

In a flask, **S7** (0.5 g, 2.0 mmol, 1.0 equiv) was dissolved in 20 mL of CH_2Cl_2. The mixture was added *i*Pr_2NEt (2.2 equiv), 4-nitrophenyl chloroformate (1.1 equiv) and stirred at rt. After stirring for 24 h, the mixture was concentrated under vacuum and purified by flash column chromatography (hexanes:EtOAc = 3:1 with 0.5% NEt_3) to obtain **S8** (655 mg, 86%).

^{1}H NMR (500 MHz, $CDCl_3$) δ 8.30 – 8.25 (m, 2H), 7.41 – 7.36 (m, 2H), 4.37 (tt, *J* = 6.8, 3.9 Hz, 2H), 4.19 – 3.95 (m, 2H), 3.85 – 3.79 (m, 1H), 2.25 – 2.12 (m, 1H), 2.02 (ddt, *J* = 14.1, 7.7, 6.4 Hz, 1H), 1.60 (d, *J* = 20.9 Hz, 3H), 1.53 – 1.50 (m, 3H), 1.50 (s, 9H).

^{13}C NMR (126 MHz, $CDCl_3$) δ 162.72, 155.55, 152.47, 145.37, 126.15, 125.29, 121.80, 115.58, 93.77, 80.33, 67.22, 66.93, 54.74, 33.01, 32.46, 28.42, 27.65, 26.88, 24.36, 23.05.

HRMS (ESI): calculated for $[M+H]^+$: 411.1762, found: 411.1764.

IR (ATR): ν^{max} (cm^{-1}) = 3672 (br), 2979 (m), 2900 (w), 1757 (m), 1697 (m), 1613 (w), 1590 (m)

3.8 Synthesis of Aboc protected Fmoc-Lys-OH **S9**

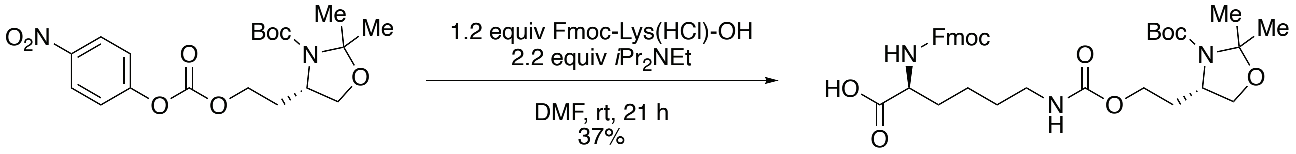

Aboc PNP carbonate **S8** Aboc Fmoc-Lys-OH **S9**

In a 25 mL round bottom flask, **S8** (0.58 g, 1.4 mmol, 1 equiv) was dissolved in 10 mL of DMF and the mixture was added *i*Pr_2NEt (3.6 equiv) and Fmoc-Lys-OH HCl (1.2 equiv). After stirring for 21 h at rt, the mixture was concentrated under vacuum and purified by flash column chromatography (hexanes:EtOAc = 1:4 with 0.05% acetic acid) to obtain **S9** as a white solid (0.34 g, 37%).

^1H NMR (500 MHz, $CDCl_3$) δ 7.78 (d, *J* = 7.5 Hz, 2H), 7.62 (d, *J* = 9.0 Hz, 2H), 7.41 (t, *J* = 7.5 Hz, 2H), 7.33 (t, *J* = 7.5 Hz, 2H), 5.70 (d, *J* = 38.0 Hz, 1H), 4.86 (d, *J* = 46.9 Hz, 1H), 4.61 – 4.35 (m, 3H), 4.26 – 4.11 (m, 3H), 4.00 (d, *J* = 49.3 Hz, 2H), 3.84 (d, *J* = 9.1 Hz, 1H), 3.21 (s, 2H), 2.13 (s, 1H), 1.87 (d, *J* = 33.4 Hz, 3H), 1.61 (s, 2H), 1.57 (s, 3H), 1.50 (d, *J* = 10.8 Hz, 14H).

^{13}C NMR (126 MHz, $CDCl_3$) δ 156.08, 152.53, 143.89, 143.73, 141.33, 127.73, 127.09, 125.11, 120.00, 93.52, 77.28, 77.23, 77.03, 76.78, 67.05, 55.20, 53.50, 47.18, 32.32, 31.47, 28.44, 28.35, 27.55, 26.78, 24.41.

HRMS (ESI): calculated for $[M+H]^+$:640.3229, found:640.3226.

IR (ATR): ν^{max} (cm^{-1}) = 3334 (br), 2974 (w), 2931 (br), 2870 (w), 1689 (s), 1575 (m), 1529 (m)

4 Synthesis of Aboc/Abac protected Ub monomers by Fmoc-SPPS

4.1 Ub donor (Ub^D) synthesis

Each Ub^D was synthesized on a 2-chlorotritylchloride resin preloaded with Fmoc-Gly-OH with a resin loading of 0.25 mmol/g. The synthesis was performed on a 0.49 mmol scale (2 g of resin) by automated Fmoc-SPPS up to Met1Nle using the procedure described in the General Methods. The resulting peptide was cleaved from the resin using condition A. Purification was performed by preparative HPLC (C-18, Reprosil-Pur 120 ODS-2, 5 μm, 50 x 250 mm) with a gradient of 15 to 75% over 30 minutes, with a flow rate of 40 mL/min. The pure product fractions were combined and lyophilised. Analytical HPLC and ESI-TOF confirmed the purity and exact mass of each Ub^D.

4.1.1 Ub^D **1** K48K/K63K-Abac free C-terminus

The pure product fractions were combined and lyophilised to obtain pure Ub^D **1** (610 mg, 70 µmol, 14% yield for peptide synthesis, resin cleavage and purification steps). Analytical HPLC and ESI-TOF confirmed the purity and exact mass.

HRMS (ESI): calculated for $C_{387}H_{643}N_{107}O_{122}$: 8747, found: 8746.

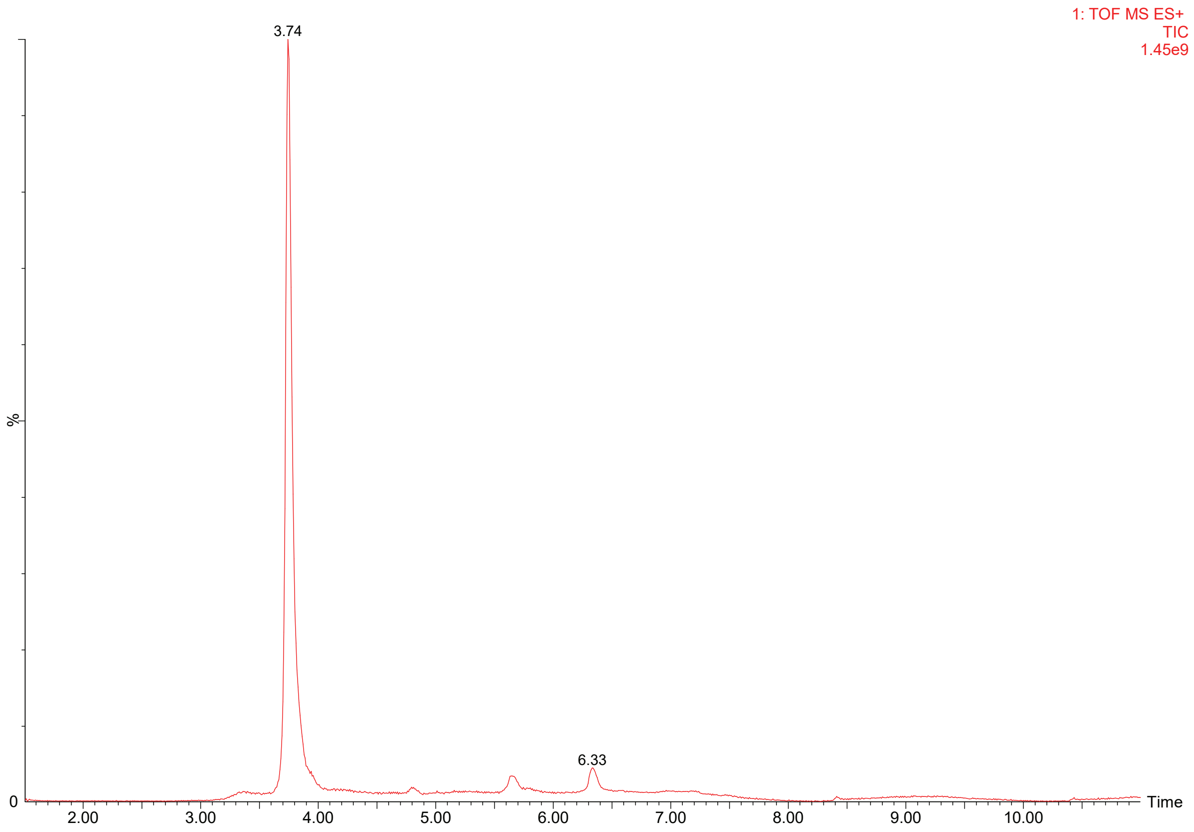

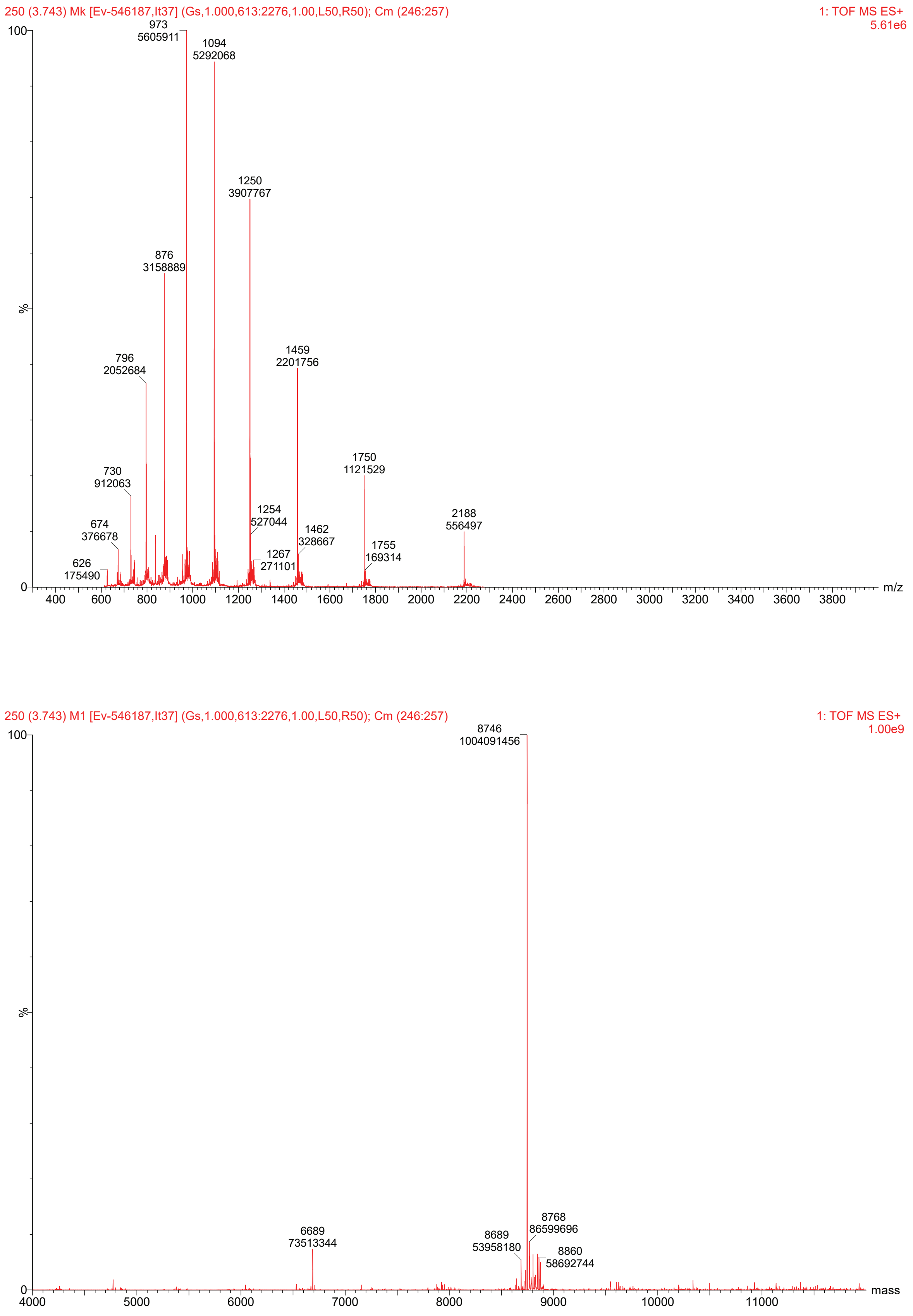

Figure S10. LC-MS analysis of Ub^{D} **1** K48K/K63K-Abac free C-terminus.

(top) Total ion chromatogram using LC-MS method A. (middle) Mass spectrum of peak at 3.7 min. (bottom) Deconvoluted mass spectrum of peak found in the middle spectrum.

4.1.2 Ub^{D} **2** K48K-Aboc/K63K-Abac free C-terminus

The pure product fractions were combined and lyophilised to obtain pure Ub^{D} **2** (510 mg, 58 µmol, 12% yield for peptide synthesis, resin cleavage and purification steps). Analytical HPLC and ESI-TOF confirmed the purity and exact mass.

HRMS (ESI): calculated for $C_{392}H_{652}N_{108}O_{125}$: 8878, found: 8878.

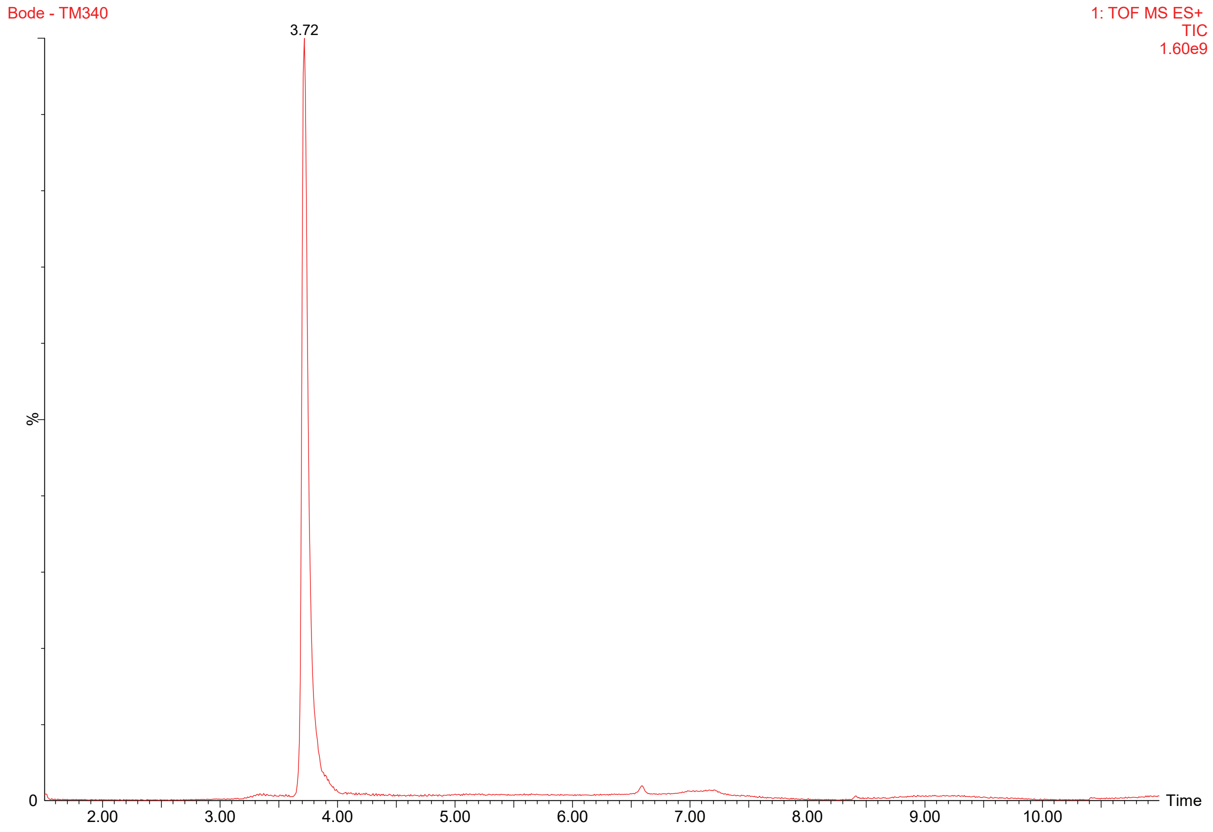

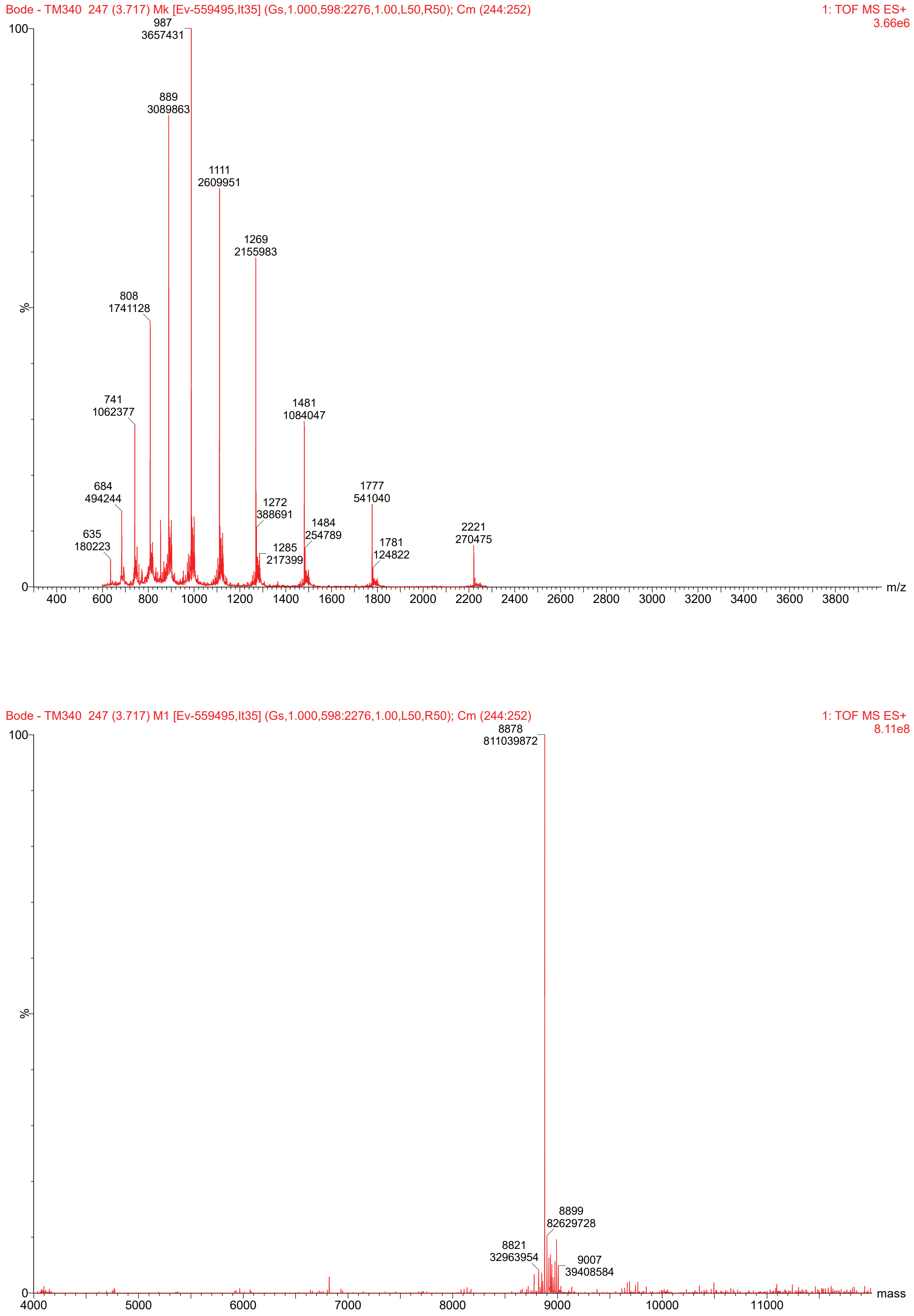

Figure S11. LC-MS analysis of Ub^{D} **2** K48K-Aboc/K63K-Abac free C-terminus.

(top) Total ion chromatogram using LC-MS method A. (middle) Mass spectrum of peak at 3.7 min. (bottom) Deconvoluted mass spectrum of peak found in the middle spectrum.

4.1.3 Ub^{D} **4** K48K-Abac/K63K-Aboc free C-terminus

The pure product fractions were combined and lyophilised to obtain pure Ub^{D} **4** (0.2% yield for peptide synthesis, resin cleavage, purification steps and folding). Analytical HPLC and ESI-TOF confirmed the purity and exact mass.

HRMS (ESI): calculated for $C_{392}H_{652}N_{108}O_{125}$: 8878, found: 8877.

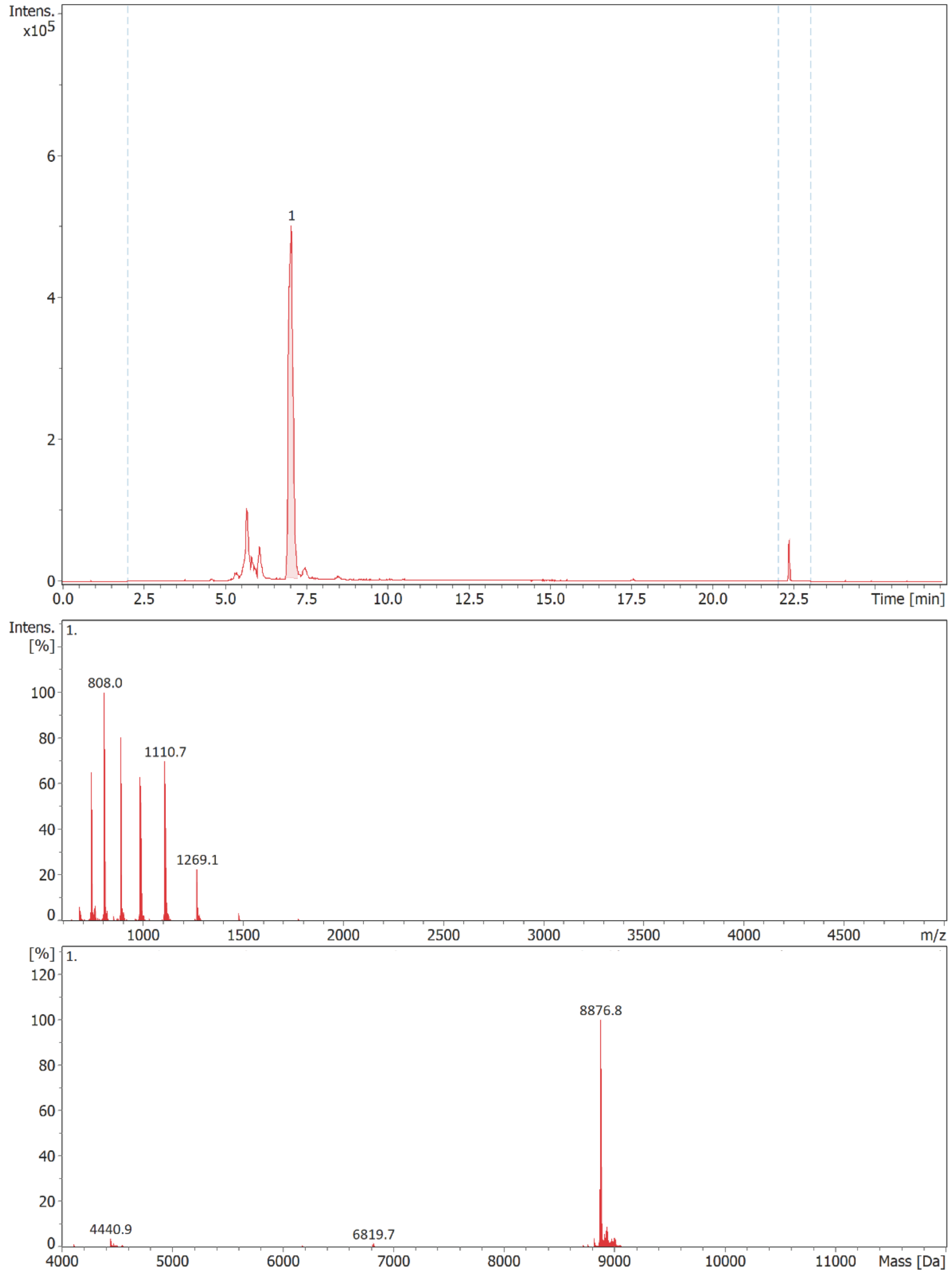

Figure S12. LC-MS analysis of Ub^D **4** K48K-Abac/K63K-Aboc free C-terminus.

(top) Total ion chromatogram using LC-MS method B. (middle) Mass spectrum of peak at 7.0 min. (bottom) Deconvoluted mass spectrum of peak found in the middle spectrum.

4.2 Ub^A **3** synthesis by KAHA ligation

Ub^A **3** was synthesized utilizing KAHA ligation.[1] Ub1-76-DF-α-ketoacid was synthesized on a Rink Amide resin preloaded with protected Fmoc-Phe-α-ketoacid with a resin loading of 0.28 mmol/g. The synthesis was performed on a 0.38 mmol scale by automated Fmoc-SPPS up to Met1Nle using the procedure described in the General Methods. The resulting peptide was cleaved from the resin using condition B. Purification of Ub1-76-DF-α-ketoacid was performed by preparative HPLC (C-18, Reprosil-Pur 120 ODS-2, 5 μm, 50 x 250 mm) with a gradient of 15 to 75% over 30 minutes, with a flow rate of 40 mL/min. The pure product fractions were combined and lyophilised to obtain 604 mg of slightly yellow powder (18%).

The obtained α-ketoacid segments (130 mg, 14 μmol, 1.0 equiv) and Opr-(His)$_6$-OH (80 mg, 85 μmol, 6.0 equiv) were dissolved in 9:1 DMSO/H_2O with 0.1 M oxalic acid (final α-ketoacid peptide concentration 10 mM). The mixture was heated to 60 °C for 20 h. After consumption of α-ketoacid segment, the reaction mixture was diluted 10-fold using 6.0 M Gdn HCl, 0.1 M Na_2CO_3/$NaHCO_3$ solution pH 9.5 to a final concentration of 1 mM. The resulting solution was agitated at rt for 3 h. The complete O to N rearrangement was monitored by analytical HPLC. Upon completion, the reaction mixture was acidified using CH_3CN/H_2O (1:1) containing 0.1% TFA, and purified by RP-HPLC. Purification was performed by preparative HPLC (C-18, Reprosil-Pur 120 ODS-2, 5 μm, 50 x 250 mm) with a gradient of 15 to 75% over 30 minutes, with a flow rate of 40 mL/min. The pure product fractions were combined and lyophilised to obtain white powder (50 mg, 5.0 μmol, 36% yield for KAHA ligation, O to N rearrangement and purification). Analytical HPLC and ESI-TOF confirmed the purity and exact mass.

HRMS (ESI): calculated for $C_{439}H_{705}N_{127}O_{134}$: 9906, found: 9907.

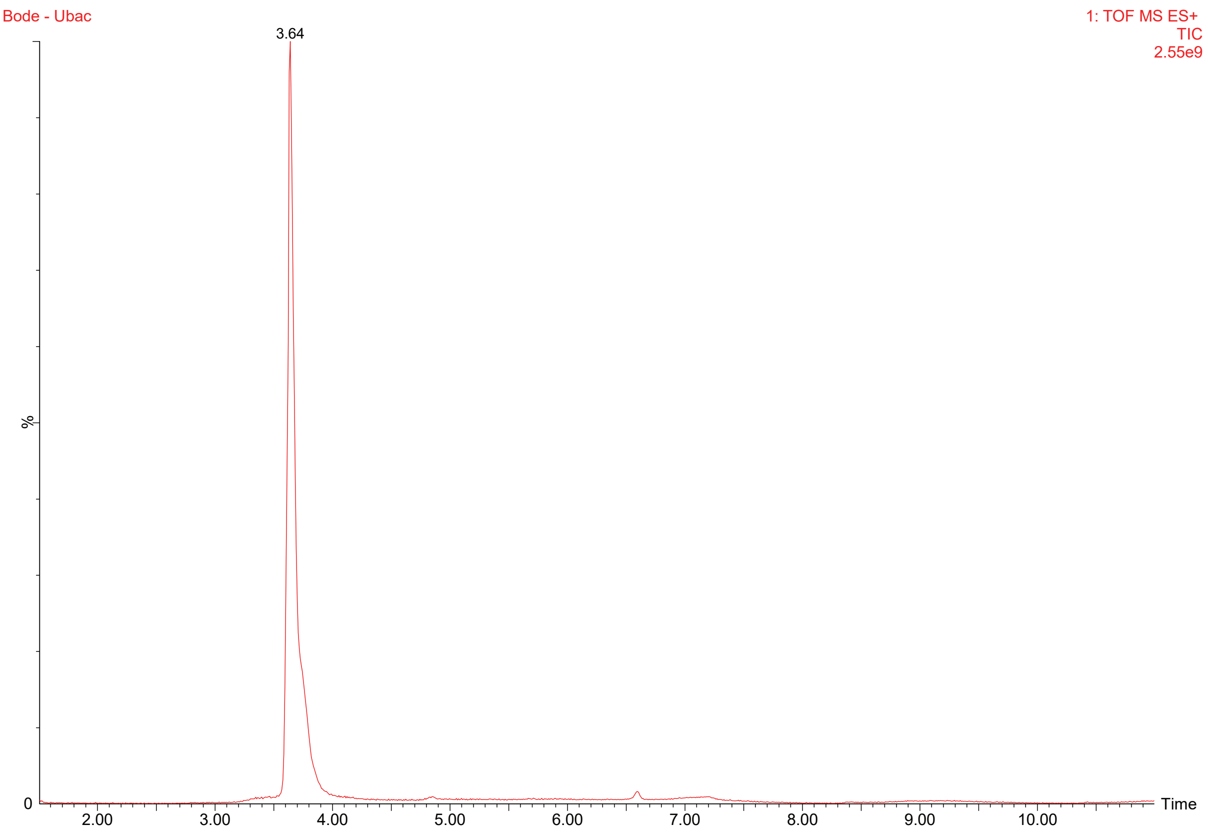
Bode - Ubac
1: TOF MS ES+
TIC
2.55e9
3.64
%
0
2.00
3.00
4.00
5.00
6.00
7.00
8.00
9.00
10.00
Time

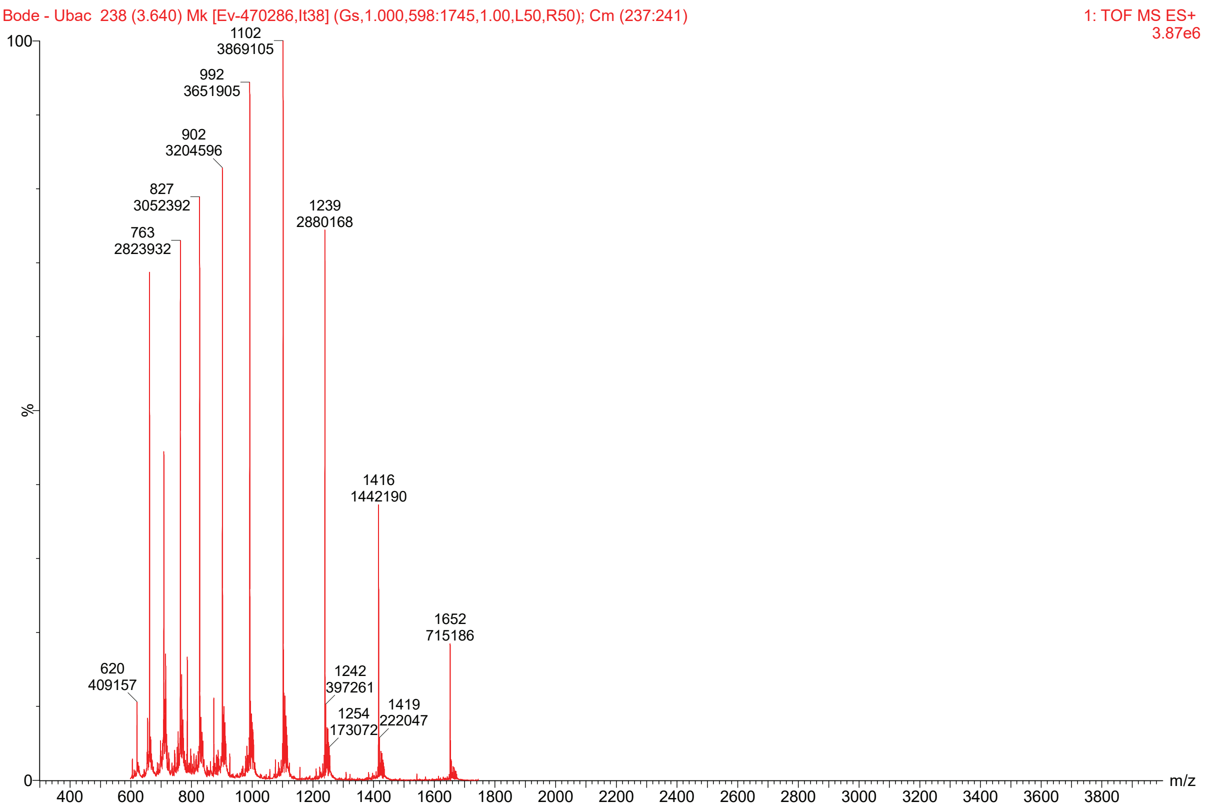
Bode - Ubac 238 (3.640) Mk [Ev-470286,It38] (Gs,1.000,598:1745,1.00,L50,R50); Cm (237:241)
1: TOF MS ES+
3.87e6
1102
3869105
992
3651905
902
3204596
827
3052392
763
2823932
1239
2880168
1416
1442190
1652
715186
620
409157
1242
397261
1254
173072
1419
222047
100
%
0
m/z

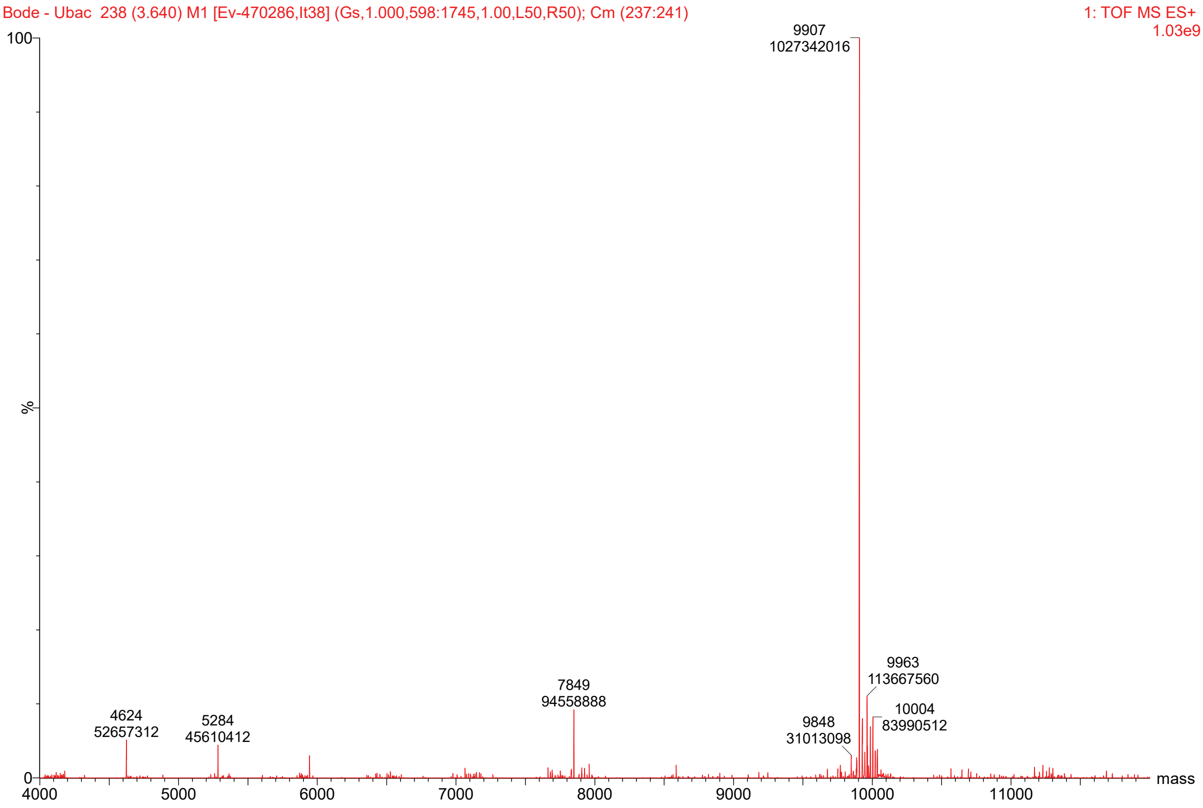

Figure S13. LC-MS analysis of Ub^A **3** K48K-Aboc/K63K His-tagged C-terminus. LC-MS Method A. (top) Total ion chromatogram using LC-MS method A. (middle) Mass spectrum of peak at 3.7 min. (bottom) Deconvoluted mass spectrum of peak found in middle spectrum.

5 Protein expression

5.1 Expression of Uba1

Chemically competent BL21 (DE3) cells for expression were heat-shock transformed with the plasmids. Single colonies were used to inoculate overnight precultures in selective lysogeny broth (LB) Miller medium. Following 1:100 dilution with fresh selective LB Miller medium, cultures were grown in baffled shake flasks at 37 °C until an OD600 of approximately 0.6 was reached. Protein expression was induced by addition of isopropyl β-D-1-thiogalactopyranoside at a final concentration of 0.25 mM. Expressions were carried out for overnight at 18 °C. Cells were collected by centrifugation (4,500 x g, 30 min, 4 °C), resuspended in 20 mL lysis buffer per L cell culture (50 mM Tris pH 8.0, 350 mM NaCl, 30 mM imidazole, 10% glycerol) and stored at –80 °C until purification. Cell suspensions were thawed on ice, supplemented with 1 mM PMSF, 1 mM DTT, lysozyme (20 µg/mL) and DNase I (0.1 mg/mL). Cells were lysed by sonication and the suspensions were cleared by centrifugation (16,000 x g, 20 min, 4 °C) and filtration (0.45 µm membrane filter). Supernatants were subjected to gravity Ni-NTA affinity purification using lysis buffer and elution buffer (lysis buffer containing 300 mM imidazole and 1 mM DTT), to obtain Uba1-His. Uba1-His was further polished by anion exchange chromatography (Mono Q 5/50 GL). Samples were dialyzed against 50 mM Tris pH 8.0, 50 mM NaCl and 1 mM DTT at 4 °C and purified with buffer A (20 mM Tris pH 8.0, 1 mM DTT) and a gradient of buffer B (buffer A with 1 M NaCl). Samples were concentrated and further purified by size exclusion chromatography (Hiload 16/600 superdex 200 pg) with 40 mM Tris pH 7.8, 50 mM NaCl, 1 mM TCEP. All samples were portioned into aliquots, flash-frozen in liquid N_2 and could be stored at –80 °C for months without noticeable loss of activity.

5.2 Expression of Ubc13/Mms2

Chemically competent BL21 CodonPlus(DE3) RIL for expression, were heat-shock transformed with the plasmids. Single colonies were used to inoculate overnight precultures in selective lysogeny broth (LB) Miller medium. The proteins were expressed in autoinduction medium.[17] Following 1:1000 dilution of overnight precultures were added and cells were grown 24 h at 30 °C. Cells were collected by centrifugation (4,500 x g, 30 min, 4 °C), resuspended in 20 mL lysis buffer per L cell culture (50 mM Tris pH 8.0, 350 mM NaCl, 30 mM imidazole, 10% glycerol) and stored at –80 °C until purification. Cell suspensions were thawed on ice, supplemented with 1 mM PMSF, 2.5 mM β-mercaptoethanol, lysozyme (20 µg/mL) and DNase I (0.1 mg/mL). Cells were lysed by sonication and the suspensions were cleared by centrifugation (16,000 x g, 20 min, 4 °C) and filtration (0.45 µm membrane filter). Supernatants were subjected to gravity GST affinity purification using lysis buffer. GST-Ubc13 and GST-Mms2 were cleaved on Glutathione Sepharose 4 Fast Flow by GST-3C protease. Then Ubc13 and Mms2 were incubated together for dimer complex and further polished by size exclusion chromatography (Hiload 16/600 superdex 200 pg) with 50 mM HEPES pH 7.5, 150 mM NaCl, 1 mM TCEP. Samples were portioned into aliquots, flash-frozen in liquid N_2 and could be stored at –80 °C for months without noticeable loss of activity.

5.3 Expresssion of Ube2K

Chemically competent BL21 CodonPlus(DE3) RIL for expression, were heat-shock transformed with the plasmids. Single colonies were used to inoculate overnight precultures in selective lysogeny broth (LB) Miller medium. The protein was expressed in autoinduction medium. Following 1:1000 dilution of overnight precultures were added and cells were grown 24 h at 30 °C. Cells were collected by centrifugation (4,500 x g, 30 min, 4 °C), resuspended in 20 mL lysis buffer per L cell culture (50 mM Tris pH 8.0, 350 mM NaCl, 30 mM imidazole, 10% glycerol) and stored at –80 °C until purification. Cell suspensions were thawed on ice, supplemented with 1 mM PMSF, 2.5 mM β-mercaptoethanol, lysozyme (20 µg/mL) and DNase I (0.1 mg/mL). Cells were lysed by sonication and the suspensions were cleared by centrifugation (16,000 x g, 20 min, 4 °C) and filtration (0.45 µm membrane filter). Supernatants were subjected to gravity GST affinity purification using lysis buffer. GST-Ube2K was cleaved on Glutathione Sepharose 4 Fast Flow by GST-3C protease. Samples were portioned into aliquots, flash-frozen in liquid N_2 and could be stored at –80 °C for months without noticeable loss of activity.

5.4 Expression of GST-3C protease[18]

Chemically competent BL21 CodonPlus(DE3) RIL for expression, were heat-shock transformed with the plasmids. Single colonies were used to inoculate overnight precultures in selective lysogeny broth (LB) Miller medium. Following 1:100 dilution with fresh selective LB Miller medium with 0.2% glucose, cultures were grown in baffled shake flasks at 37 °C until an OD600 of approximately 0.6 was reached. Protein expression was induced by addition of isopropyl β-D-1-thiogalactopyranoside at a final concentration of 1 mM. Expression was carried out for 3 h at 30 °C, Cells were collected by centrifugation (4,500 x g, 30 min, 4 °C), resuspended in 20 mL lysis buffer per L cell culture (50 mM Tris pH 8.0, 100 mM NaCl, 10% glycerol) and stored at –80 °C until purification. Cell suspensions were thawed on ice, supplemented with 1 mM PMSF, 2.5 mM β-mercaptoethanol, lysozyme (20 µg/mL) and DNase I (0.1 mg/mL). Cells were lysed by sonication and the suspensions were cleared by centrifugation (16,000 x g, 20 min, 4 °C) and filtration (0.45 µm membrane filter). Supernatants were subjected to gravity GST affinity purification using lysis buffer and elution buffer (lysis buffer containing 10 mM glutathione) to obtain GST-3C protease. GST-3C protease was dialysed to 50 mM Tris pH 7.5 at rt, 150 mM NaCl, 1 mM TCEP after GST-tag purification. Samples were portioned into aliquots, flash-frozen in liquid N_2 and could be stored at –80 °C for months without noticeable loss of activity.

5.5 Expression of YUH1

Chemically competent BL21 CodonPlus(DE3) RIL for expression, were heat-shock transformed with the plasmids. Single colonies were used to inoculate overnight precultures in selective lysogeny broth

(LB) Miller medium. The proteins were expressed in autoinduction medium. 1:1000 dilution of overnight precultures were added and cells were grown overnight at 37 °C. Cells were collected by centrifugation (4,000 x g, 25 min, 4 °C), resuspended in 20 mL lysis buffer per L cell culture (50 mM Tris pH 8.0, 350 mM NaCl, 30 mM imidazole, 10% glycerol) and stored at –80 °C until purification. Cell suspensions were thawed on ice, supplemented with 1 mM PMSF, 1 mM DTT, lysozyme (20 µg/mL) and DNase I (0.1 mg/mL). Cells were lysed by sonication and the suspensions were cleared by centrifugation (8,000 x g, 30 min, 4 °C) and filtration (0.45 µm membrane filter). Supernatants were subjected to gravity Ni-NTA affinity purification using lysis buffer and elution buffer (lysis buffer containing 400 mM imidazole and 1 mM DTT), to obtain His-tagged proteins. His-YUH1 was dialyzed to 20 mM HEPES pH 7.5, 100 mM NaCl, 1 mM DTT at 4 °C. Samples were concentrated and portioned into aliquots, flash-frozen in liquid N_2 and could be stored at –80 °C for months without noticeable loss of activity.

6 Ubiquitin chain elongation

6.1 Enzymatic ligation

6.1.1 On-resin synthesis (Figure 4, Figure S2, S3, and S4)

Ub chains were synthesized on Ni-NTA resin (Ni-NTA His Bind resin, Novagen®) in 50 mM HEPES 50 mM NaCl pH 7.5 (ubiquitylation buffer). Ub^A or Ub chain (loaded on 500 μL of Ni-NTA slurry), Ub^D (200 μM, 2 equiv to the initial Ub^A), Uba1 (0.5 μM), Ubc13/Mms2 (20 μM), Mg-ATP (5 mM) and TCEP (200 μM) were mixed on ice and the resulting solution was incubated at 37 °C. After 16 h, the solid phase was washed with washing buffer. The small amount of the resin was taken and suspended with elution buffer for analytical purpose.

Ubiquitylation buffer: 50 mM HEPES 50 mM NaCl pH 7.5

Washing buffer: 100 mM HEPES, 500 mM NaCl, 30 mM imidazole pH 7.5

Elution buffer: 100 mM HEPES, 500 mM imidazole pH 7.5

Abac-deprotection buffer: 100 mM Na phosphate pH 6.0, 150 mM NaCl

6.1.2 Solution-phase synthesis for Ub tetramers (Figure S5, S6, and S8.)

Ub chains were synthesized in 50 mM HEPES 50 mM NaCl pH 7.5. Ub^A (100 μM), Ub^D (200 μM, 2 equiv to the initial Ub^A), Uba1 (0.25 μM), Ubc13/Mms2 (20 μM), Mg-ATP (5 mM) and TCEP (200 μM) were mixed on ice and the resulting solution was incubated at 37 °C. After 16 h, the unpurified mixture was treated with Ni-NTA resin. The resin was washed a few times with washing buffer and the products were eluted from resin with elution buffer. The elution fraction was subjected to a desalting column (HiTrap™ Desalting 5 mL, performed on an ÄKTA pure chromatography system at 4 °C) pre-equilibrated with Abac-deprotection buffer. To the resulting buffer-exchanged sample was added 20 mM PLP. After incubating the mixture at 37 °C until the deprotection was finished, the dimer was purified by size exclusion column (SEC, Superdex 75 3.2/300 column, performed on an ÄKTA pure chromatography system at 4 °C using ubiquitylation buffer).

The resulting dimer solution was subjected to another solution-phase ubiquitylation reaction as described above. After Ni-NTA affinity purification and buffer exchange, the Ub trimer was treated with Ub^D (200 μM, 2 equiv to the initial Ub^A), Uba1 (0.25 μM), Ube2K (20 μM), Mg-ATP (5 mM) and TCEP (200 μM) to obtain branched/heterotypic chains.

6.2 PLP mediated deprotection

Deprotection of Abac on Ub chains were performed either on Ni-NTA resin or in solution phase. The Ub oligomer was washed by washing buffer and 20 mM PLP in Abac-deprotection buffer was added. The resin solution was incubated at 37 °C until full conversion (typically 1-2 days). The fresh PLP

solution was further added in case the conversion was not sufficient and the mixture was reacted for another 16 h. The reaction was monitored by LC-MS.

6.3 $NaIO_4$ mediated deprotection

Deprotection of Aboc on Ub chains were performed either on Ni-NTA resin or in solution phase after tag cleavage. The Ub oligomer was added 1 mM $NaIO_4$ in 200 mM Na borate pH 8.5. After incubated at rt for 30 min, 20 mM Tris pH 9.0 was added to the mixture to quench the excess $NaIO_4$. The resin solution was further incubated at 37 °C for 16 h. The reaction was monitored by LC-MS.

6.4 YUH1 tag cleavage (Figure 4)

The resulting Abac-deprotected Ub_5 branched chain was eluted from the resin by treating the resin with 4 μM YUH1 in washing buffer at 37 °C for 16 h. The flow through was collected and analyzed to confirm tag-cleaved Ub oligomers.

6.5 Estimation of ubiquitylation conversions

Ub chains were separated and visualized by SDS-PAGE as described in 2.12 and 2.13. The band intensity was quantified using Image Lab 6.1 (BIO-RAD). The obtained intensity was converted to the adjusted intensity by dividing the intensity by the corresponding molecular weight. The molar ratio was calculated by dividing the adjusted intensity of the band of interest by the sum of adjusted intensities in the same lane.

7 NMR spectrum

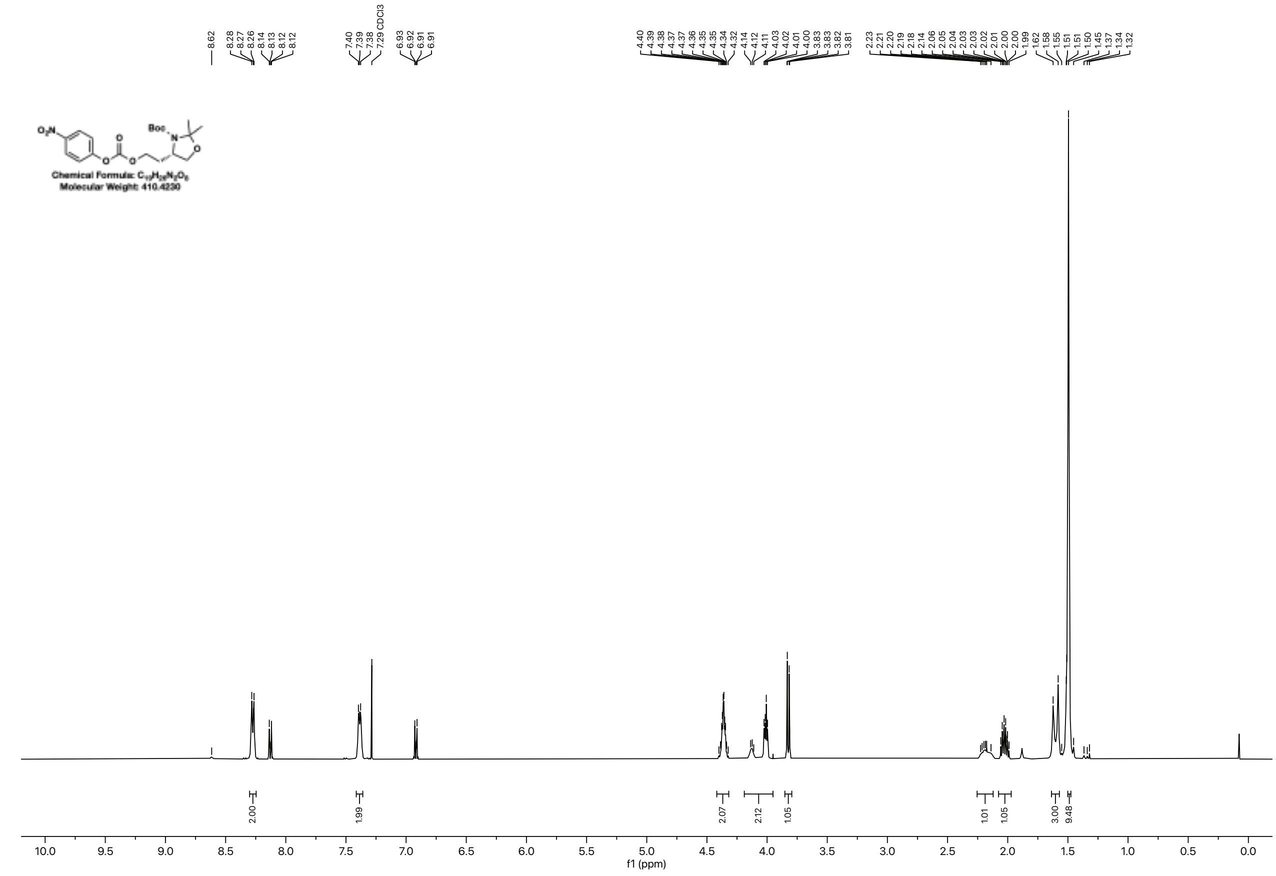

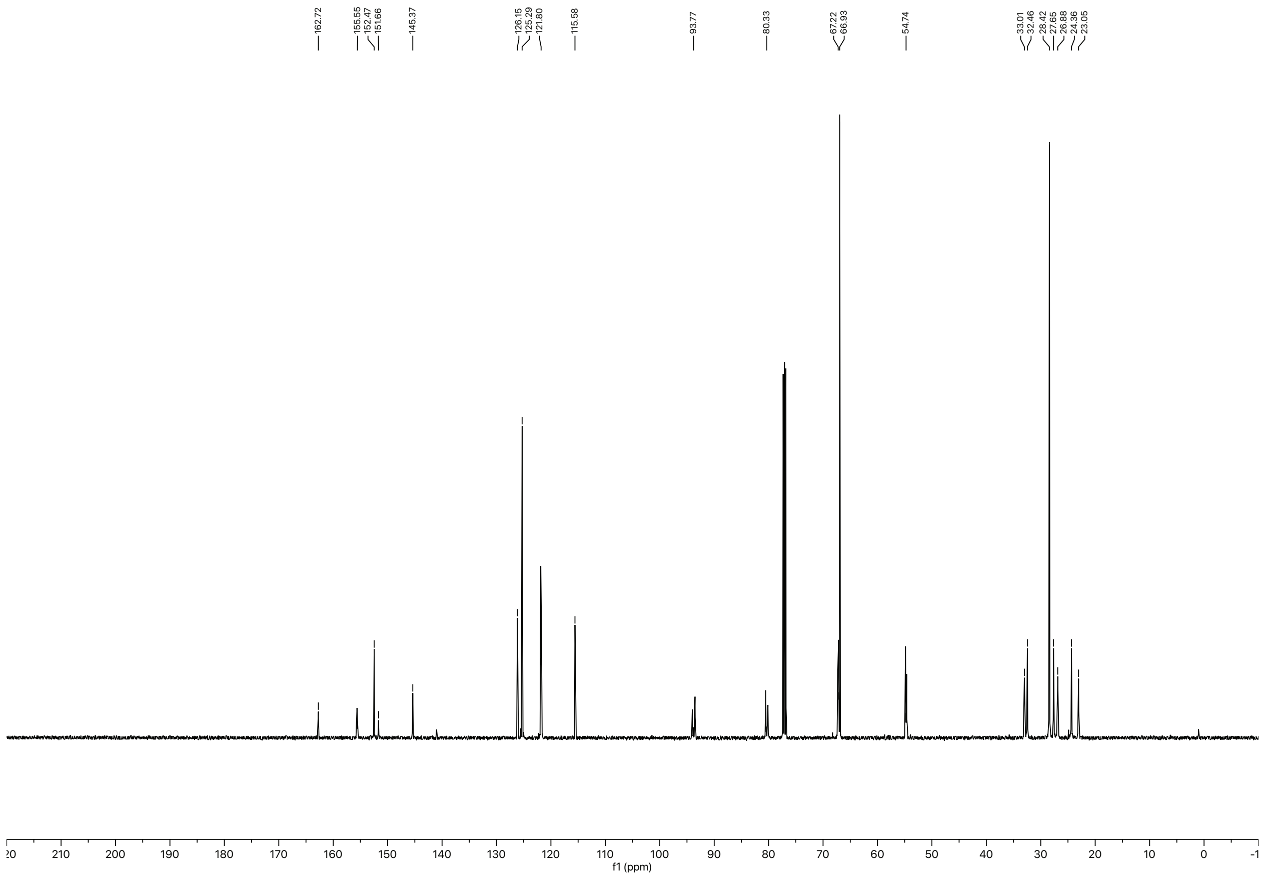

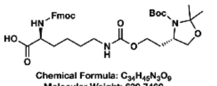

Chemical Formula: $C_{34}H_{45}N_3O_9$
Molecular Weight: 639.7460

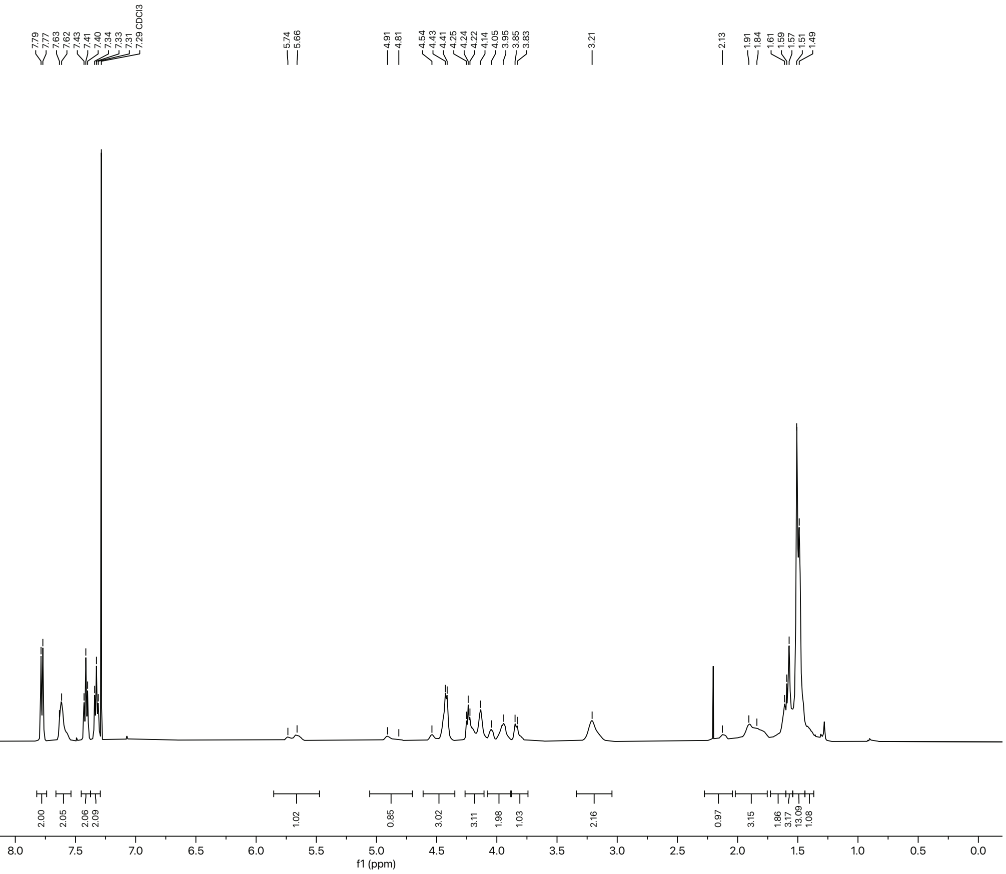

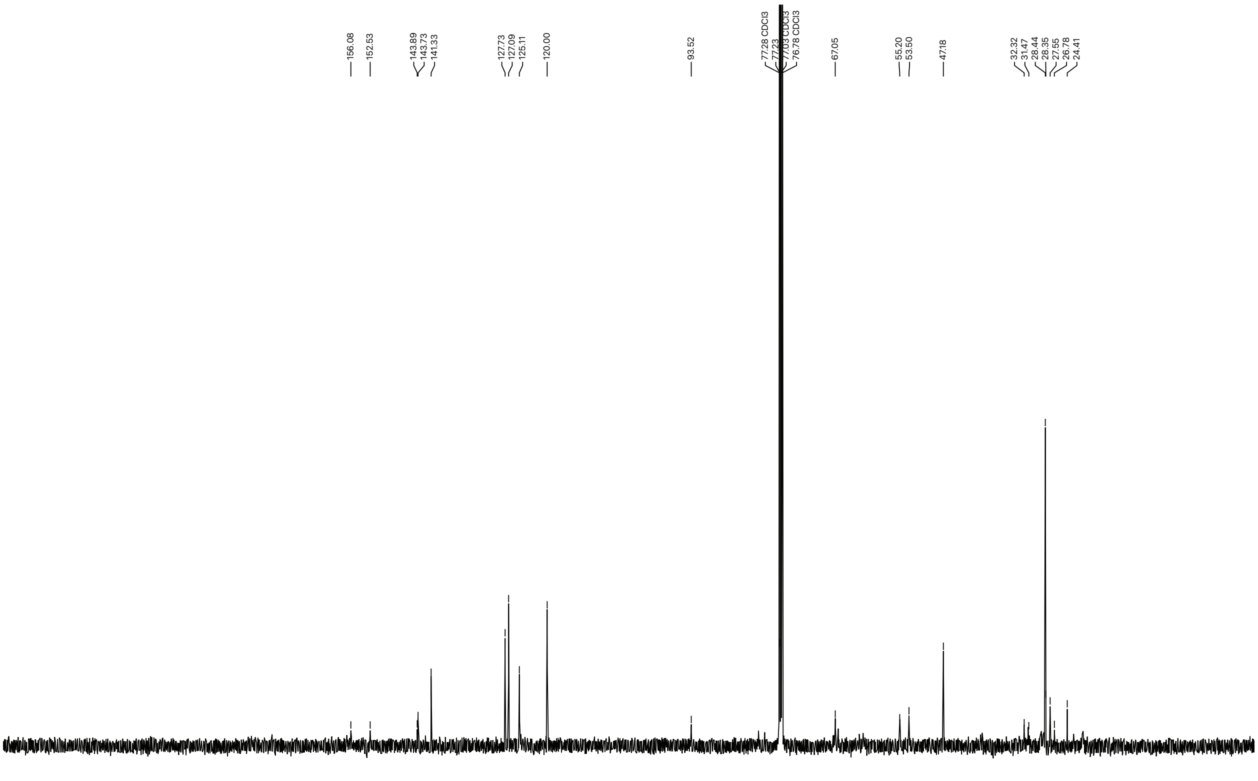

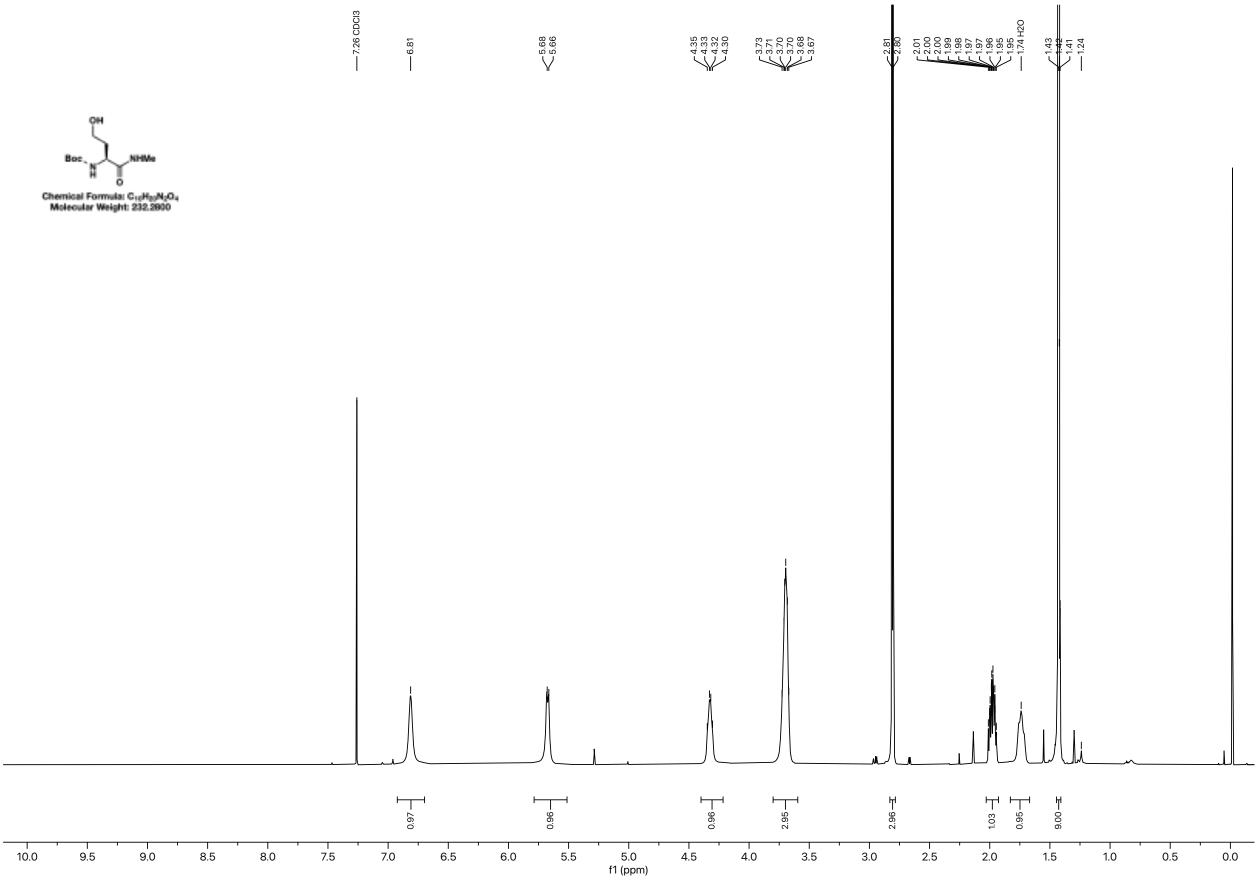
OH
Boc
N
H
NHMe
O
Chemical Formula: C10H20N2O4
Molecular Weight: 232.2800
7.26 CDCl3
6.81
5.68
5.66
4.35
4.33
4.32
4.30
3.73
3.71
3.70
3.70
3.68
3.67
2.81
2.80
2.01
2.00
2.00
1.99
1.98
1.97
1.97
1.96
1.95
1.95
1.74 H2O
1.43
1.42
1.41
1.24
0.97
0.96
0.96
2.95
2.96
1.03
0.95
9.00
10.0 9.5 9.0 8.5 8.0 7.5 7.0 6.5 6.0 5.5 5.0 4.5 4.0 3.5 3.0 2.5 2.0 1.5 1.0 0.5 0.0
f1 (ppm)

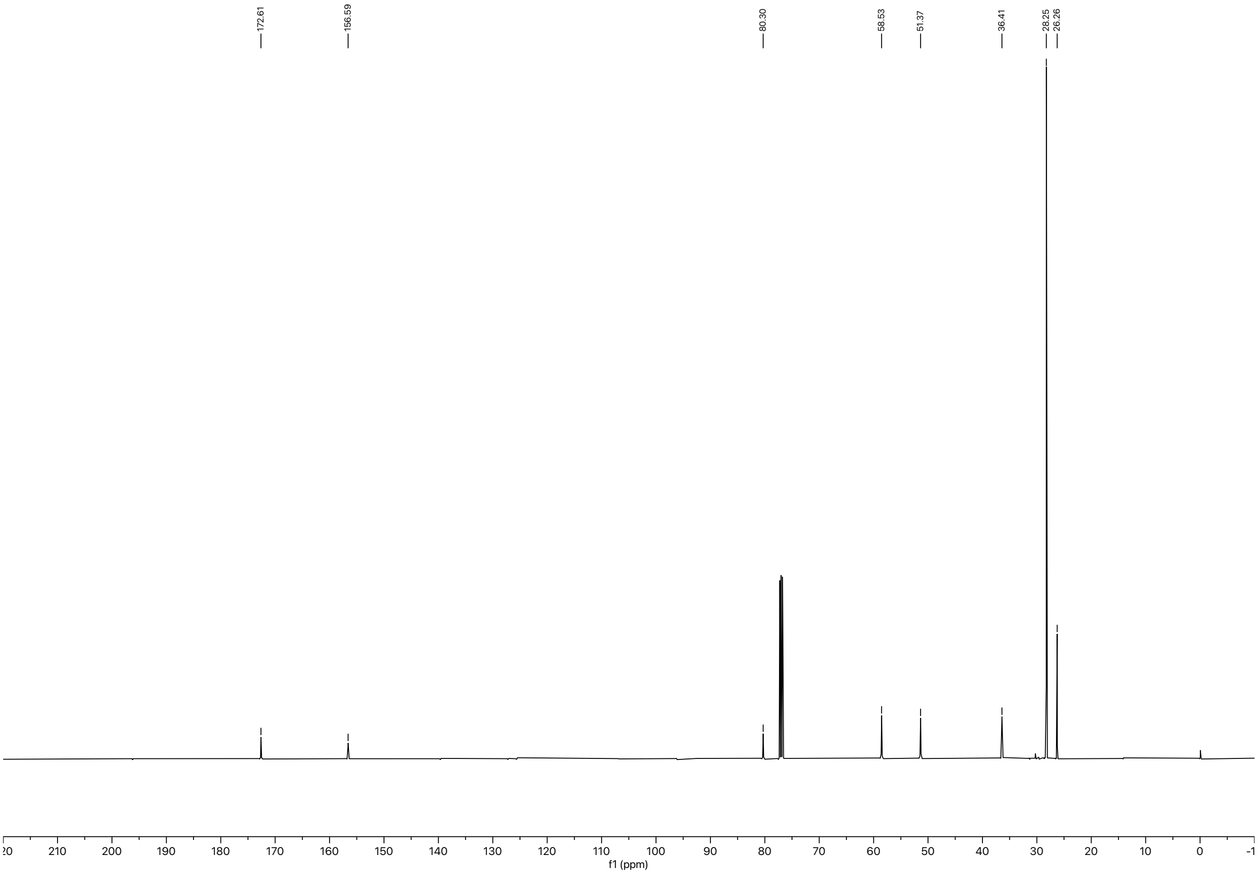
172.61
156.59
80.30
58.53
51.37
36.41
28.25
26.26
210 200 190 180 170 160 150 140 130 120 110 100 90 80 70 60 50 40 30 20 10 0 -1
f1 (ppm)

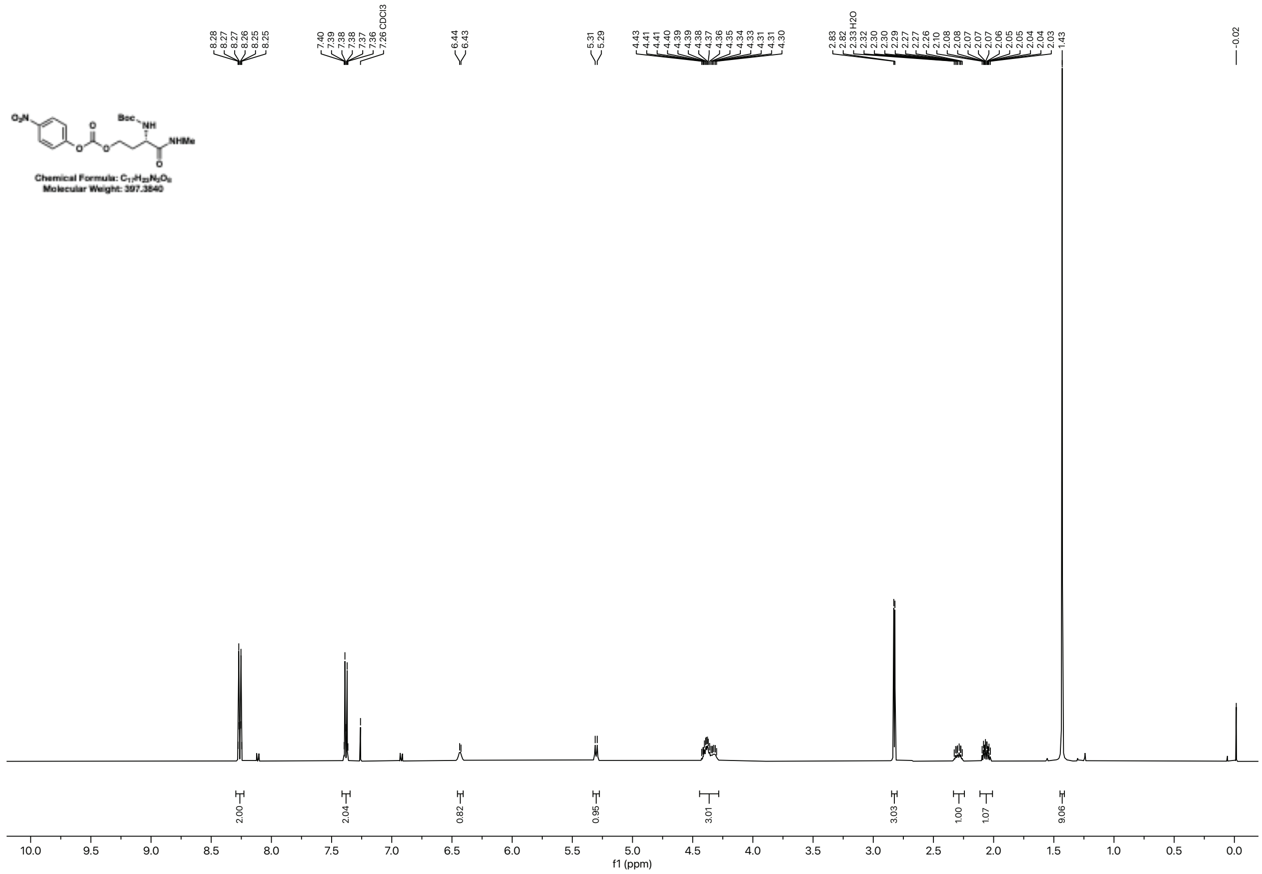

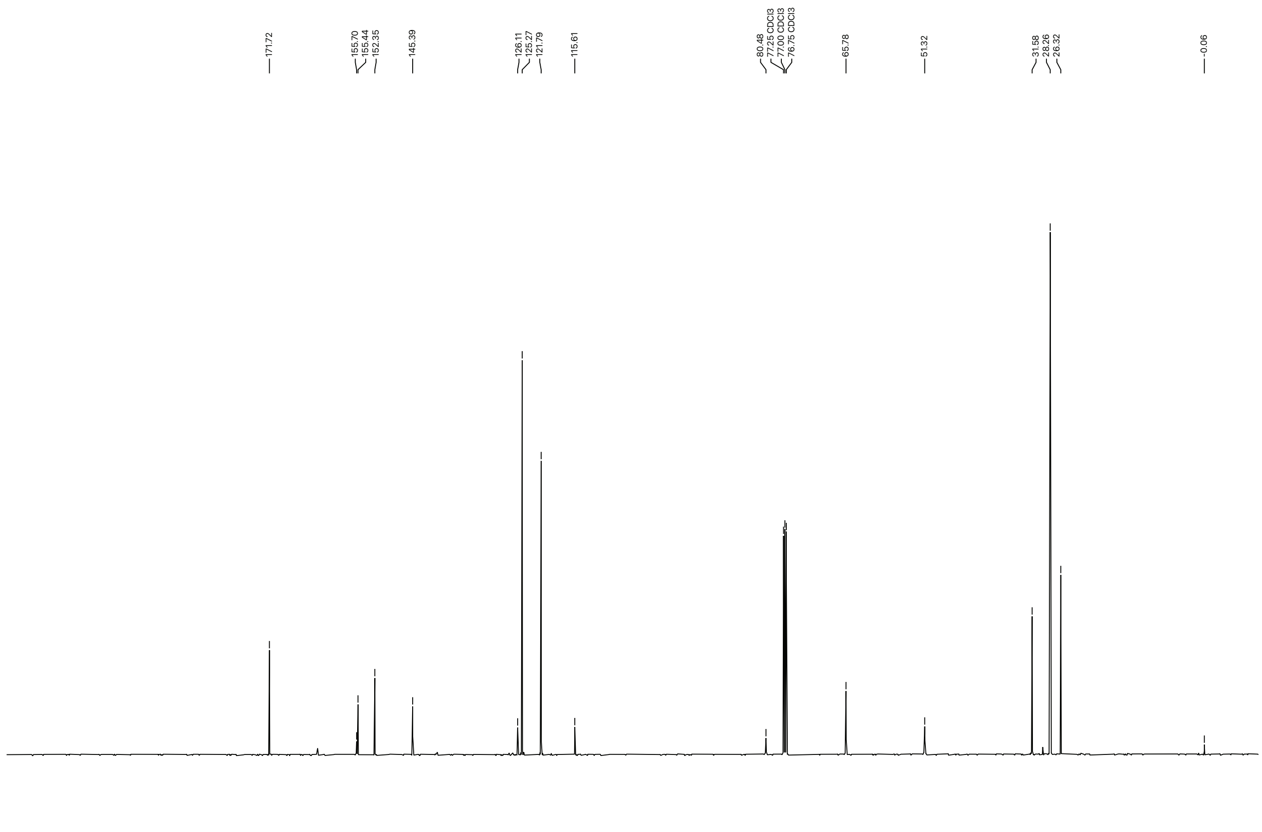

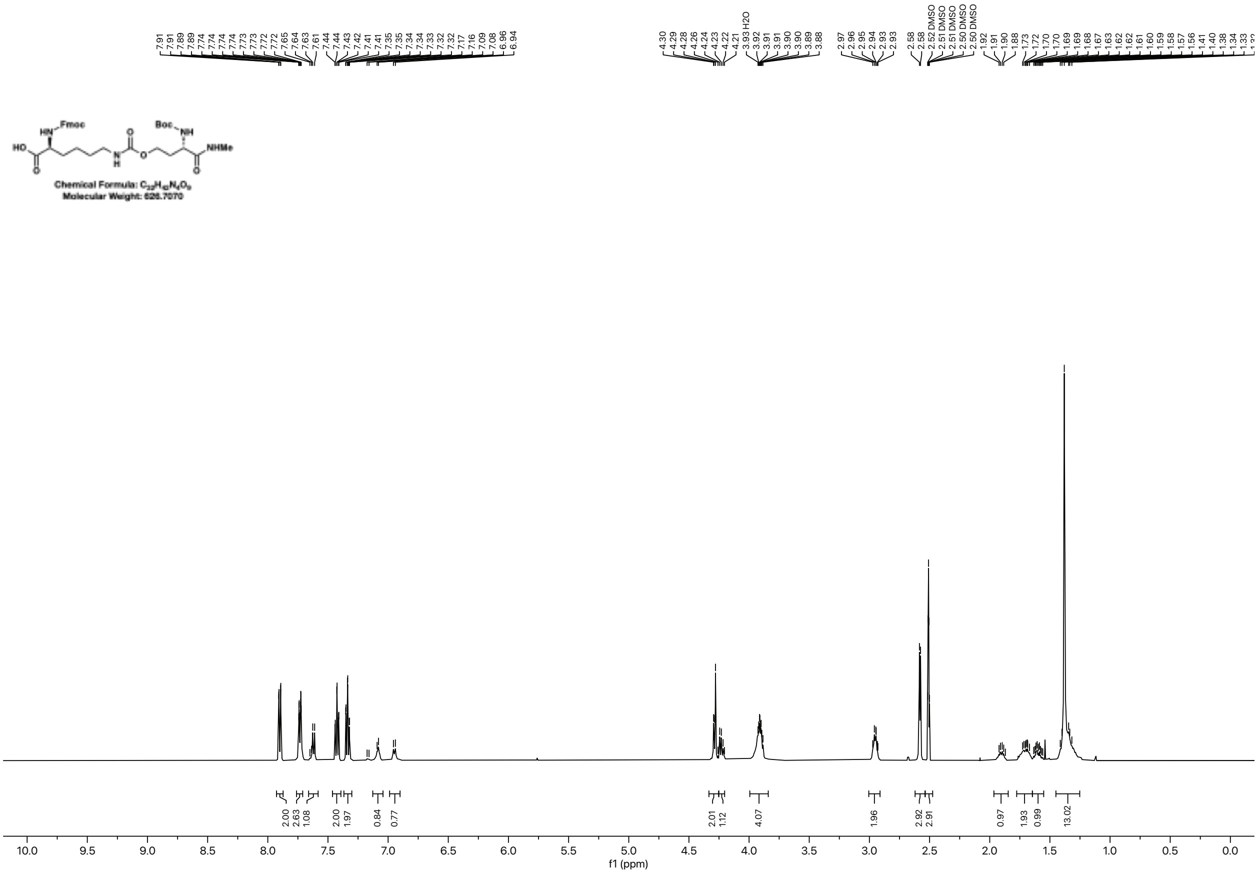
Chemical Formula: $C_{32}H_{42}N_4O_9$
Molecular Weight: 626.7070

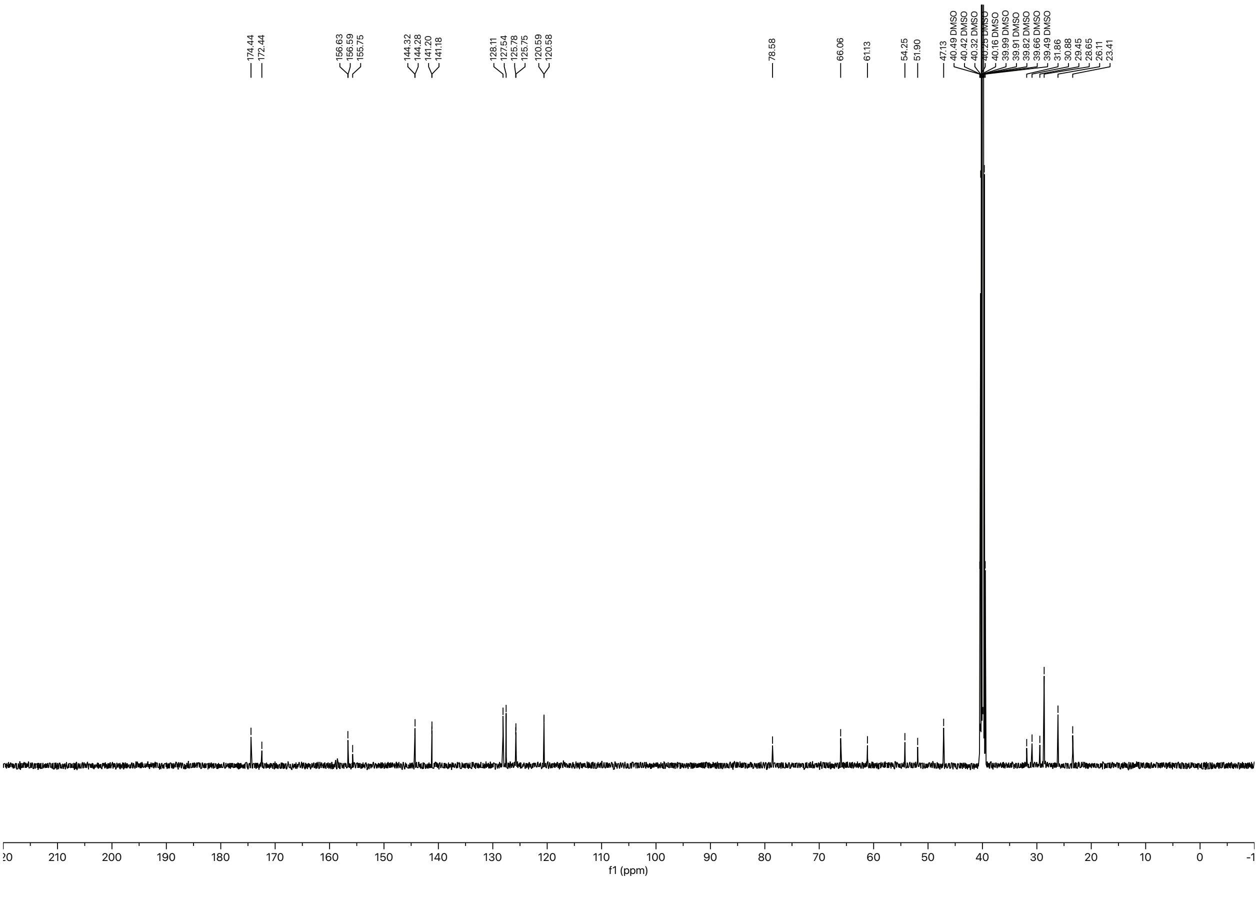

8 Reference

(1) Nakasone, M. A.; Livnat-Levanon, N.; Glickman, M. H.; Cohen, R. E.; Fushman, D. Mixed-Linkage Ubiquitin Chains Send Mixed Messages. *Structure* **2013**, *21* (5), 727–740. https://doi.org/10.1016/j.str.2013.02.019.

(2) Piotrowski, J.; Beal, R.; Hoffman, L.; Wilkinson, K. D.; Cohen, R. E.; Pickart, C. M. Inhibition of the 26 S Proteasome by Polyubiquitin Chains Synthesized to Have Defined Lengths *. *J. Biol. Chem.* **1997**, *272* (38), 23712–23721. https://doi.org/10.1074/jbc.272.38.23712.

(3) Matsumoto, M. L.; Wickliffe, K. E.; Dong, K. C.; Yu, C.; Bosanac, I.; Bustos, D.; Phu, L.; Kirkpatrick, D. S.; Hymowitz, S. G.; Rape, M.; Kelley, R. F.; Dixit, V. M. K11-Linked Polyubiquitination in Cell Cycle Control Revealed by a K11 Linkage-Specific Antibody. *Mol. Cell* **2010**, *39* (3), 477–484. https://doi.org/10.1016/j.molcel.2010.07.001.

(4) Castañeda, C. A.; Liu, J.; Kashyap, T. R.; Singh, R. K.; Fushman, D.; Cropp, T. A. Controlled Enzymatic Synthesis of Natural-Linkage, Defined-Length Polyubiquitin Chains Using Lysines with Removable Protecting Groups. *Chem. Commun.* **2011**, *47* (7), 2026–2028. https://doi.org/10.1039/C0CC04868B.

(5) Kumar, K. S. A.; Spasser, L.; Erlich, L. A.; Bavikar, S. N.; Brik, A. Total Chemical Synthesis of Di-ubiquitin Chains. *Angew. Chem. Int. Ed.* **2010**, *49* (48), 9126–9131. https://doi.org/10.1002/anie.201003763.

(6) Kumar, K. S. A.; Bavikar, S. N.; Spasser, L.; Moyal, T.; Ohayon, S.; Brik, A. Total Chemical Synthesis of a 304 Amino Acid K48-Linked Tetraubiquitin Protein. *Angew. Chem. Int. Ed.* **2011**, *50* (27), 6137–6141. https://doi.org/10.1002/anie.201101920.

(7) Pan, M.; Gao, S.; Zheng, Y.; Tan, X.; Lan, H.; Tan, X.; Sun, D.; Lu, L.; Wang, T.; Zheng, Q.; Huang, Y.; Wang, J.; Liu, L. Quasi-Racemic X-Ray Structures of K27-Linked Ubiquitin Chains Prepared by Total Chemical Synthesis. *J. Am. Chem. Soc.* **2016**, *138* (23), 7429–7435. https://doi.org/10.1021/jacs.6b04031.

(8) Tang, S.; Liang, L.-J.; Si, Y.-Y.; Gao, S.; Wang, J.-X.; Liang, J.; Mei, Z.; Zheng, J.-S.; Liu, L. Practical Chemical Synthesis of Atypical Ubiquitin Chains by Using an Isopeptide-Linked Ub Isomer. *Angew. Chem. Int. Ed.* **2017**, *56* (43), 13333–13337. https://doi.org/10.1002/anie.201708067.

(9) Castañeda, C.; Liu, J.; Chaturvedi, A.; Nowicka, U.; Cropp, T. A.; Fushman, D. Nonenzymatic Assembly of Natural Polyubiquitin Chains of Any Linkage Composition and Isotopic Labeling Scheme. *J. Am. Chem. Soc.* **2011**, *133* (44), 17855–17868. https://doi.org/10.1021/ja207220g.

(10) Virdee, S.; Ye, Y.; Nguyen, D. P.; Komander, D.; Chin, J. W. Engineered Diubiquitin Synthesis Reveals Lys29- Isopeptide Specificity of an Otu Deubiquitinase. *Nat. Chem. Biol.* **2010**, *6* (10), 750–757. https://doi.org/10.1038/nchembio.426.

(11) Harmand, T. J.; Murar, C. E.; Bode, J. W. Protein Chemical Synthesis by α-Ketoacid–Hydroxylamine Ligation. *Nat. Protoc.* **2016**, *11* (6), 1130–1147. https://doi.org/10.1038/nprot.2016.052.

(12) Pichler, A.; Knipscheer, P.; Oberhofer, E.; van Dijk, W. J.; Körner, R.; Olsen, J. V.; Jentsch, S.; Melchior, F.; Sixma, T. K. SUMO Modification of the Ubiquitin-Conjugating Enzyme E2-25K. *Nat. Struct. Mol. Biol.* **2005**, *12* (3), 264–269. https://doi.org/10.1038/nsmb903.

(13) Rabe, B. A.; Cepko, C. A Simple Enhancement for Gibson Isothermal Assembly. bioRxiv June 15, 2020, p 2020.06.14.150979. https://doi.org/10.1101/2020.06.14.150979.

(14) Ozinskas, A. J.; Rosenthal, G. A. Synthesis of L-Canaline and .Gamma.-Functional 2-Aminobutyric Acid Derivatives. *J. Org. Chem.* **1986**, *51* (26), 5047–5050. https://doi.org/10.1021/jo00376a001.

(15) Gu, X.-S.; Yu, N.; Yang, X.-H.; Zhu, A.-T.; Xie, J.-H.; Zhou, Q.-L. Enantioselective Hydrogenation of Racemic α-Arylamino Lactones to Chiral Amino Diols with Site-Specifically Modified Chiral Spiro Iridium Catalysts. *Org. Lett.* **2019**, *21* (11), 4111–4115. https://doi.org/10.1021/acs.orglett.9b01290.

(16) Paintner, F. F.; Allmendinger, L.; Bauschke, G.; Klemann, P. Highly Efficient Approach to Orthogonally Protected (2 S,4 R)- and (2 S,4 S)-4-Hydroxyornithine. *Org. Lett.* **2005**, *7* (7), 1423–1426. https://doi.org/10.1021/ol0503182.

(17) Studier, F. W. Stable Expression Clones and Auto-Induction for Protein Production in E. Coli. *Structural Genomics: General Applications*, 2014, 17–32. https://doi.org/10.1007/978-1-62703-691-7_2.

(18) Raran-Kurussi, S.; Waugh, D. S. A Dual Protease Approach for Expression and Affinity Purification of Recombinant Proteins. *Anal. Biochem.* **2016**, *504*, 30–37. https://doi.org/10.1016/j.ab.2016.04.006.
