# Supplementary material for: Biocompatible Lysine Protecting Groups for the Chemoenzymatic Synthesis of K48/K63 Heterotypic and Branched Ubiquitin Chains
Source: ACS Cent Sci. 2023 Jul 15;9(8):1633–41. doi: 10.1021/acscentsci.3c00389 (PMC10450881; doi:10.1021/acscentsci.3c00389)
Supplement: Supplementary file 2 — oc3c00389_si_002.pdf [file oc3c00389_si_002.pdf]

oc-2023-00389c.R1

Name: Peer Review Information for "Biocompatible lysine protecting groups for the chemoenzymatic synthesis of K48/K63 heterotypic and branched ubiquitin chains"

First Round of Reviewer Comments

Reviewer: 1

Comments to the Author

Ubiquitin chains of distinct topologies play an essential role in cellular activities by regulating the stability, interactions, or localization of substrate proteins. Recent studies found that heterotypic and branched chains also elicit a variety of biological outputs, further expanding the versatility, specificity, and complexity of ubiquitin associated signaling. However, the understanding of heterotypic and branched chains has often been hampered by limited access to prepare them. In this manuscript, Mikami et al. developed an expedient method for on-resin chemo-enzymatic synthesis of heterotypic and branched ubiquitin chains by introducing two biocompatible lysine protecting groups, Abac and Aboc, which can be deprotected under refolding conditions. The utility of the method was exemplified by synthesis of several architecture-defined K48/63 branched Ub chains using K48-specific E2 E2-25K, and K63-specific E2 Ubc13/Mms2. This method, in principle, enables construction of any branching site by simply changing the donor or acceptor Ub moiety. In my opinion, through introducing more chain-specific E2/E3 enzymes and improving the coupling efficiency, this shall become a robust method to obtain defined ubiquitin chains. Therefore, this work deserves a publication in ACS Central Science after addressing following concerns.

(1) The authors postulated that the protecting groups might impair the binding of E2, thereby affecting the conversion. Is there a possibility that the liquid-solid heterogeneous reaction lowered the E2 catalyzed coupling efficiency?

(2) Whether increasing the amount of donor Ub to 4eq. or more as used in SPPS would improve the conversion efficiency, have the authors tested?

(3) The conversion efficiency of each chain-elongating coupling reaction and the overall yield for each branched Ub chain should be provided.

(4) Figure 3A and all figures in the Supporting Information should be referred in the main text.

(5) The caption for Figure 4 (B), (C) and (E) should be revised as they were all the same “SDS-PAGE gel after Coomassie staining” in the current form.

(6) In Figure S1, the graph for the “IsoUb strategy” is inappropriate and should revised as no IsoUb unit for K48, and K63 in the strategy

(7) The “GOPAL” strategy is developed by Chin and Komander, not Rape.

(8) The caption of Figure S3B is inconsistent with the graph, and there were two “(B)” for S3.

Reviewer: 2

Comments to the Author

In this manuscript, Mikami and colleagues report a new method to generate site specific and homogeneous free branched polyubiquiton chains. This method is based on the design and utilization of orthogonal cleavable protection of the branching sites. One is a pyridoxal 5’-phosphate (PLP) and the other, an aminobutanol carbamate. These efforts include the design of protecting groups that maintain the overall properties of folded Ub monomers and allow the chemoenzymatic addition of ubiquitin. This method yields atypical K48-K63 branched ubiquitin chains. The synthetic routes are well-described, the products show high purity, and the synthesis of several different atypical chain production were demonstrated however, there is very limited discussion of yield.

Overall, this work nicely demonstrates the use of chemoenzymatic strategy to make polymers of ubiquitin, but not a conceptual leap from existing methods that achieve similar chains (albeit a bit shorter). Moreover, the work has limited impact as the authors do not show the utility of the method by, for example, creating a modified protein substrate, testing deubiquitinating enzymes activity on the branched chains or specific polyubiquitin-binding proteins.

Reviewer: 3

Comments to the Author

The authors describe a new synthetic strategy to generate specific oligomers of ubiquitin (Ub). Advances of this type are very important for unraveling the complexities of Ub-based signaling, which is widely employed in biological systems. The Ub "language" is complex because Ub units can be linked to one another at multiple distinct Lys residues on the surface of this protein, which leads to myriad of geometric possibilities. Strategies that deliver specific Ub oligomers remain a high priority in this field, because the available tools are limited.

The authors' approach relies on two features, Ub-conjugating enzymes with specific Lys preferences, which have been employed by others, and protecting groups for Lys side chains that are tailored for use in aqueous solution, which are new. The authors' protecting group design is clever and successful. The specific oligomers that they are able to prepare, which include branched examples, demonstrate the power of their method.

This work should appeal to a broad audience. I recommend that the manuscript be accepted after the authors have considered two minor concerns.

First, the figures are generally very helpful, but I believe there is a small error in Fig. 1A. At the upper right, I believe that "- CO2" should be "- NH3".

Second, the authors cite relevant synthetic efforts from several groups, but they should also cite work from Strieter et al., for example, Org. Lett. 2019, 21, 6790.

Author's Response to Peer Review Comments:

Please see attached response letter.

Dear Editor,

Thank you for the prompt and positive review of our manuscript, “Biocompatible lysine protecting groups for the chemoenzymatic synthesis of K48/K63 heterotypic and branched ubiquitin chains”. We have carefully considered the input from the expert reviewers and have revised our manuscript accordingly. Point-by-point responses to the specific concerns are provided below, and a track-changes versions of the manuscript and SI, highlighting the modifications made, are included in the online submission.

The revisions have been approved by all authors and modifications in the manuscript have been highlighted in red using the tracking system.

Sincerely,

Jeffrey

Reviewer(s)' Comments to Author:

Reviewer: 1

(1) "The authors postulated that the protecting groups might impair the binding of E2, thereby affecting the conversion. Is there a possibility that the liquid-solid heterogeneous reaction lowered the E2 catalyzed coupling efficiency?"

The coupling efficiency of the on-resin reaction was similar to that of solution-phase reaction. We added the following sentences in the Discussion section.

> "We found that the efficiency of conjugation on solid support was comparable to that in solution phase."

(2) Whether increasing the amount of donor Ub to 4eq. or more as used in SPPS would improve the conversion efficiency, have the authors tested?

In initial studies, we tried using more Ub^D for the conjugation reactions. However, the conversion was similar to that when 2 equiv Ub^D was used (200 μM). Furthermore, Ub^D precipitates during the reaction when the concentration is higher than 250 μM.

(3) The conversion efficiency of each chain-elongating coupling reaction and the overall yield for each branched Ub chain should be provided.

We added the conversions in Figure 4 and Figures S3 to S8 according to the densitometry of the SDS-PAGE gels.

We also added an "Estimation of ubiquitylation conversions" section on page S36 in the Supplementary Information.

(4) Figure 3A and all figures in the Supporting Information should be referred in the main text.

Figure 3A is referred on page 7.
Figure S2 is referred on page 9.
Figure S3 to S8 are referred on page 11.

(5) The caption for Figure 4 (B), (C) and (E) should be revised as they were all the same "SDS-PAGE gel after Coomassie staining" in the current form.

We added the additional explanations to Figure 4B, 4C, and 4E.

(6) In Figure S1, the graph for the "IsoUb strategy" is inappropriate and should revised as no IsoUb unit for K48, and K63 in the strategy

We revised the scheme in Figure S1 according to the literature (10.1002/ange.201708067). They have prepared K11/K48 branched chains by combining K11 isoUb segment and K48 isoUb segment (Figure 2 in their paper)

(7) The "GOPAL" strategy is developed by Chin and Komander, not Rape.

The caption of Figure S1 was corrected.

(8) The caption of Figure S3B is inconsistent with the graph, and there were two "(B)" for S3.

The caption of Figure S3 was corrected.

Reviewer: 3

(1) First, the figures are generally very helpful, but I believe there is a small error in Fig. 1A. At the upper right, I believe that "- CO2" should be "- NH3".

We have modified Figure 2A to clarify the removal of the protecting group.

(2) The authors cite relevant synthetic efforts from several groups, but they should also cite work from Strieter et al., for example, Org. Lett. 2019, 21, 6790.

We added the reference on page 3 of the manuscript with the sentence shown below.

> "Strieter et al. successfully synthesized branched triubiquitin activity-based probes by a combination of enzymatic conjugation and Cys modification reaction."

Formatting

(1) GENERAL REF FORMATTING: Periodical references should contain authors' surnames followed by initials, article title, journal abbreviation, year, volume number, and page range. Refs with more than 10 authors should list the first 10 and then be followed by "et al."

Doi links were deleted. All the reference contains authors' surnames followed by initials, article title, journal abbreviation, year, volume number, and page range in this order.

(2) Web sources must include access date.

The accessed date was added to the reference 33.

(3) TOC MISSING: Provide a TOC image per journal guidelines (3.25 in. × 1.75 in. (8.25 cm

× 4.45 cm) ; on the last page of the Manuscript) with the heading "TOC Graphic" above the graphic.

We have included the TOC Graphic at the end of the manuscript and as a separate graphics file.

(4) SYNOPSIS MISSING: The synopsis should be no more than 200 characters (including spaces) and should reasonably correlate with the TOC graphic. The synopsis is intended to explain the importance of the article to a broader readership across the sciences. Please place your synopsis in the manuscript file after the TOC graphic.

A synopsis was added at the last page of the main manuscript.
